# Electrochemical Synthesis of 3-Selenyl-Chromones via Domino C(sp^2^)-H Bond Selenylation/Annulation of Enaminones

**DOI:** 10.3390/molecules31020391

**Published:** 2026-01-22

**Authors:** João M. Brito, Isabella M. e Oliveira, Cassio A. O. Moraes, Alex R. Schneider, Tiago E. A. Frizon, Giancarlo V. Botteselle, Vijay P. Singh, André L. Stein, Gleison A. Casagrande, Giuseppe A. Camara, Antonio L. Braga, Jamal Rafique, Sumbal Saba

**Affiliations:** 1 Laboratório de Síntese Sustentável e Organocalcogênio (LABSO), Instituto de Química (IQ), Universidade Federal de Goiás–UFG, Goiania 74690-900, Brazil; 2 Instituto de Química (INQUI), Universidade Federal do Mato Grosso do Sul–UFMS, Campo Grande 79074-460, Brazilgiuseppe.silva@ufms.br (G.A.C.); 3 Departamento de Química, Universidade Federal de Santa Catarina–UFSC, Florianopolis 88040-900, Brazilbraga.antonio@ufsc.br (A.L.B.); 4 Departamento de Química, Universidade Estadual do Centro-Oeste–UNICENTRO, Guarapuava 85819-110, Brazil; 5 Department of Chemistry & Centre of Advanced Studies in Chemistry, Panjab University, Sector-14, Chandigarh 160 014, India; 6 Laboratório de Pesquisa de Produtos Naturais, Instituto de Química, Universidade Federal de Mato Grosso–UFMT, Cuiaba 78060-900, Brazil

**Keywords:** diselenide, seleno-cyclization, domino reaction, electrosynthesis, enaminone, chromone

## Abstract

Herein, we disclose a highly efficient pathway toward 3-selenylated chromone derivatives via electrosynthesis domino C(sp^2^)-H bond selenylation/cyclization/deamination of 2-hydroxyaryl enaminones with diselenides. This method showed mild conditions, easy operation, a wide substrate scope, and good functional group tolerance. Furthermore, this electrosynthesis strategy was amenable to scaling up the reaction. Additionally, the preliminary experiments revealed that this reaction probably proceeded via a cation pathway instead of a radical pathway.

## 1. Introduction

**Figure 1:**
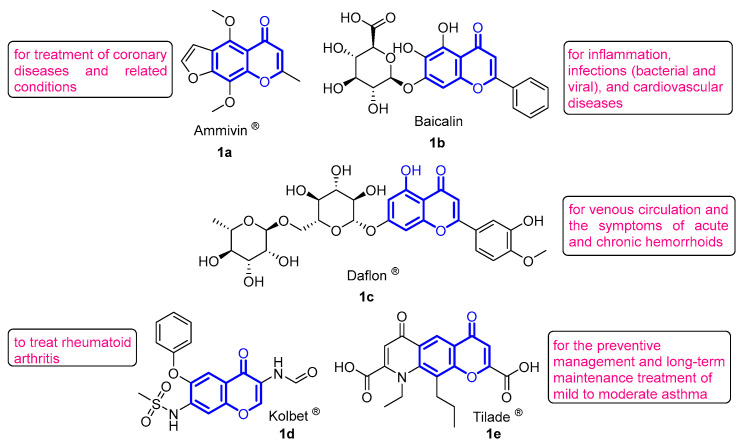
Commercially available pharmaceuticals with chromone nucleus in core structure.

Chromones are commonly found in natural sources [1,2,3]. Their nucleus is found in the vast majority of natural compounds [4,5] and displays a wide range of pharmacological activities, like antiallergic [6], anti-Alzheimer [7], anti-bacterial [8], anti-cancer [9], antidiabetic [10], anti-HIV [11,12], immune-stimulatory [13], immunostimulators [14], anti-inflammatory [15], anti-oxidant [16], anti-ulcer [17], antiviral [18], biocidal [19], and wound healing [20] properties. A number of commercially available pharmaceuticals have the chromone nucleus in their core structure [21,22,23,24] (Figure 1), for example, Ammivin **1a**, Baicalin **1b**, Daflon **1c**, Kolbet **1d**, and Tilade **1e** [21,22,23,24]. Besides medicinal properties, chromone derivatives serve as versatile building blocks in materials science [25,26,27]. Consequently, due to their structural diversity, biological activity, and synthetic utility, these compounds have attracted significant interest among the synthetic science community [28,29,30,31,32,33,34,35].

Similarly, research in the field of organoselenium chemistry has garnered substantial scientific interest over recent decades [36,37,38,39,40,41]. Organoselenium compounds exhibit a remarkably broad and potent pharmacological profile, demonstrating efficacy in diverse areas [42,43,44,45,46,47,48,49,50,51]. This wide-ranging bioactivity is largely attributable to the distinctive reactivity of the C–Se bond, coupled with the relative ease with which these molecules can be accessed through a multitude of well-developed synthetic methodologies [52]. Besides their biological roles, they serve as versatile synthetic intermediates [52], as ligands [53], as catalysts in organic transformations [54,55], and for use in enantioselective synthesis [56,57]. The applicability of organoselenium compounds further transcends the domains of biology and synthesis, finding significant utility in materials science [58,59,60].

Furthermore, the integration of electrochemistry into modern organic synthesis represents a paradigm shift toward more sustainable and precise chemical bond formation/cleavage. Electrochemistry contributes uniquely to green chemistry goals by enhancing atom economy, reducing toxic waste, and enabling the use of renewable electricity as a primary energy source [61,62,63,64,65,66,67]. In this regard, electrochemistry is revolutionizing the formation of C–Se bonds, a critical motif in organoselenium chemistry, by providing a sustainable and selective synthetic platform [68,69,70].

Considering the therapeutic properties of chromones and the biological importance of organoselenium compounds, several routes have been developed to combine these structures into a single hybrid molecule. In the last decade, significant progress has been made in the one-pot selenylation and chromone annulation of 2-hydroxyaryl enaminones, using a domino process that involves deamination and (sp^2^)–H bond functionalization with various selenium sources [22,24,71,72,73,74,75,76,77,78]. Nevertheless, some of the current synthetic approaches are accompanied by a few drawbacks concerning their applicability and environmental profile. Common issues involve the need for pre-functionalized coupling partners, hazardous solvents, expensive reagents, limited range of compatible substrates, harsh conditions (elevated temperatures and long durations), low atom economy, metal catalysts, malodorous reagents, multi-step operations, etc.

Given the importance of these hybrid compounds, the scientific community is actively pursuing new, sustainable synthetic routes that offer high efficiency. Herein, we report an eco-friendly electrochemical method for synthesizing bioactive 3-selenylchromones. This strategy employs a domino C(sp^2^)-H selenylation/cyclization/deamination of 2-hydroxyaryl enaminones with diselenides. The process uses inexpensive, bench-stable potassium iodide (KI) as the electrolyte in acetonitrile under an open-air atmosphere and concludes within 60 min. This study extends our research program, which is dedicated to developing environmentally benign synthetic processes and introducing chalcogen groups into biologically relevant heterocycles [79,80,81,82,83,84].

This new and alternative electrochemical approach proved to be robust, scalable, and effective for the rapid coupling of various 2-hydroxyaryl enaminones and structurally diverse diorganyl diselenides using potassium iodide under ambient conditions and open-air atmosphere.

## 2. Results and Discussion

To establish the optimal reaction conditions, substrates enaminone **2a** and diphenyl diselenide **3a** were subjected to screening of various parameters (Table 1). Based on our previous experience of electrochemical reactions [82], initial trials were conducted using platinum electrodes in acetonitrile (MeCN) with a stoichiometric amount of electrolyte for 60 min (Table 1, entries 1–7). Potassium iodide (KI) proved to be the most effective electrolyte, providing selenylated chromone **4a** in 92% isolated yield (Entry 5).

In the next step, the stoichiometric amount of electrolyte was analyzed, ranging from 1.5 equiv. to 0.5 equiv. (entry 8 and 9). A lower amount of electrolyte resulted in a poor yield of **4a** (entry 9 vs. 5), while a higher amount led to a lower yield of the desired product (entry 8 vs. 5).

After establishing the KI as an appropriate electrolyte, in the next step, a solvent screen was performed for this transformation (entries 10–14). While MeCN remained optimal, MeOH afforded **4a** in 69% yield (entry 11). Other solvents tested were unsatisfactory. This aligns with the prevalent use of MeCN in electrosynthesis, as its high dielectric constant and stability under thermal and electrochemical conditions are ideal for such reactions [61,62,63,64,65,66,67,68,69,70].

Finally, solvent volume was examined. Reducing the volume to 3 mL impaired the reaction (entry 15 vs. 5), whereas increasing it to 10 mL had no significant effect on the yield (entry 16 vs. 5).

The performance was negatively impacted by altering the electrical current. An increase to 25 mA reduced the yield (entry 17), while a decrease to 15 mA caused a significant decline in efficiency (entry 18). Evaluation of reaction time confirmed 60 min as the optimum, with both 30 and 90 min trials producing inferior results (entry 5 vs. 19–20). Under the conditions for entries 13 and 14, the reaction stalled entirely, allowing for complete recovery of the starting material. Lastly, substituting graphite electrodes rendered the transformation inefficient, yielding poor results or no observable conversion (entries 21–23).

**Scheme 1:**
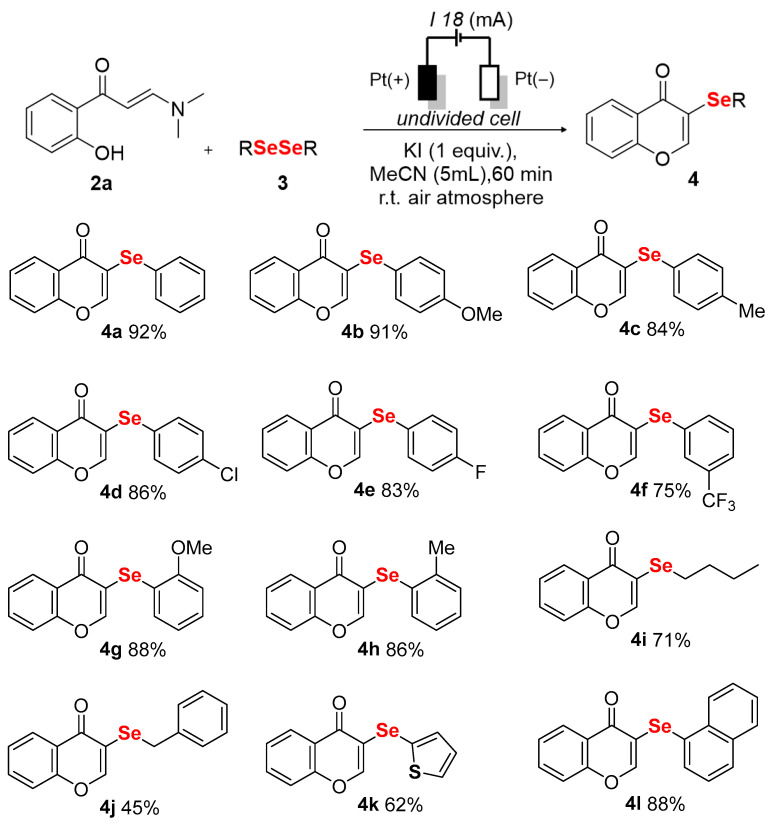
Scope of diorganyl diselenide **3** ^[a],[b]^. Legend: ^[a]^ General reaction conditions: Pt plate electrodes (10 mm × 10 mm × 0.05 mm); (**1a**) (0.25 mmol), (**2a**) (0.25 mmol), KI (0.25 mmol), MeCN (5 mL), 60 min, r.t.; ^[b]^ isolated yields. The red color is the Selenium atom. It highlight that diselenide get added and form the product.

**Scheme 2:**
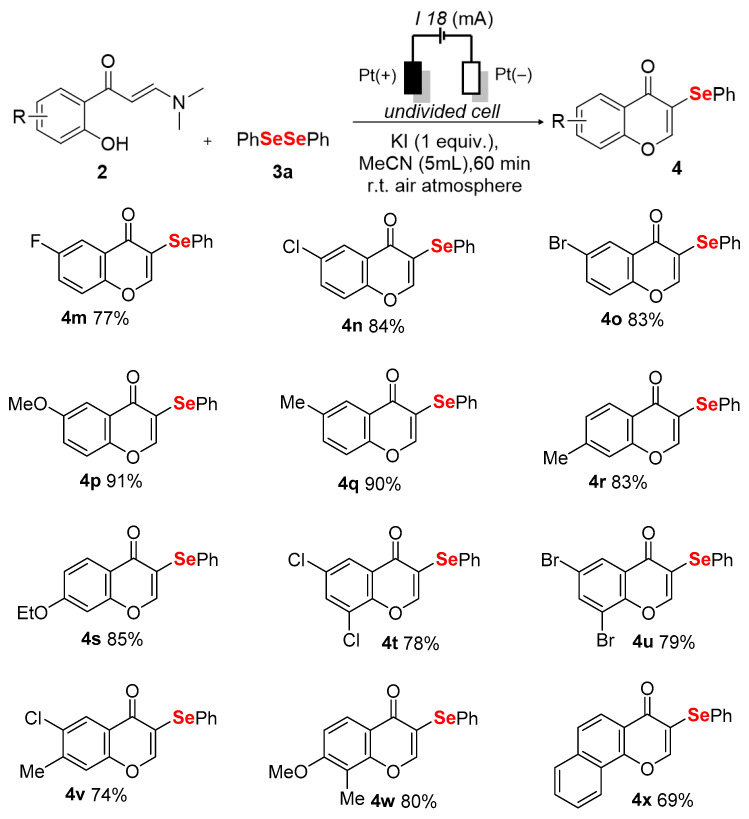
Scope of enaminones **1** ^[a],[b]^. Legend: ^[a]^ General reaction conditions: Pt plate electrodes (10 mm × 10 mm × 0.05 mm); (**1a**) (0.25 mmol), (**2a**) (0.25 mmol), KI (0.25 mmol), MeCN (5 mL), 60 min, r.t.; ^[b]^ isolated yields.

With the best result in hand (Table 1, entry 5), the generality and scope of this electrochemical domino C(sp^2^)–H bond selenylation and chromone annulation of the enaminones (**2**) with various diorganyl diselenides (**3**) were explored (Scheme 1 and Scheme 2).

Our initial evaluation of the method’s scope and efficiency tested a range of diorganyl diselenides (**3**) with a fixed enaminone substrate, **2a** (Scheme 1). The reaction proved effective for structurally diverse diselenides. Both electron-donating (e.g., -Me, -OMe) and electron-withdrawing (e.g., -F, -Cl, -CF_3_) aryl substituents yielded the target selenylated products **4a**–**f** in good to excellent yields (75–91%).

The reaction pathway displayed a notable electronic effect, with substrates bearing electron-donating groups generally providing higher yields (e.g., **4b**,**c**) than those with electron-withdrawing groups (**4d–f**). Steric hindrance from *ortho*-substituted aryl groups had a comparatively weaker impact, yielding results similar to their *para*-substituted analogs (**4b**–**c** vs. **4g**–**h**).

While aliphatic diselenides **3i**–**j** furnished products **4i**–**j** (71% and 45%, respectively), a benzylic substrate led to the formation of **4j**. A C-2 heteroaryl diselenide **3k** successfully generated the desired product **4k** in 62% yield. Finally, the method also accommodated a bulky-naphthyl group, affording product **4l** in 70% yield.

To further broaden the scope in relation to the substrate, we next investigated the influence of the enaminone **2**, using diphenyl diselenide **3a** as the constant reactant (Scheme 2). Enaminones bearing various aryl substituents, including halogens, ethers, and methyl groups, were evaluated.

The reaction tolerated a range of electronic effects on the enaminone’s aryl ring. Both electron-withdrawing and electron-donating groups served as viable substrates, delivering the corresponding selenylated products **4m**–**w** in good to excellent yields (74–91%). Notably, electron-donating groups at the C-5 position (**4p**–**q**) generally provided higher yields than electron-withdrawing groups at the same position (**4m**–**o**).

The methodology was also compatible with di-substituted enaminones, yielding selenylated chromones **4t**–**w** in good yields (74–80%). Finally, the system extended to a fused aromatic system, affording the naphthyl-derived product **4x** in 68% yield.

**Scheme 3:**
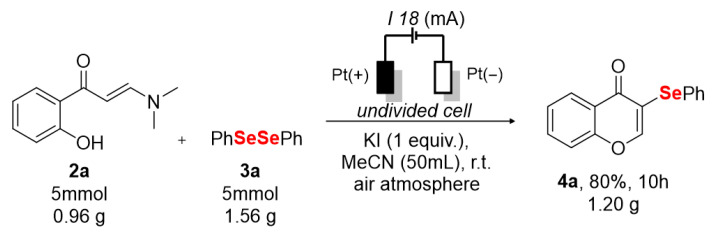
Gram-scale synthesis ^[a],[b]^. Legend: ^[a]^ General reaction conditions: Pt plate electrodes (10 mm × 10 mm × 0.05 mm); (**1a**) (5 mmol), (**2a**) (5 mmol), KI (5 mmol), MeCN (50 mL), 10 h, r.t.; ^[b]^ isolated yield.

To demonstrate the potential of this synthetic protocol, a gram-scale (5 mmol) electrochemical domino (sp^2^)–H bond selenylation and chromone annulation was performed using enaminone **2a** with diphenyl diselenide **3a**. This process afforded the selenylated product 4a in 80% yield (Scheme 3). Thus, the method offers a practical route for synthesizing biologically relevant hybrid compounds.

**Scheme 4:**
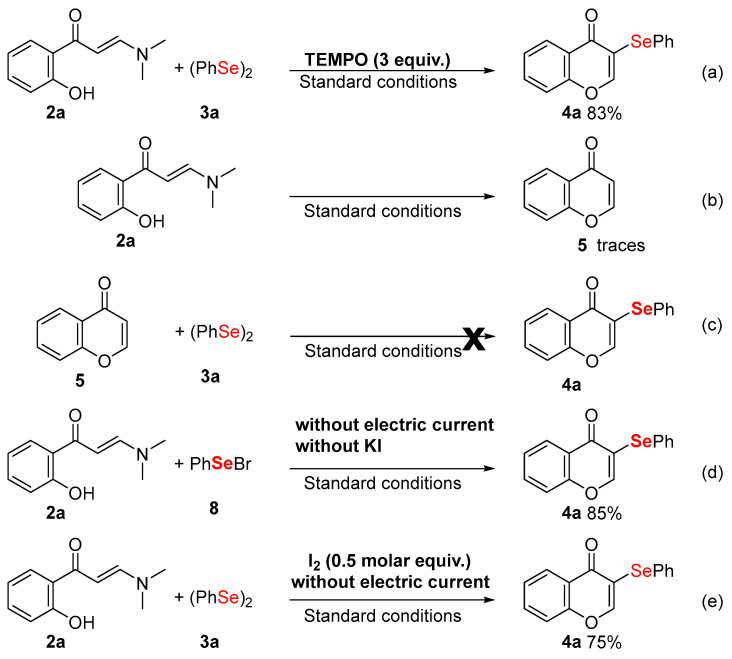
Control experiments.

For a better understanding of the reaction mechanism, a series of control experiments were performed; see Scheme 4. Under standard conditions, the addition of a stoichiometric amount of the radical scavenger TEMPO did not inhibit the transformation, yielding the selenylated chromone **4a** in 83% yield (Scheme 4, entry a). This outcome suggests that a radical pathway is unlikely. Omission of the diselenide **3a** resulted in only trace formation of chromone **5** (Scheme 4, entry b), indicating that this compound is probably not an intermediate in the process. Furthermore, subjecting chromone **5** to the optimized conditions did not yield the desired product **4a** (Scheme 4, entry c), which rules out the possibility of a mechanism involving initial chromone formation followed by selenylation. Finally, treatment of **2a** with PhSeBr **9** instead of diphenyl diselenide **3a** afforded product **4a** in 85% yield (Scheme 4, entry d). This finding supports the involvement of a phenylselenium cation species in the reaction pathway. Lastly, when a control experiment was performed using molecular iodine (0.5 molar equiv.) in place of KI and without applied electricity, the target selenylated product 4a was furnished in 75% yield (Scheme 4, entry e). This result suggests that the active iodine species in the standard reaction likely originates from the anodic oxidation of iodide.

**Scheme 5:**
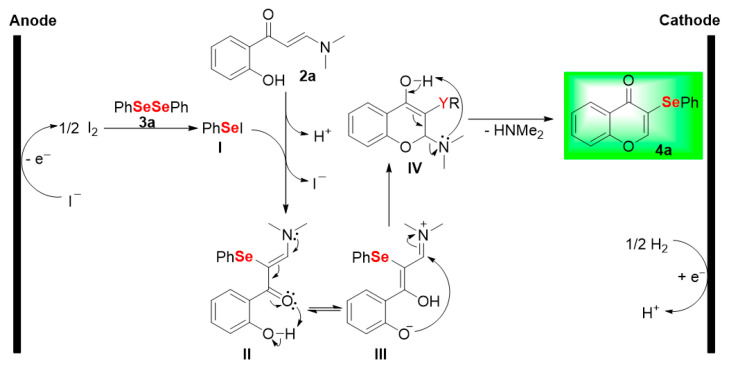
Proposed mechanism for the reaction.

On the basis of the control experiments and previously reported studies [85,86,87,88,89,90,91], a plausible mechanism for this electrochemical domino reaction is outlined in Scheme 5. The reaction is initiated at the anode where possible anodic oxidation of (I^−^) to molecular iodine (I_2_) occurs. Subsequently, I_2_ reacts with diphenyl diselenide **3a** to generate the electrophilic selenium species PhSeI **I**, which acts as the key selenylating agent [92,93,94]. The electrophilic selenium species **I** then reacts with enaminone **2a**, leading to the formation of intermediate **II** via electrophilic selenylation. Intermediate **II** undergoes tautomerization to generate intermediate **III**, which facilitates intramolecular cyclization to afford the cyclic intermediate **IV**. Finally, intermediate **IV** undergoes elimination of dimethylamine (HNMe_2_), delivering the desired selenylated chromone **4a**. Concurrently, at the cathode, protons are reduced to molecular hydrogen, completing the electrochemical cycle.

## 3. Materials and Methods

The reagents were acquired from commercial suppliers and utilized as received, without further purification. All solvents were of analytical (PA) grade and were also used without purification.

Proton nuclear magnetic resonance (^1^H NMR) spectra were acquired at 300 MHz using a Bruker DPX 300 NMR spectrometer (Bruker, Rheinstetten, Germany). Samples were prepared in CDCl_3_, and chemical shifts (δ) are reported in parts per million (ppm), referenced to the residual solvent peak of CDCl_3_ or to tetramethylsilane (TMS) as an external standard. Spectral data are presented as chemical shift, multiplicity, coupling constant (J) in Hz, and integrated intensity. Carbon-13 nuclear magnetic resonance (^13^C NMR) spectra were recorded at 50 MHz on the same instrument, using CDCl_3_ solutions. Chemical shifts are reported in ppm relative to the central solvent peak of CDCl_3_. The following abbreviations indicate signal multiplicity: s (singlet), d (doublet), t (triplet), q (quartet), quint (quintet), sext (sextet), and m (multiplet). All the NMR spectra were analyzed and processed with the MestReNova v12.0 software (Mestrelab Research S.L).

Melting points were measured using an SPLABOR SP-PFIII analog melting point apparatus with a heating plate (SPLABOR, Sao Paulo, Brazil). Purification by column chromatography was carried out with silica gel (230–400 mesh). Thin-layer chromatography (TLC) was performed using Merck silica gel GF254 of 0.25 mm thickness (Merck, Darmstadt, Germany). TLC visualization was achieved by examination under ultraviolet light or by staining with iodine vapor or an acidic vanillin solution.

### 3.1. General Procedure for Synthesis of 3-Selenyl-Chromones from the Cyclization of Enaminones Using Diorganyl Chalcogenides

An undivided three-necked flask (10 mL) equipped with a stirring bar was charged with appropriate enaminone **2** (0.25 mmol), diorganyl dichalcogenide **2** (0.25 mmol), diselenides (0.2 mmol), KI (1 equiv.) and MeCN (5 mL). The cell was equipped with platinum electrodes (1.0 × 1.0 × 0.05 mm) as the anode and cathode. The reaction mixture was stirred and electrolyzed at a constant current of 18 mA at ambient temperature and monitored by TLC. Upon completion, the solvent was removed under reduced pressure to afford the crude product, which was purified by flash column chromatography utilizing silica gel as stationary phase and eluate with a mixture of hexane/ethyl acetate to afford the desired product. Images of electrosynthetic setup are available in the Supplementary Materials (Figures SA and SB). Spectra of all the synthesized compounds are available in the Supplementary Materials (Figures S1–S48).

#### Spectral Data of Synthesized Products (**4a**–**x**)

3-(Phenylselanyl)-4*H*-chromen-4-one **4a**: Yield: 92% (69 mg); beige crystalline solid (crystallized from hexane/ethyl acetate); mp: 59–60 °C (59 °C) [22]; ^1^H NMR (300 MHz, Chloroform-*d*) δ 8.21 (d, *J* = 8.2 Hz, 1H), 7.87 (s, 1H), 7.71–7.53 (m, 3H), 7.45–7.33 (m, 2H), 7.32–7.21 (m, 3H). ^13^C NMR (75 MHz, Chloroform-*d*) δ 175.20, 156.34, 155.77, 133.83, 129.55, 128.12, 126.35, 125.56, 123.15, 118.06, 117.85; ^77^Se NMR (76.39 MHz, Chloroform-*d*) δ = 303.11.
3-((4-Methoxyphenyl)selanyl)-4*H*-chromen-4-one **4b**: Yield: 91% (75 mg); white crystalline solid (crystallized from hexane/ethyl acetate); mp: 91–92 °C (92–94 °C) [22]; ^1^H NMR (300 MHz, Chloroform-*d*) δ 8.18 (dd, *J* = 8.2, 1.7 Hz, 1H), 7.65–7.53 (m, 4H), 7.35 (ddd, *J* = 7.1, 3.5, 2.3 Hz, 2H), 6.88–6.77 (m, 2H), 3.76 (s, 3H). ^13^C NMR (75 MHz, Chloroform-*d*) δ 175.29, 160.22, 156.28, 153.85, 137.10, 133.69, 126.14, 125.38, 122.85, 119.35, 118.01, 117.02, 115.36, 55.31.
3-(*p*-Tolylselanyl)-4*H*-chromen-4-one **4c**: Yield: 84% (66 mg); beige solid (purified using hexane/ethyl acetate); mp: 90–91 °C (88–89 °C) [22]; ^1^H NMR (300 MHz, Chloroform-*d*) δ 8.19 (dd, *J* = 8.3, 1.8 Hz, 1H), 7.74 (s, 1H), 7.62 (ddd, *J* = 8.8, 7.2, 1.7 Hz, 1H), 7.54–7.46 (m, 2H), 7.43–7.32 (m, 2H), 7.09 (d, *J* = 7.8 Hz, 2H), 2.30 (s, 3H). ^13^C NMR (75 MHz, Chloroform-*d*) δ 175.23, 156.31, 154.80, 138.50, 134.63, 133.74, 130.43, 126.25, 125.44, 123.87, 123.01, 118.57, 118.03, 21.19.
3-((4-Chlorophenyl)selanyl)-4*H*-chromen-4-one **4d**: Yield: 86% (72 mg); beige crystalline solid (crystallized from hexane/ethyl acetate); mp: 116–118 °C (118–119 °C) [22]; ^1^H NMR (300 MHz, Chloroform-*d*) δ 8.20 (dd, *J* = 8.1, 1.4 Hz, 1H), 7.98 (s, 1H), 7.65 (ddd, *J* = 8.6, 7.0, 1.7 Hz, 1H), 7.54–7.44 (m, 2H), 7.46–7.34 (m, 2H), 7.27–7.16 (m, 2H). ^13^C NMR (75 MHz, Chloroform-*d*) δ 175.06, 156.50, 156.35, 134.81, 134.33, 133.99, 129.63, 126.68, 126.37, 125.72, 123.24, 118.11, 117.19.
3-((4-Fluorophenyl)selanyl)-4*H*-chromen-4-one **4e**: Yield: 83% (66 mg); yellow crystalline solid (crystallized from hexane/ethyl acetate); mp: 83–85 °C (84–85 °C) [22]; ^1^H NMR (300 MHz, Chloroform-*d*) δ 8.17 (dd, *J* = 8.3, 1.7 Hz, 1H), 7.84 (s, 1H), 7.66–7.53 (m, 3H), 7.36 (t, *J* = 7.3 Hz, 2H), 7.01–6.90 (m, 2H). ^13^C NMR (75 MHz, Chloroform-*d*) δ 175.11, 161.28, 156.29, 155.55, 136.45, 136.34, 133.91, 126.24, 125.61, 123.08, 122.55, 118.08, 117.98, 116.88, 116.59.
3-((3-(Trifluoromethyl)phenyl)selanyl)-4*H*-chromen-4-one **4f**: Yield: 75% (69 mg); beige crystalline sold (crystallized from hexane/ethyl acetate); mp: 109–112 °C (110–111 °C) [22]; ^1^H NMR (300 MHz, Chloroform-*d*) δ 8.17 (dd, *J* = 8.0, 1.7 Hz, 1H), 8.10 (s, 1H), 7.77 (s, 1H), 7.72–7.59 (m, 2H), 7.48–7.31 (m, 4H). ^13^C NMR (75 MHz, Chloroform-*d*) δ 174.90, 157.55, 156.34, 136.00, 134.11, 131.75 (q, *J*_C–F_ = 32.5 Hz), 130.18, 129.71, 129.26 (q, *J*_C–F_ = 3.3 Hz), 129.21, 129.16, 129.11, 126.36 (q, *J*_C–F_ = 271 Hz), 125.83, 124.58 (q, *J*_C–F_ = 3.5 Hz), 124.53, 124.48, 123.31, 118.16, 116.20.
3-((2-Methoxyphenyl)selanyl)-4*H*-chromen-4-one **4g**: Yield: 88% (73 mg); white solid (purified using hexane/ethyl acetate); mp: 110–111 °C (110–111 °C) [22]; ^1^H NMR (300 MHz, Chloroform-*d*) δ 8.21 (dd, *J* = 8.0, 1.7 Hz, 1H), 8.02 (s, 1H), 7.64 (ddd, *J* = 8.6, 7.1, 1.7 Hz, 1H), 7.46–7.34 (m, 2H), 7.23–7.11 (m, 2H), 6.80 (qd, *J* = 7.9, 7.5, 1.2 Hz, 2H), 3.84 (s, 3H). ^13^C NMR (75 MHz, Chloroform-*d*) δ 175.38, 157.72, 157.42, 156.42, 133.88, 131.70, 128.62, 126.40, 125.63, 123.30, 121.74, 118.40, 118.13, 114.79, 110.76, 55.93.
3-(*o*-Tolylselanyl)-4*H*-chromen-4-one **4h**: Yield: 86% (68 mg); red crystalline solid (crystallized from hexane/ethyl acetate); mp: 117–118 °C (118 °C) [22]; ^1^H NMR (300 MHz, Chloroform-*d*) δ 8.22 (dd, *J* = 8.2, 1.8 Hz, 1H), 7.64 (q, *J* = 7.2, 6.3 Hz, 2H), 7.42 (dt, *J* = 14.6, 7.3 Hz, 3H), 7.28–7.12 (m, 2H), 7.07 (t, *J* = 6.5 Hz, 1H), 2.47 (s, 3H). ^13^C NMR (75 MHz, Chloroform-*d*) δ 175.33, 156.37, 154.76, 140.57, 134.43, 133.82, 130.50, 128.56, 128.47, 127.05, 126.26, 125.53, 122.95, 118.07, 117.26, 22.34.
3-(Butylselanyl)-4*H*-chromen-4-one **4i**: Yield: 71% (50 mg); yellow solid (purified using hexane/ethyl acetate); mp: 55–56 °C (54–55 °C) [22]; ^1^H NMR (300 MHz, Chloroform-*d*) δ 8.24–8.17 (m, 1H), 8.14 (s, 1H), 7.64 (ddd, *J* = 8.6, 5.6, 1.6 Hz, 1H), 7.52–7.33 (m, 2H), 2.95–2.77 (m, 2H), 1.60 (q, *J* = 7.6 Hz, 2H), 1.47–1.29 (m, 2H), 0.86 (t, *J* = 7.3 Hz, 3H). ^13^C NMR (75 MHz, Chloroform-*d*) δ 175.89, 156.35, 156.24, 133.69, 126.29, 125.47, 123.19, 118.03, 114.74, 32.13, 25.97, 22.77, 13.54.
3-(Benzylselanyl)-4*H*-chromen-4-one **4j**: Yield: 45% (36 mg); white solid (purified using hexane/ethyl acetate); mp: 94–96 °C (95–96 °C) [22]; ^1^H NMR (300 MHz, Chloroform-*d*) δ 8.27–8.17 (m, 1H), 7.85 (s, 1H), 7.64 (ddd, *J* = 8.6, 7.2, 1.7 Hz, 1H), 7.46–7.33 (m, 3H), 7.16 (q, *J* = 4.6 Hz, 4H), 4.08 (s, 2H). ^13^C NMR (75 MHz, Chloroform-*d*) δ 175.83, 157.59, 156.30, 138.46, 134.12, 133.80, 128.98, 128.42, 126.89, 126.32, 125.61, 123.31, 118.09, 29.77.
3-(Thiophen-2-ylselanyl)-4*H*-chromen-4-one **4k**: Yield: 62% (48 mg); yellow solid (purified using hexane/ethyl acetate); mp: 113–115 °C (114–116 °C) [22]; ^1^H NMR (300 MHz, Chloroform-*d*) δ 8.15 (dd, *J* = 8.2, 1.7 Hz, 1H), 7.65–7.57 (m, 1H), 7.52 (s, 1H), 7.49–7.42 (m, 1H), 7.34 (dt, *J* = 8.0, 2.9 Hz, 3H), 7.01 (dd, *J* = 5.4, 3.5 Hz, 1H). ^13^C NMR (75 MHz, Chloroform-*d*) δ 175.01, 156.25, 153.25, 137.99, 133.84, 132.82, 128.63, 125.99, 125.48, 122.66, 119.90, 119.86, 118.06.
3-(Naphthalen-1-ylselanyl)-4*H*-chromen-4-one **4l**: Yield: 88% (77 mg); yellow solid (purified using hexane/ethyl acetate); mp: 68–70 °C (67–68 °C) [22]; ^1^H NMR (300 MHz, Chloroform-*d*) δ 8.21 (dd, *J* = 8.2, 1.7 Hz, 1H), 8.09 (s, 1H), 7.85 (s, 1H), 7.74 (dd, *J* = 9.7, 6.0 Hz, 3H), 7.66–7.57 (m, 2H), 7.44 (dd, *J* = 6.2, 3.3 Hz, 2H), 7.41–7.29 (m, 2H). ^13^C NMR (75 MHz, Chloroform-*d*) δ 175.23, 156.32, 155.70, 133.98, 133.86, 133.31, 132.73, 130.75, 129.11, 127.80, 127.53, 126.63, 126.60, 126.30, 125.58, 125.39, 123.10, 118.09, 117.93.
6-Fluoro-3-(phenylselanyl)-4*H*-chromen-4-one **4m**: Yield: 77% (62 mg); white solid (purified using hexane/ethyl acetate); mp: 118–120 °C (120–121 °C) [24]; ^1^H NMR (300 MHz, Chloroform-*d*) δ 7.86–7.76 (m, 2H), 7.56 (dd, *J* = 6.6, 3.0 Hz, 2H), 7.38 (ddt, *J* = 10.3, 7.3, 3.6 Hz, 2H), 7.27 (hept, *J* = 4.4, 3.8 Hz, 3H). ^13^C NMR (75 MHz, Chloroform-*d*) δ 174.49 (d, *J*_C–F_ = 2.4 Hz), 159.62 (d, *J*_C–F_ = 247.7 Hz), 155.68, 152.57 (d, *J*_C–F_ = 1.7 Hz), 134.01, 129.61, 128.31, 127.74, 124.14 (d, *J*_C–F_ = 7.3 Hz), 122.16 (d, *J*_C–F_ = 25.7 Hz), 120.31 (d, *J*_C–Fk_ = 8.1 Hz), 117.35, 111.00 (d, *J*_C–F_ = 23.9 Hz).
6-Chloro-3-(phenylselanyl)-4*H*-chromen-4-one **4n**: Yield: 84% (71 mg); yellow crystalline solid (crystallized from hexane/ethyl acetate); mp:104–106 °C (105–106 °C) [22]; ^1^H NMR (300 MHz, Chloroform-*d*) δ 8.10 (d, *J* = 2.6 Hz, 1H), 7.78 (s, 1H), 7.59–7.48 (m, 3H), 7.37–7.23 (m, 4H). ^13^C NMR (75 MHz, Chloroform-*d*) δ 174.02, 155.43, 154.59, 134.12, 134.05, 131.39, 129.64, 128.39, 127.57, 125.51, 123.82, 119.90, 118.14.
6-Bromo-3-(phenylselanyl)-4*H*-chromen-4-one **4o**: Yield: 83% (79 mg); beige crystalline solid (crystallized from hexane/ethyl acetate); mp: 105 °C (104–105 °C) [22]; ^1^H NMR (300 MHz, Chloroform-*d*) δ 8.25 (d, *J* = 2.5 Hz, 1H), 7.77 (s, 1H), 7.65 (dd, *J* = 8.9, 2.6 Hz, 1H), 7.55 (dd, *J* = 6.8, 3.0 Hz, 2H), 7.31–7.21 (m, 4H). ^13^C NMR (75 MHz, Chloroform-*d*) δ 173.86, 155.41, 155.01, 136.79, 134.14, 129.64, 128.71, 128.40, 127.56, 124.17, 120.12, 118.88, 118.23.
6-Methoxy-3-(phenylselanyl)-4*H*-chromen-4-one **4p**: Yield: 91% (75 mg); white crystalline solid (crystallized from hexane/ethyl acetate); mp: 97–99 °C (99–101 °C) [22]; ^1^H NMR (300 MHz, Chloroform-*d*) δ 7.86 (s, 1H), 7.54 (ddd, *J* = 9.2, 4.1, 2.4 Hz, 3H), 7.36–7.17 (m, 5H), 3.82 (s, 3H). ^13^C NMR (75 MHz, Chloroform-*d*) δ 175.00, 157.15, 155.78, 151.18, 133.63, 129.49, 128.39, 128.00, 123.94, 123.76, 119.50, 116.73, 105.24, 55.91.
6-Methyl-3-(phenylselanyl)-4*H*-chromen-4-one **4q**: Yield: 90% (71 mg); beige crystalline solid (crystallized from hexane/ethyl acetate); mp: 99–101 °C (100–101 °C) [22]; ^1^H NMR (300 MHz, Chloroform-*d*) δ 7.91 (s, 1H), 7.79 (d, *J* = 1.2 Hz, 1H), 7.58–7.46 (m, 2H), 7.37 (dd, *J* = 8.6, 2.2 Hz, 1H), 7.21 (dt, *J* = 5.6, 3.8 Hz, 4H), 2.35 (s, 3H). ^13^C NMR (75 MHz, Chloroform-*d*) δ 175.13, 155.85, 154.54, 135.55, 135.08, 133.61, 129.49, 128.35, 127.99, 125.46, 122.75, 117.83, 117.35, 20.95.
7-Methyl-3-(phenylselanyl)-4*H*-chromen-4-one **4r**: Yield: 83% (66 mg); white solid (purified using hexane/ethyl acetate); mp: 109–110 °C (110–112 °C) [22]; ^1^H NMR (300 MHz, Chloroform-*d*) δ 8.04 (d, *J* = 8.1 Hz, 1H), 7.79 (s, 1H), 7.61–7.48 (m, 2H), 7.31–7.20 (m, 3H), 7.15 (d, *J* = 8.0 Hz, 2H), 2.40 (s, 3H). ^13^C NMR (75 MHz, Chloroform-*d*) δ 174.98, 156.41, 155.58, 145.27, 133.69, 129.50, 128.30, 128.02, 127.06, 125.99, 120.88, 117.74, 117.59, 21.83.
7-Ethoxy-3-(phenylselanyl)-4*H*-chromen-4-one **4s**: Yield: 85% (73 mg); yellow solid (purified using hexane/ethyl acetate); mp: 92–93 °C (90–92 °C) [22]; ^1^H NMR (300 MHz, Chloroform-*d*) δ 8.03 (d, *J* = 8.9 Hz, 1H), 7.72 (s, 1H), 7.59–7.42 (m, 2H), 7.22 (q, *J* = 3.8 Hz, 3H), 6.87 (dd, *J* = 9.0, 2.4 Hz, 1H), 6.68 (s, 1H), 4.01 (q, *J* = 6.9 Hz, 2H), 1.39 (t, *J* = 7.1 Hz, 3H). ^13^C NMR (75 MHz, Chloroform-*d*) δ 174.42, 163.51, 158.07, 155.30, 133.64, 129.48, 128.34, 127.98, 127.53, 117.61, 116.81, 115.21, 100.57, 64.32, 14.53.
6,8-Dichloro-3-(phenylselanyl)-4*H*-chromen-4-one **4t**: Yield: 78% (72 mg); white crystalline solid (crystallized from hexane/ethyl acetate); mp: 112–113 °C (113–115 °C) [22]; ^1^H NMR (300 MHz, Chloroform-*d*) δ 8.03 (d, *J* = 2.5 Hz, 1H), 7.73 (s, 1H), 7.68–7.55 (m, 3H), 7.30 (dd, *J* = 5.2, 2.0 Hz, 3H). ^13^C NMR (75 MHz, Chloroform-*d*) δ 173.44, 154.20, 150.64, 134.81, 133.89, 131.08, 129.80, 128.78, 126.67, 124.41, 124.28, 119.34.
6,8-Dibromo-3-(phenylselanyl)-4*H*-chromen-4-one **4u**: Yield: 79% (91 mg); beige crystalline solid (crystallized from hexane/ethyl acetate); mp: 114–116 °C (116–118 °C) [22]; ^1^H NMR (300 MHz, Chloroform-*d*) δ 8.24 (d, *J* = 2.3 Hz, 1H), 7.94 (d, *J* = 2.3 Hz, 1H), 7.73 (s, 1H), 7.64–7.54 (m, 2H), 7.31 (dd, *J* = 5.2, 2.1 Hz, 3H). ^13^C NMR (75 MHz, Chloroform-*d*) δ 173.37, 154.27, 151.95, 139.46, 134.85, 129.81, 128.80, 128.20, 126.64, 124.66, 119.34, 118.70, 112.81.
6-Chloro-7-methyl-3-(phenylselanyl)-4*H*-chromen **4v**: Yield: 74% (62 mg); white crystalline solid (crystallized from hexane/ethyl acetate); mp: 137–138 °C (138–140 °C) [22]; ^1^H NMR (300 MHz, Chloroform-*d*) δ 8.09 (s, 1H), 7.76 (s, 1H), 7.55 (dd, *J* = 6.6, 3.0 Hz, 2H), 7.33–7.21 (m, 4H), 2.42 (s, 3H). ^13^C NMR (75 MHz, Chloroform-*d*) δ 174.00, 155.42, 154.55, 143.21, 133.97, 132.17, 129.58, 128.26, 127.83, 125.75, 122.01, 119.86, 117.81, 20.81.
7-Methoxy-8-methyl-3-(phenylselanyl)-4*H*-chromen-4-one **4w**: Yield: 80% (69 mg); yellow crystalline solid (crystallized from hexane/ethyl acetate); mp: 130–131 °C (128–130 °C) [22]; ^1^H NMR (300 MHz, Chloroform-*d*) δ 8.09 (s, 1H), 7.76 (s, 1H), 61–7.49 (m, 2H), 7.32–7.18 (m, 4H), 2.42 (s, 3H). ^13^C NMR (75 MHz, Chloroform-*d*) δ 175.11, 161.39, 155.78, 155.46, 133.57, 129.46, 128.53, 127.91, 124.81, 117.06, 116.84, 113.96, 108.93, 56.10, 8.05.
3-(Phenylselanyl)-4*H*-benzo[*h*]chromen-4-one **4x**: Yield: 69% (61 mg); brown solid (purified using hexane/ethyl acetate); mp: 110–111 °C (111–113 °C) [22]; ^1^H NMR (300 MHz, Chloroform-*d*) δ 8.38 (d, *J* = 7.6 Hz, 1H), 8.21–8.07 (m, 2H), 7.94–7.84 (m, 1H), 7.77–7.58 (m, 3H), 7.49–7.39 (m, 2H), 7.34–7.17 (m, 3H). ^13^C NMR (75 MHz, Chloroform-*d*) δ 174.80, 155.51, 153.79, 135.82, 133.43, 130.54, 129.56, 129.32, 128.13, 127.44, 127.36, 125.84, 123.78, 122.16, 121.04, 119.78.

## 4. Conclusions

In conclusion, we have developed an efficient and alternative sustainable electrochemical protocol for the domino (sp^2^)–H bond selenylation and chromone annulation of 2-hydroxyaryl enaminones via deamination using diorganyl diselenides. This transformation proceeds under mild and greener conditions, employing KI as a low-cost electrolyte in acetonitrile under open-air atmosphere, and is completed within 60 min. Under the optimized conditions, a wide range of selenylated chromones were obtained in good to excellent yields with high regioselectivity. The methodology exhibits broad substrate scope and excellent functional-group tolerance, being effective for both enaminones and structurally diverse diselenides. Notably, the successful gram-scale synthesis highlights the practicality and potential industrial applicability of this electrosynthetic strategy.

Overall, the key advantages of this benign and robust protocol include (i) compatibility with a wide range of 2-hydroxyaryl enaminones and diselenides; (ii) use of an inexpensive, readily available and bench-stable electrolyte; (iii) operation under ambient air without external oxidants or catalysts; (iv) short reaction time at room temperature; (v) scalability; and (vi) the use of non-toxic, easy-to-handle reagents. These features collectively position this method as a valuable contribution to green electrosynthesis and the sustainable construction of selenium-containing chromone hybrid molecules.

## Figures and Tables

**Table 1:** Optimization of reaction conditions ^[a]^.

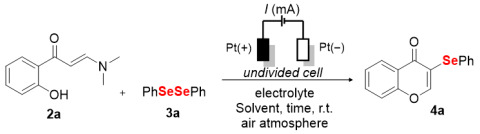					
Entry	Solvent (mL)	Electrolyte (Equiv.)	Current (mA)	Time (min)	Yield (%) ^[b]^
1	MeCN (5 mL)	TBAI (1)	18 mA	60	58
2	MeCN (5 mL)	TBAB (1)	18 mA	60	21
3	MeCN (5 mL)	TBACl (1)	18 mA	60	39
4	MeCN (5 mL)	TBAAc (1)	18 mA	60	23
**5**	**MeCN (5 mL)**	**KI (1)**	**18 mA**	**60**	**92**
6	MeCN (5 mL)	KBr (1)	18 mA	60	traces
7	MeCN (5 mL)	KCl (1)	18 mA	60	N.R.
8	MeCN (5 mL)	KI (1.5)	18 mA	60	80
9	MeCN (5 mL)	KI (0.5)	18 mA	60	35
10	DMSO (5 mL)	KI (1)	18 mA	60	N.R.
11	MeOH (5 mL)	KI (1)	18 mA	60	69
12	THF (5 mL)	KI (1)	18 mA	60	N.R.
13	DCM (5 mL)	KI (1)	18 mA	60	N.R.
14	50% MeCN:H_2_O (5 mL)	KI (1)	18 mA	60	28
15	MeCN (3 mL)	KI (1)	18 mA	60	74
16	MeCN (10 mL)	KI (1)	18 mA	60	91
17	MeCN (5 mL)	KI (1)	25 mA	60	70
18	MeCN (5 mL)	KI (1)	10 mA	60	52
19	MeCN (5 mL)	KI (1)	18 mA	30	62
20	MeCN (5 mL)	KI (1)	18 mA	90	79
21 ^[c]^	MeCN (5 mL)	KI (1)	18 mA	60	81
22 ^[d]^	MeCN (5 mL)	KI (1)	18 mA	60	76
23 ^[e]^	MeCN (5 mL)	KI (1)	18 mA	60	N.R.

^[a]^ General reaction conditions: Pt plate electrodes (10 mm × 10 mm × 0.05 mm); (**1a**) (0.25 mmol), (**2a**) (0.25 mmol); ^[b]^ isolated yields; ^[c]^ Pt anode and graphite rod (F 4 mm) cathode; ^[d]^ graphite rod (F 4 mm) anode and Pt cathode; ^[e]^ graphite rod (F 4 mm) anode and graphite rod (F 4 mm) cathode; N.R. = for all cases, starting material was recovered.

## Data Availability

The data that support the findings of this study are available in the Supplementary Material of this article.

## Supplementary Materials

The following supporting information can be downloaded at https://www.mdpi.com/article/10.3390/molecules31020391/s1, Electrosynthetic setup (Figures SA and SB) NMR Spectra (Figures S1–S48).
