# Supplementary material for: Electrochemical Synthesis of 3-Selenyl-Chromones via Domino C(sp2)-H Bond Selenylation/Annulation of Enaminones
Source: Molecules. 2026 Jan 22;31(2):391. doi: 10.3390/molecules31020391 (PMC12844472; doi:10.3390/molecules31020391)
Supplement: Supplementary file 1 [file molecules-31-00391-s001.zip › molecules-4103182-supplementary.pdf]

Electronic Supplementary Information

Electrochemical Synthesis of 3-Selenyl-Chromones via Domino $C(sp^2)$-H Bond Selenylation/Annulation of Enaminones

João M. Brito [1], Isabella M. e Oliveira [1], Cassio A. O. Moraes [2], Alex R. Schneider [3], Tiago E. A. Frizon [3], Giancarlo V. Botteselle [4], Vijay P. Singh [5], André L. Stein [6], Gleison A. Casagrande [2], Giuseppe A. Camara [2], Antonio L. Braga [3], Jamal Rafique [1,2,*] and Sumbal Saba [1,*]

[1] Laboratório de Síntese Sustentável e Organocalcogênio (LABSO), Instituto de Química (IQ), Universidade Federal de Goiás–UFG, Goiania 74690-900, Brazil;

[2] Instituto de Química (INQUI), Universidade Federal do Mato Grosso do Sul–UFMS, Campo Grande 79074-460, Brazil;

[3] Departamento de Química, Universidade Federal de Santa Catarina–UFSC, Florianopolis 88040-900, Brazil;

[4] Departamento de Química, Universidade Estadual do Centro-Oeste–UNICENTRO, Guarapuava 85819-110, Brazil;

[5] Department of Chemistry & Centre of Advanced Studies in Chemistry, Panjab University, Sector-14, Chandigarh 160 014, India;

[6] Laboratório de Pesquisa de Produtos Naturais, Instituto de Química, Universidade Federal de Mato Grosso–UFMT, Cuiaba 78060-900, Brazil;

* Corresponding author:
jamal.rafique@ufms.br , jamal.chm@gmail.com (J.R.). sumbalsaba@ufg.br (S.S.)

Contents

Figure SA: Electrosynthetic setup for the synthesis of 3-selenyl-chromones……………......S2

Figure SB: Platinum work electrodes..S2

Copies of (^{1}H and ^{13}C) NMR spectra...S3-27

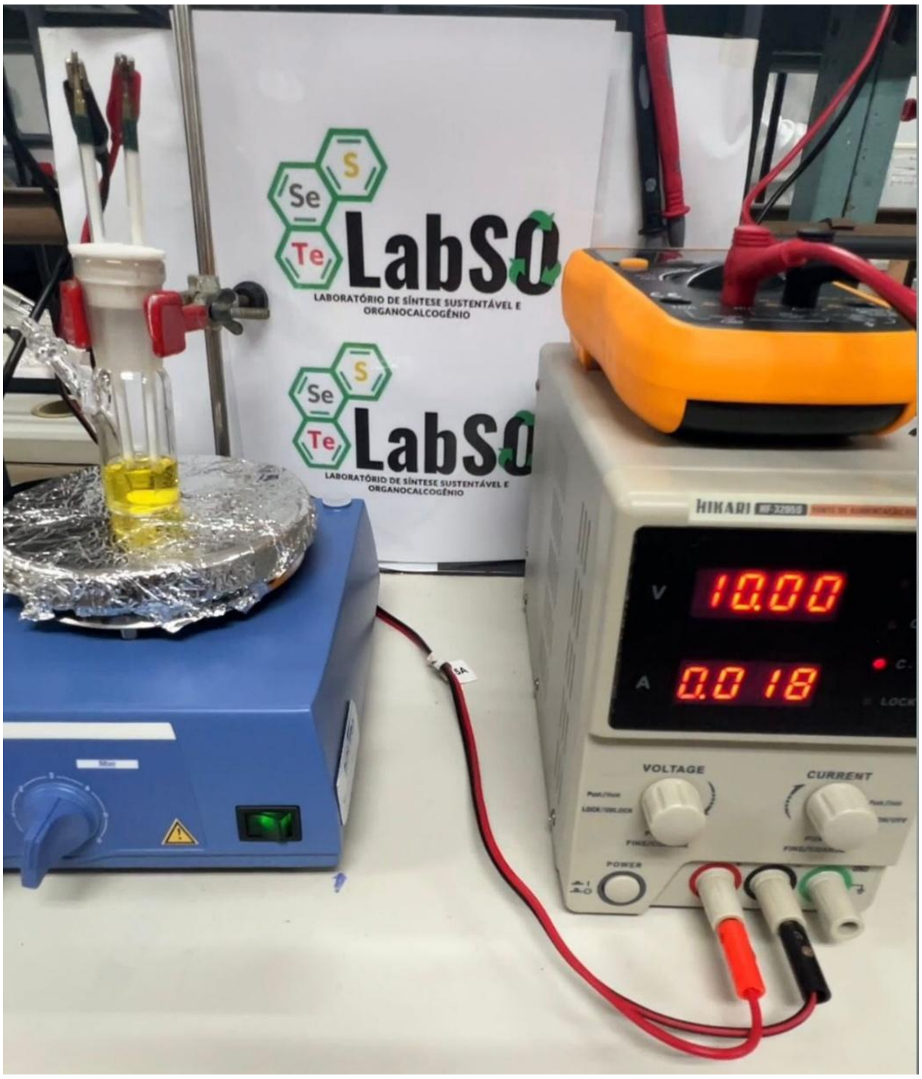

Figure SA: Electrosynthetic setup for the synthesis of 3-selenyl-chromones.

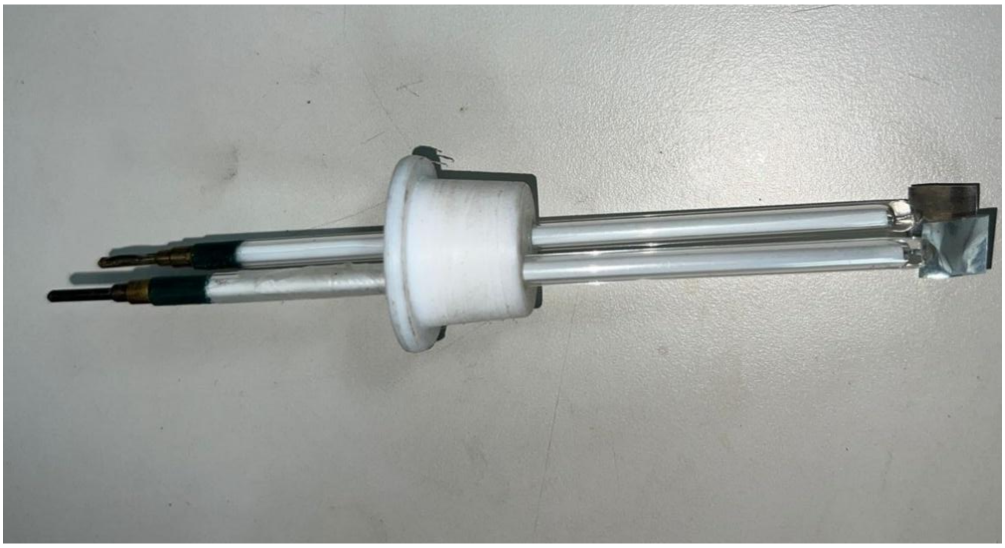

Figure SB: Platinum work electrodes.

Copies of (^{1}H and ^{13}C) NMR spectra

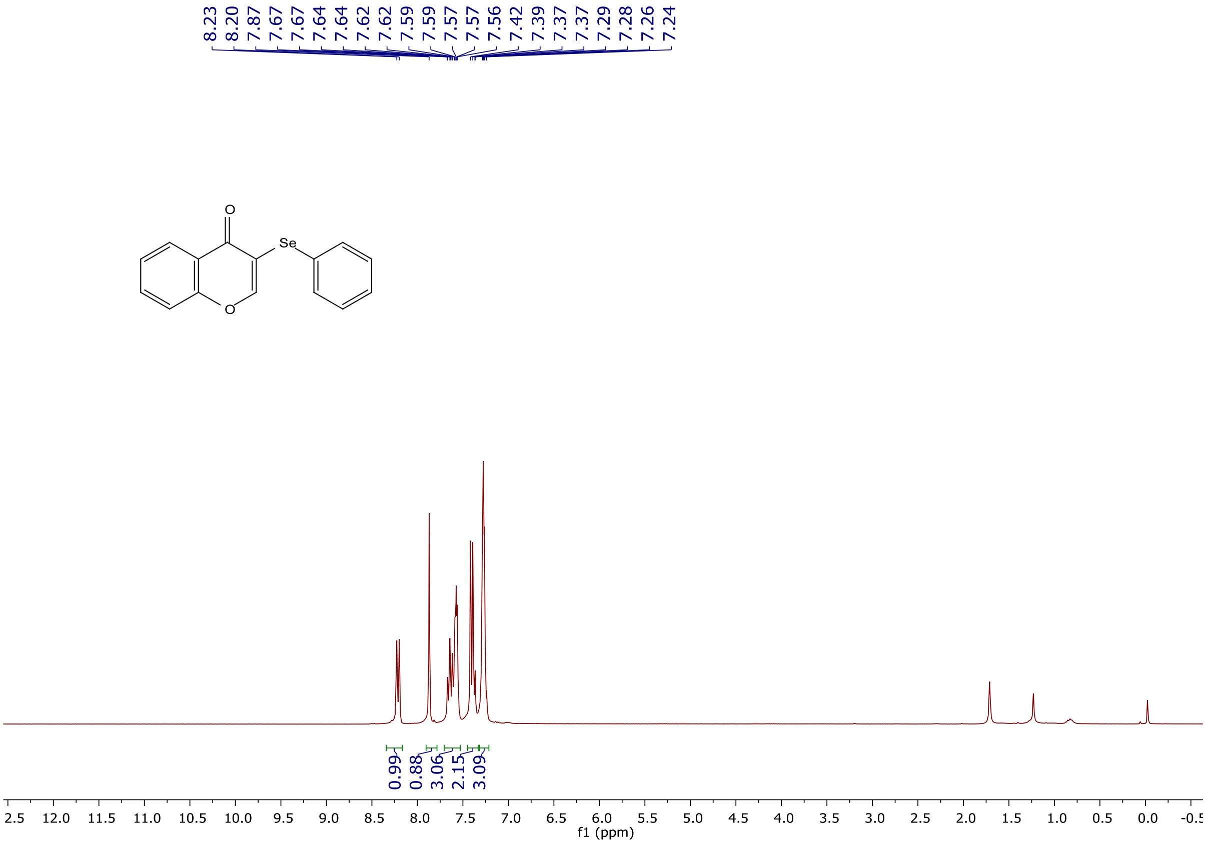

Figure S1. ^{1}H-NMR Spectra of the compound **4a,** in $CDCl_3$, 300 MHz

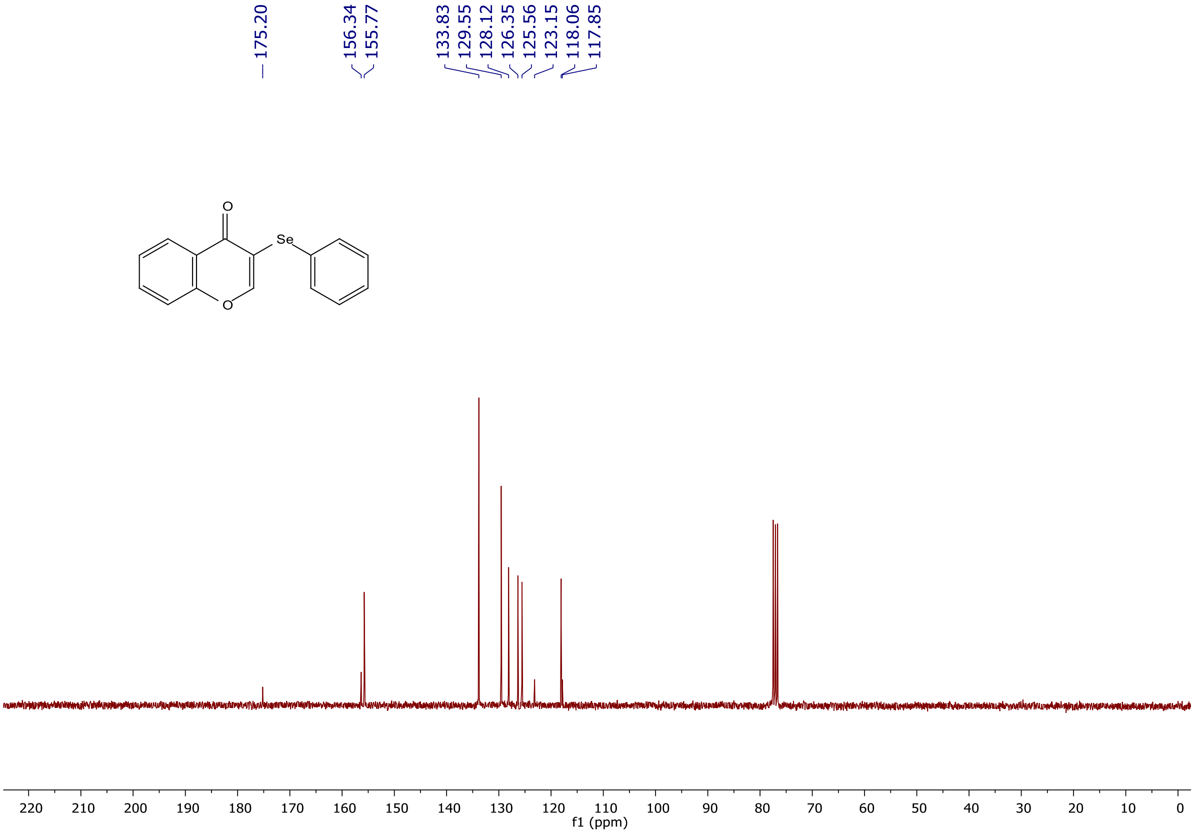

Figure S2. ^{13}C-NMR Spectra of the compound **4a**, in $CDCl_3$, 75 MHz

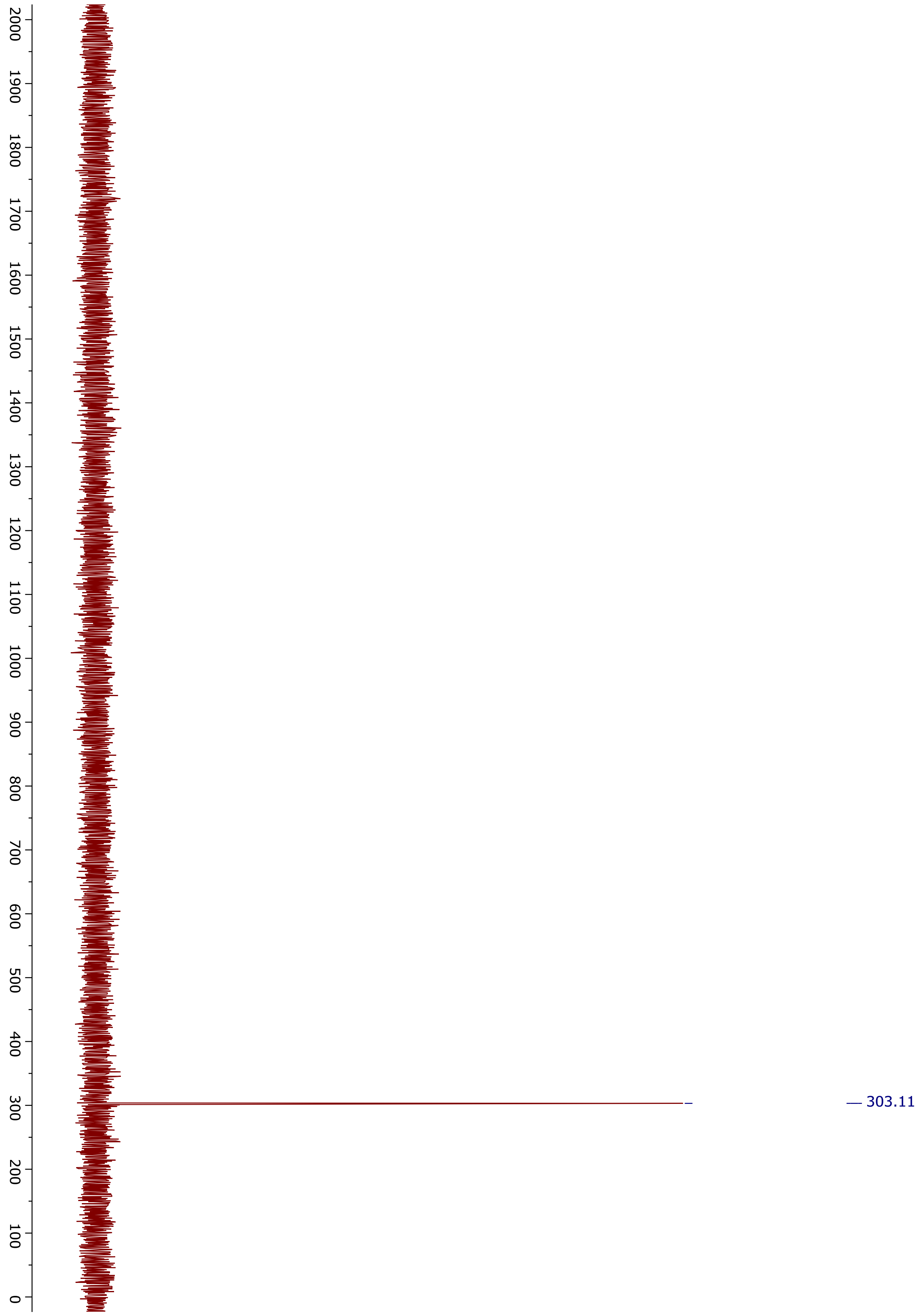

Figure S1A. ^{77}Se-NMR Spectra of the compound **4a,** in $CDCl_3$, 76.39 MHz

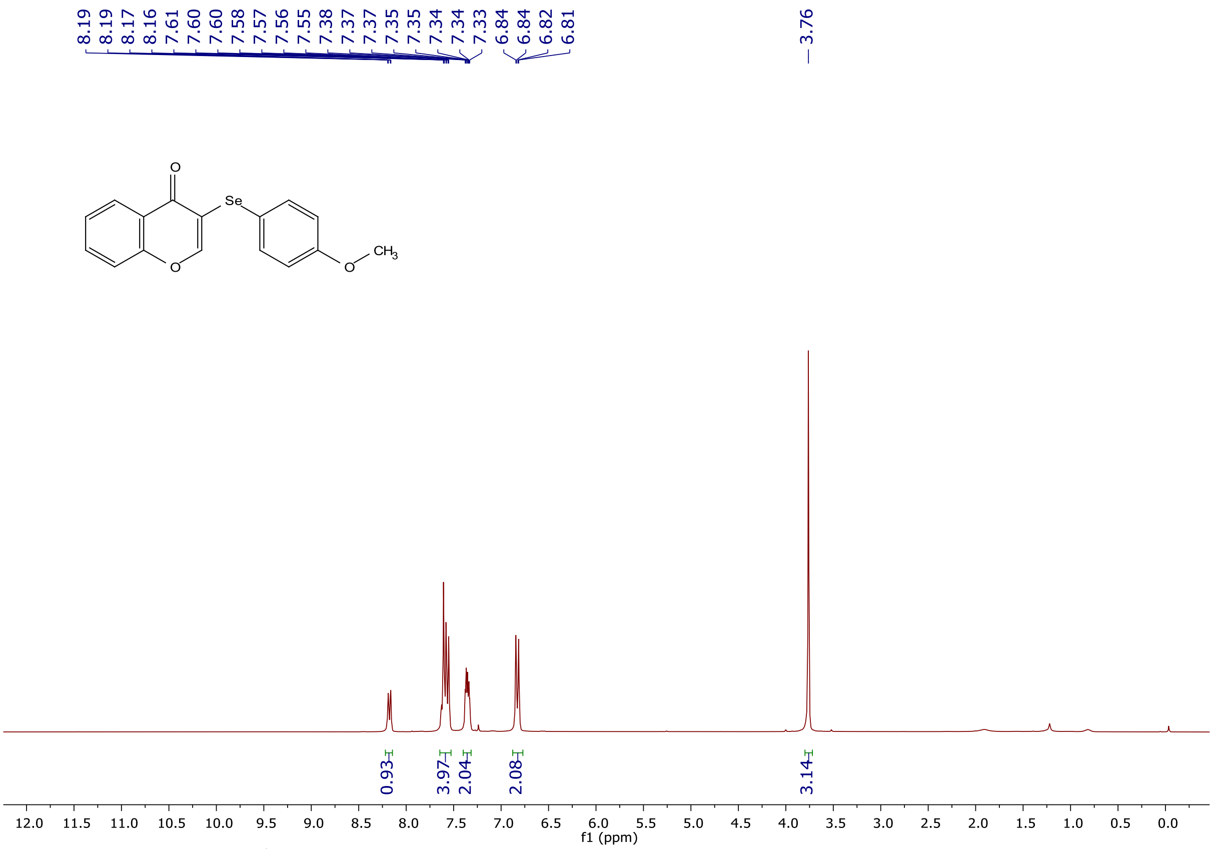

Figure S3. ^{1}H-NMR Spectra of the compound **4b,** in $CDCl_3$, 300 MHz

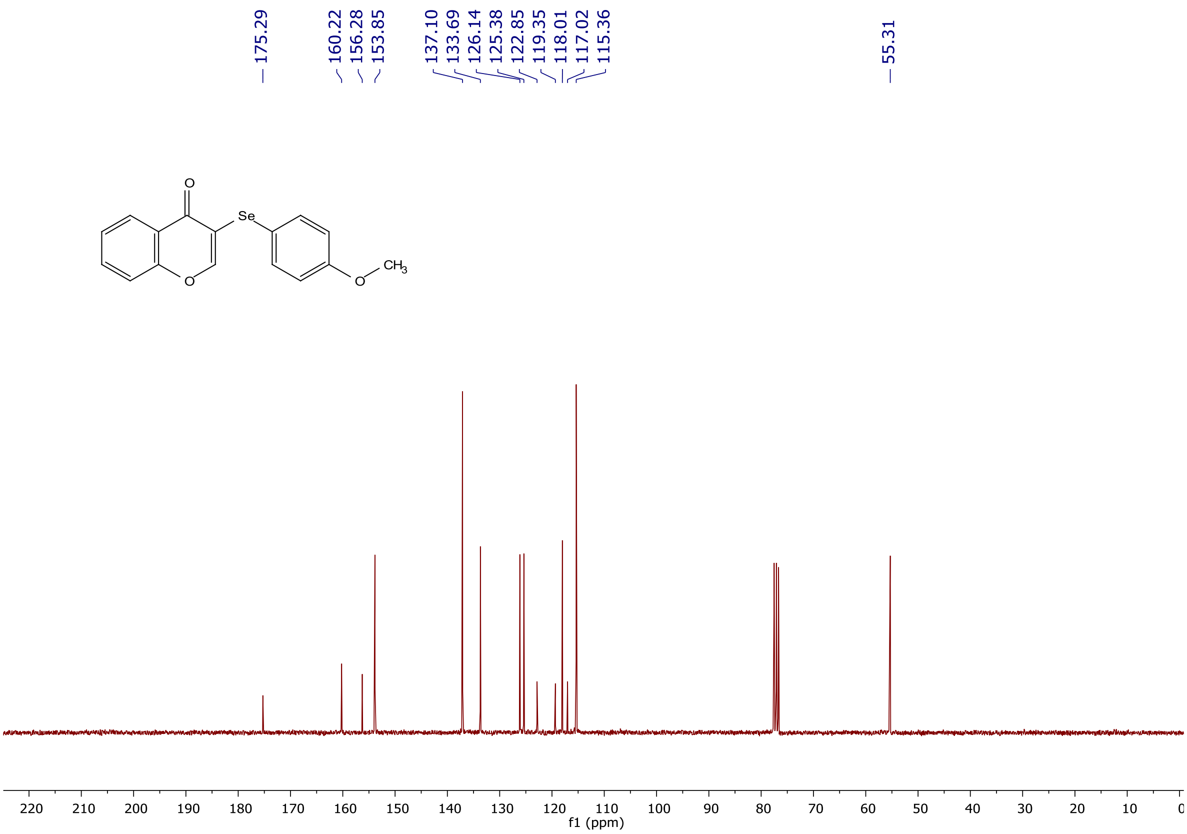

Figure S4. ^{13}C-NMR Spectra of the compound **4b**, in $CDCl_3$, 75 MHz

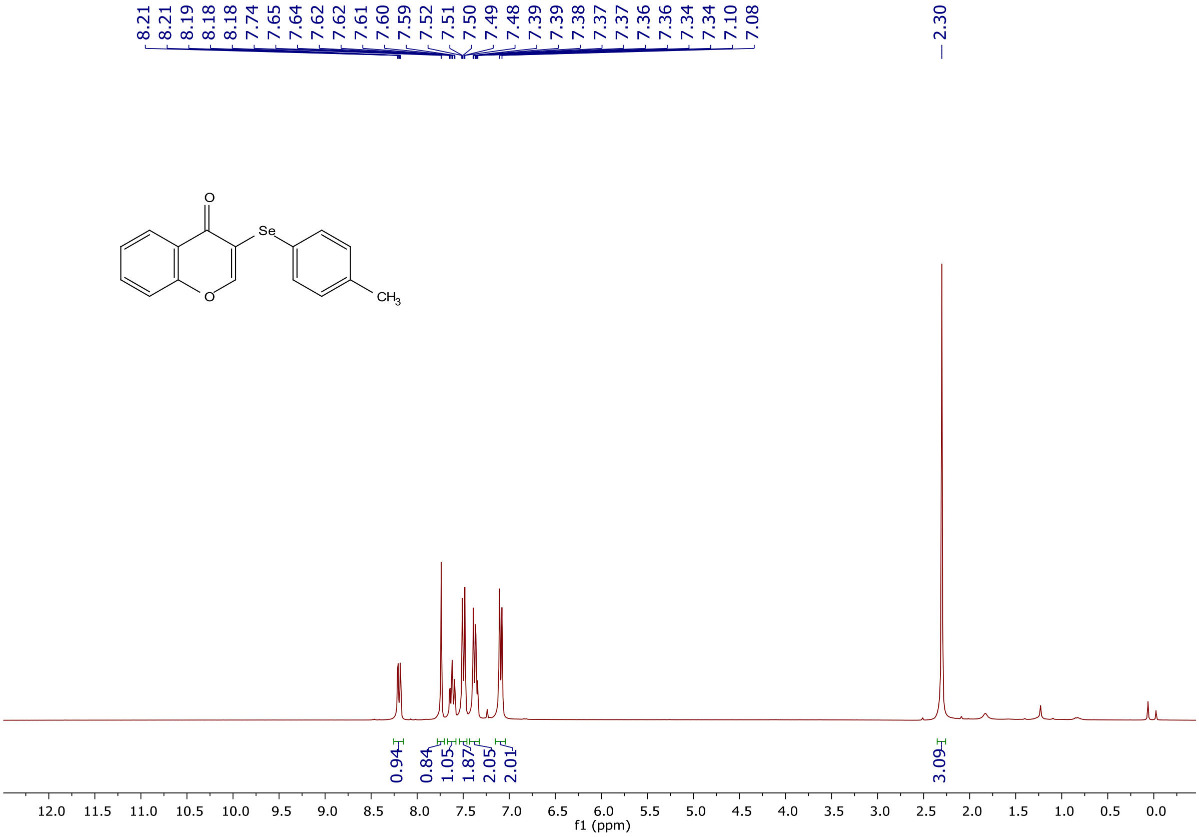

Figure S5. ^{1}H-NMR Spectra of the compound **4c,** in $CDCl_3$, 300 MHz

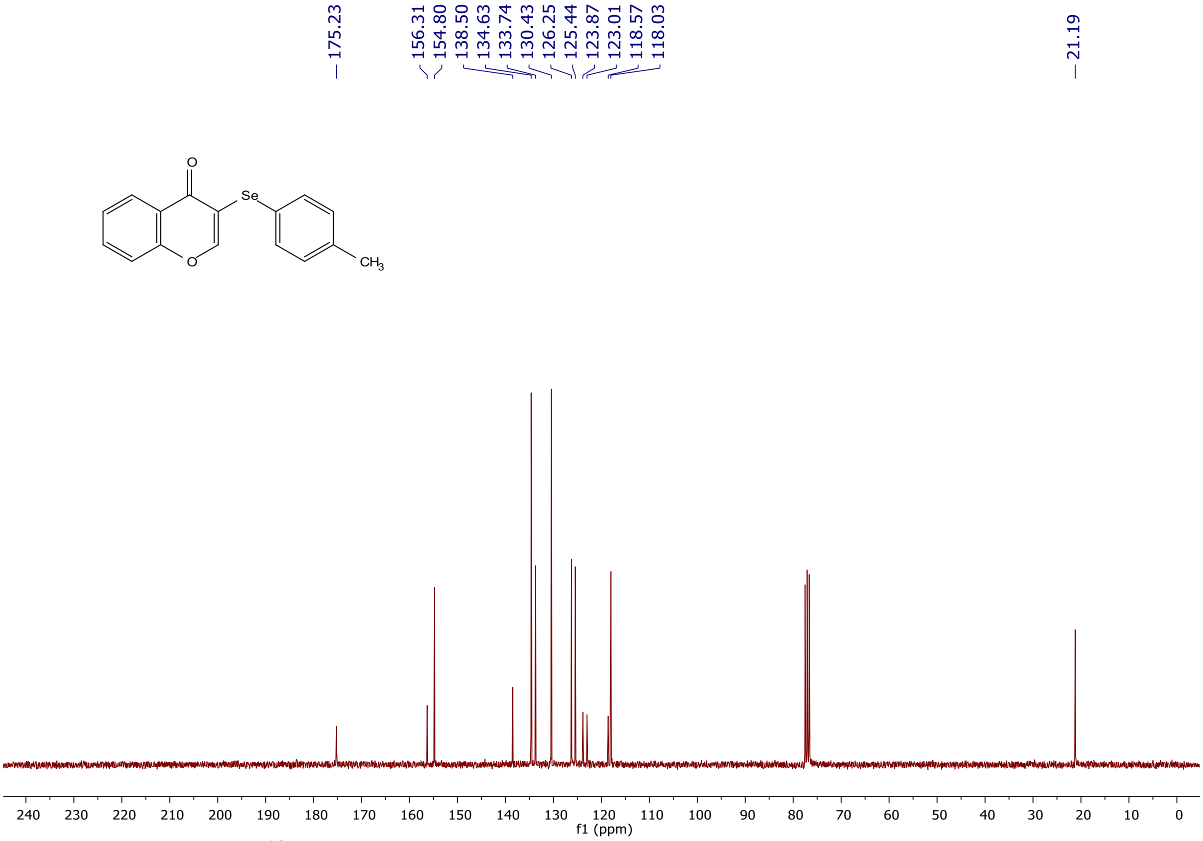

Figure S6. ^{13}C-NMR Spectra of the compound **4c**, in $CDCl_3$, 75 MHz

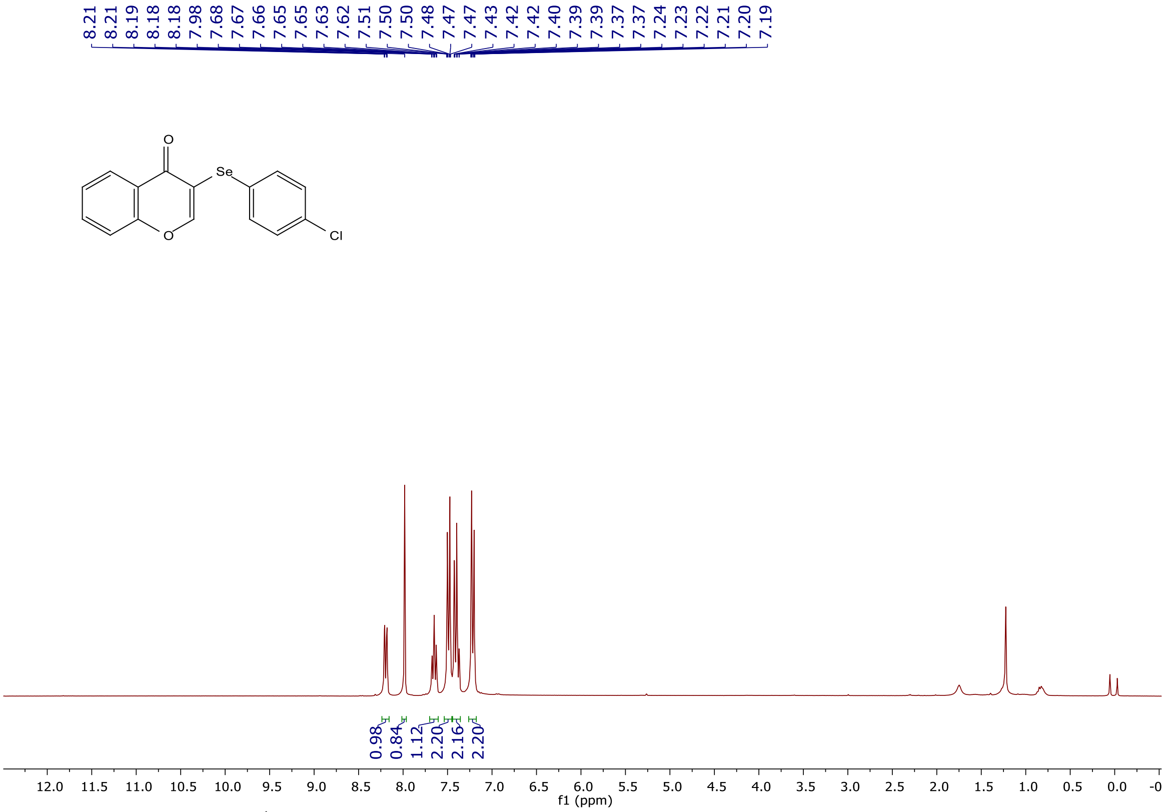

Figure S7. ^{1}H-NMR Spectra of the compound **4d,** in $CDCl_3$, 300 MHz

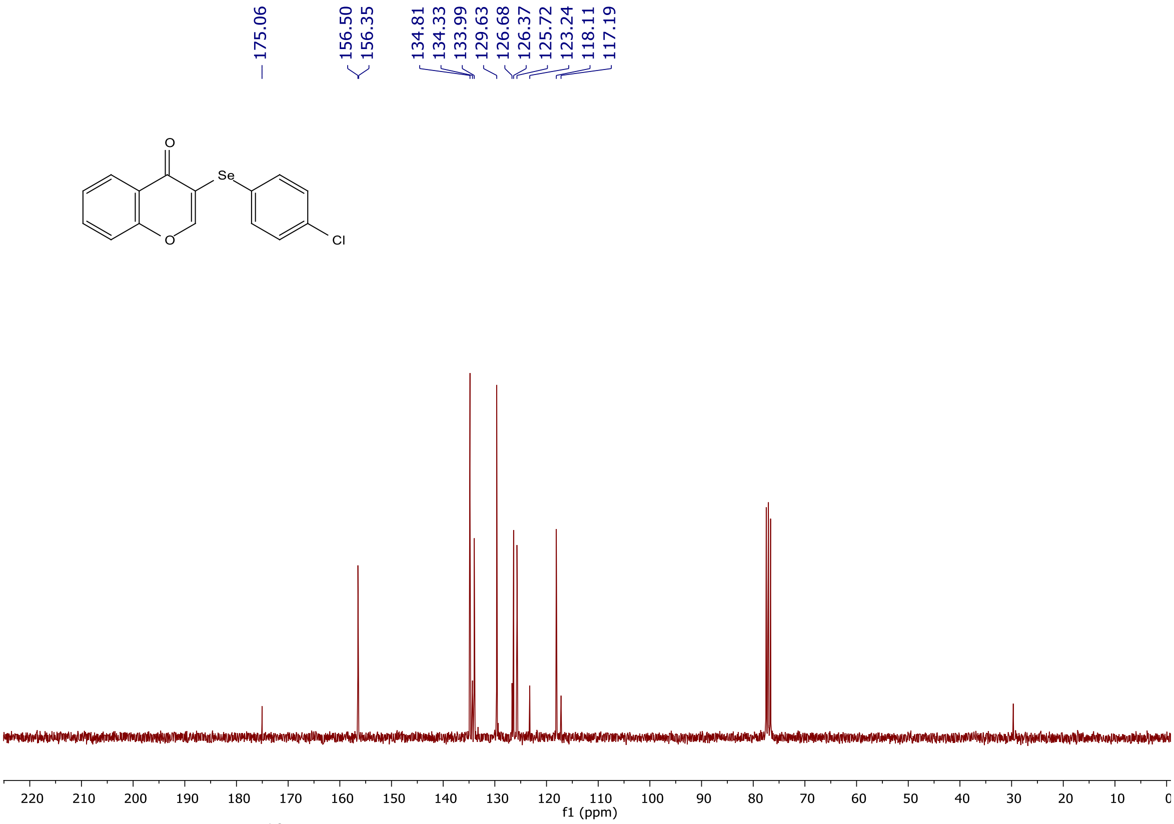

Figure S8. ^{13}C-NMR Spectra of the compound **4d**, in $CDCl_3$, 75 MHz

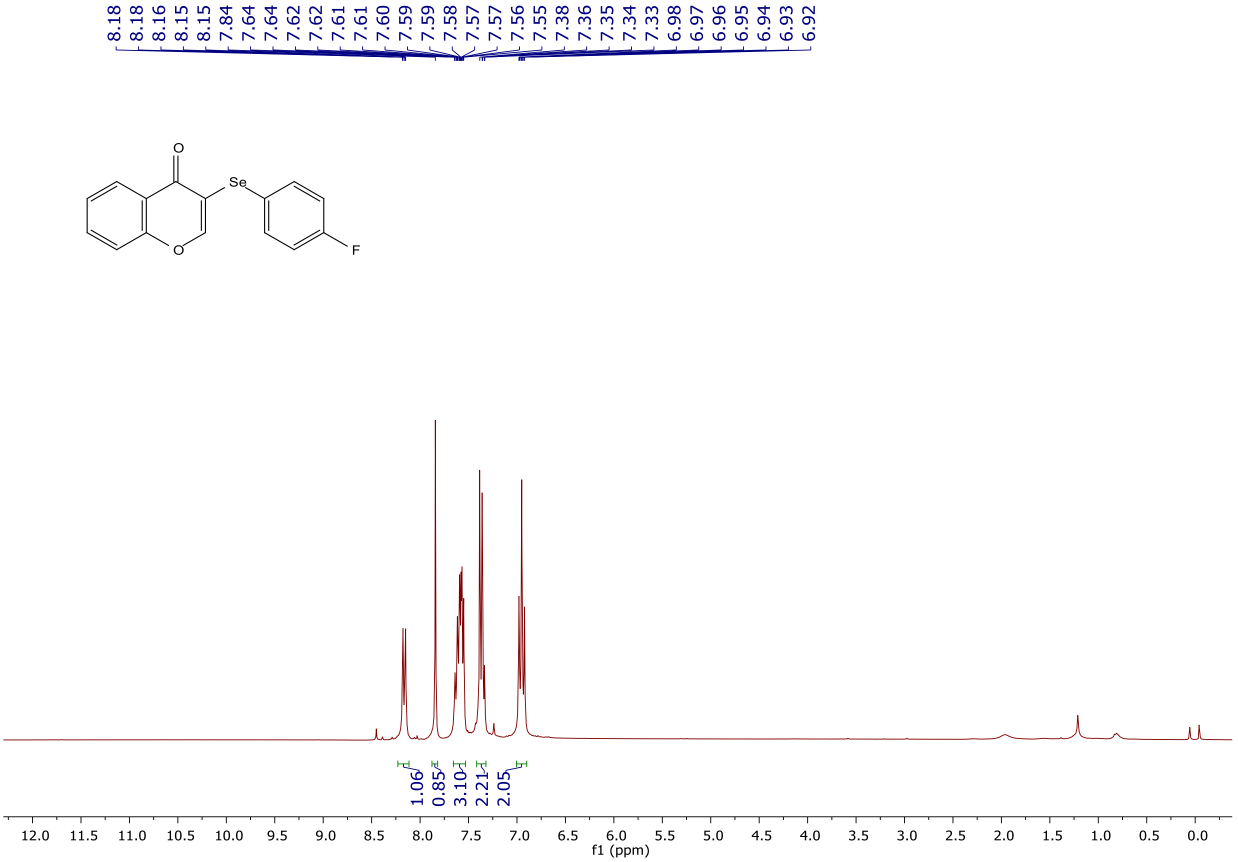

Figure S9. ^{1}H-NMR Spectra of the compound **4e,** in $CDCl_3$, 300 MHz

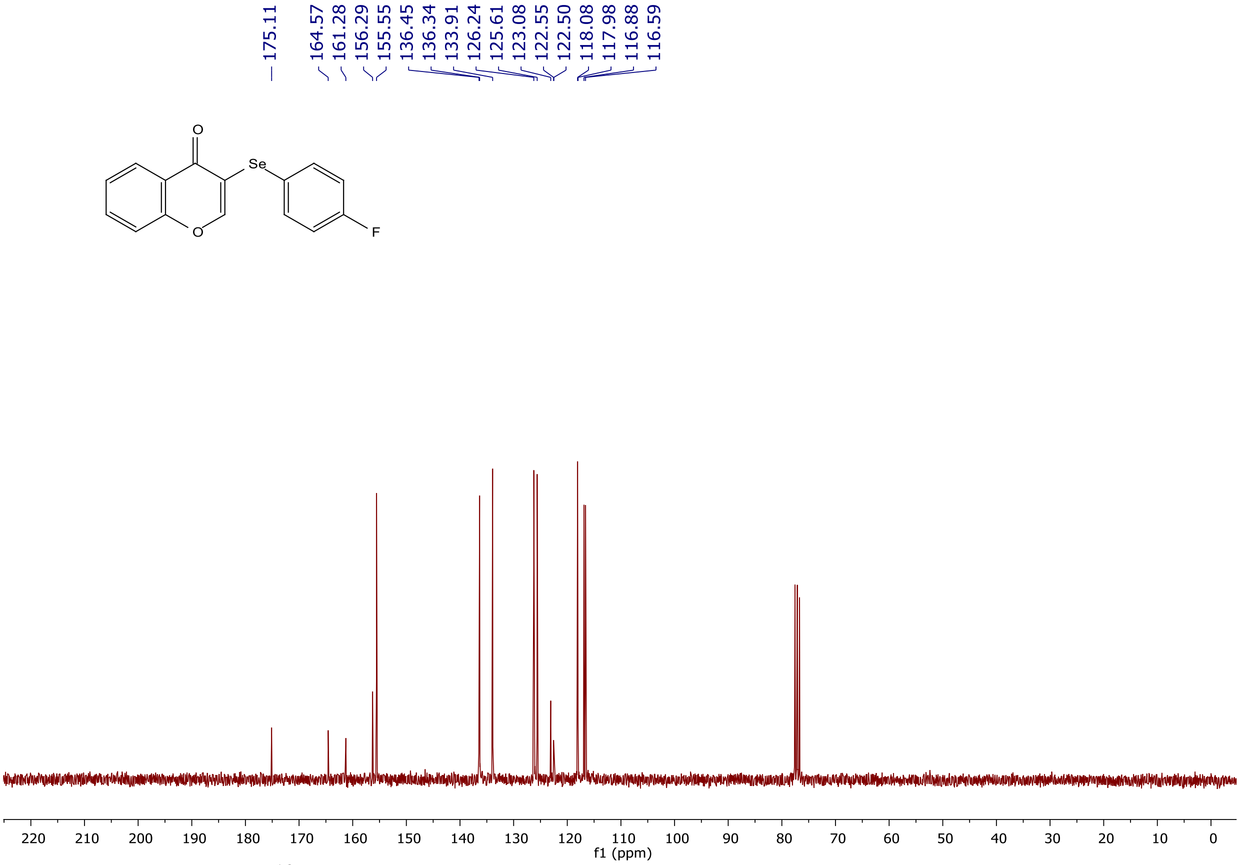

Figure S10. ^{13}C-NMR Spectra of the compound **4e**, in $CDCl_3$, 75 MHz

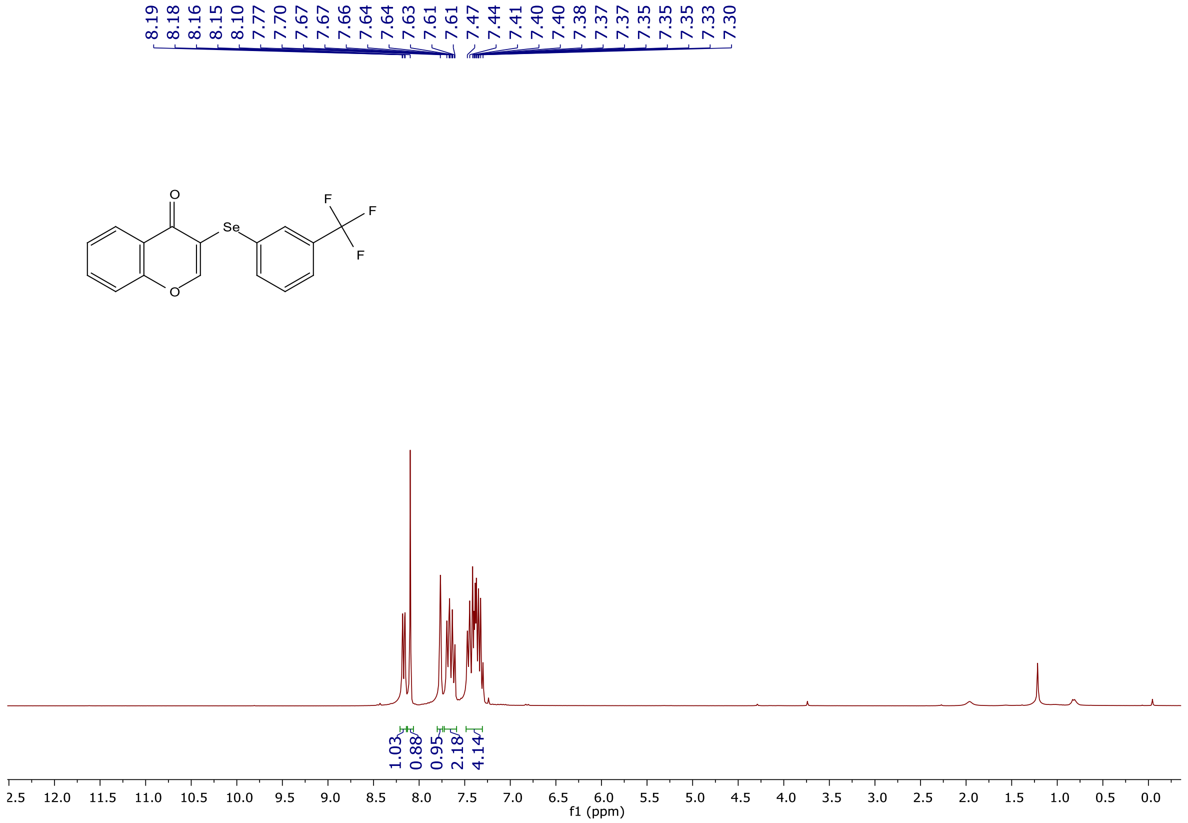

Figure S11. ^{1}H-NMR Spectra of the compound **4f,** in $CDCl_3$, 300 MHz

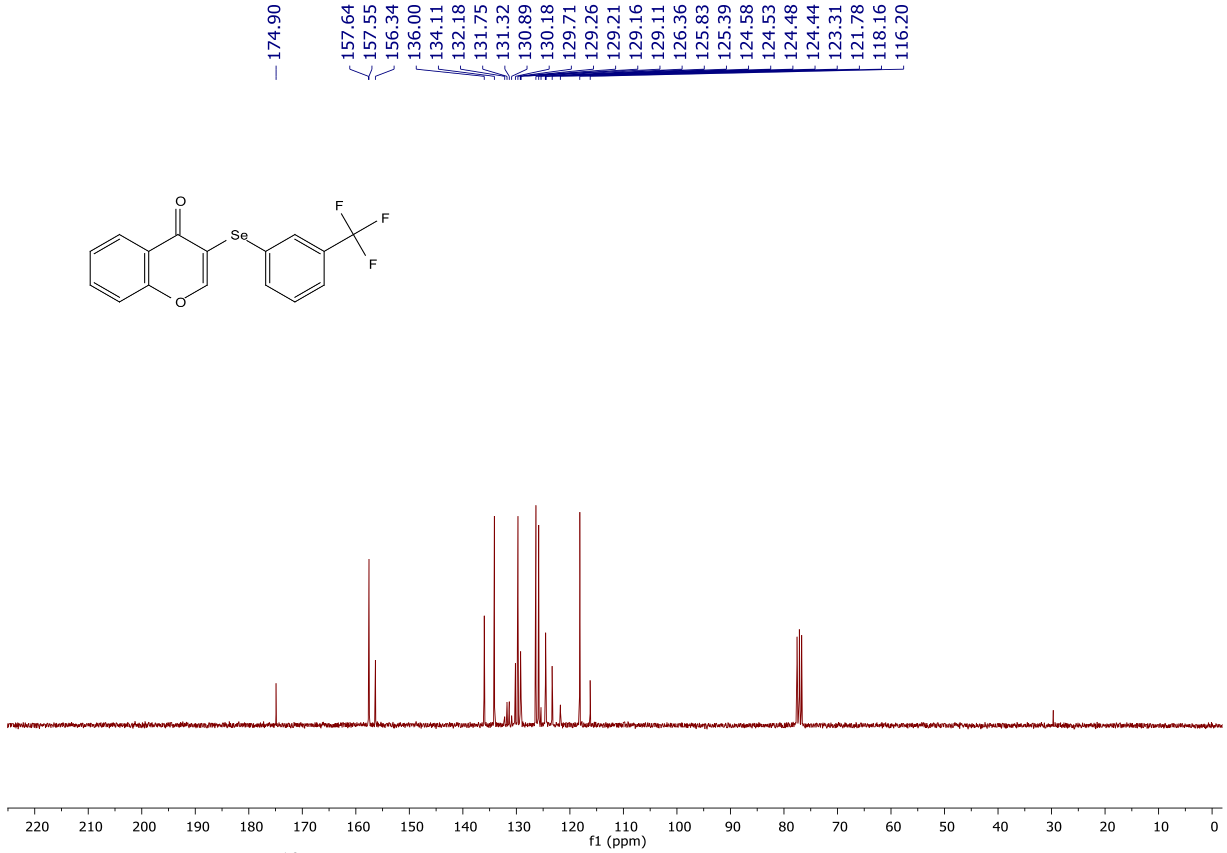

Figure S12. ^{13}C-NMR Spectra of the compound **4f**, in $CDCl_3$, 75 MHz

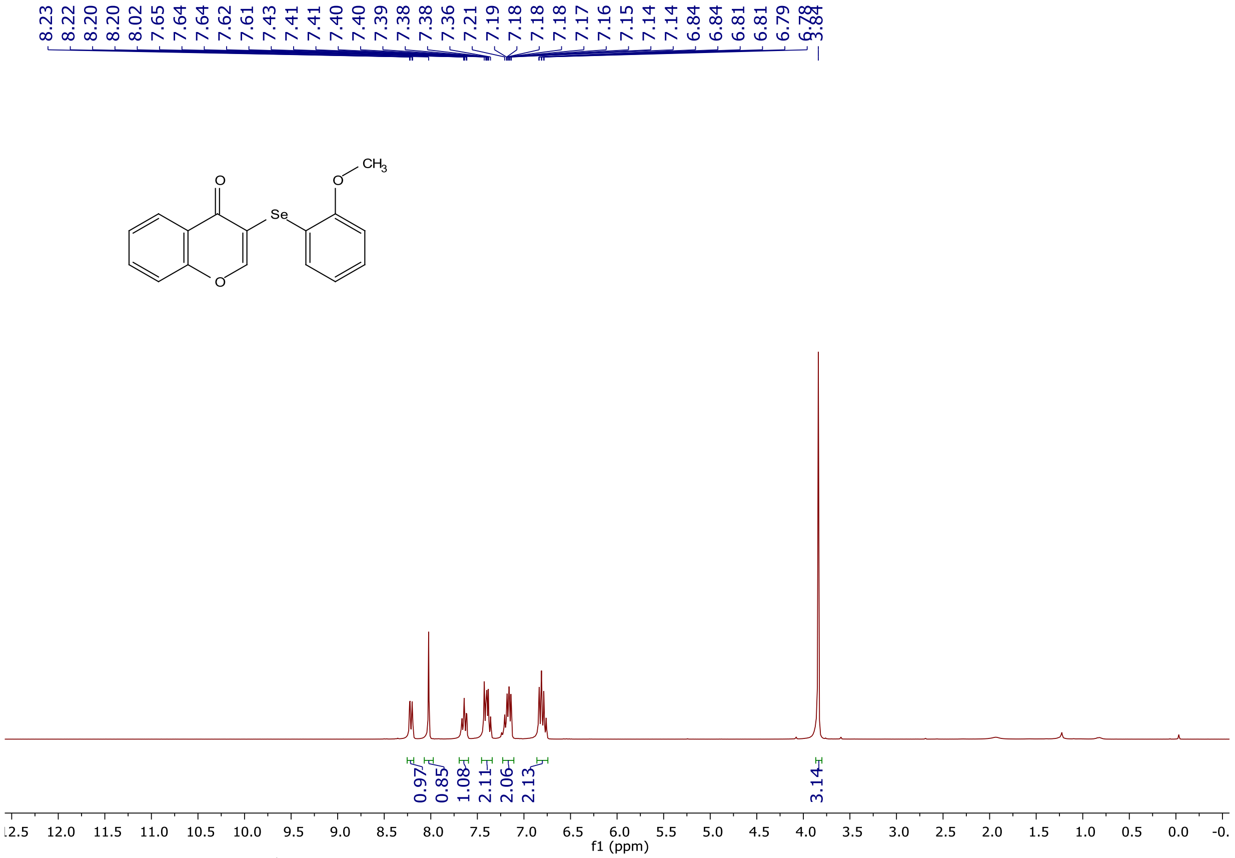

Figure S13. ^{1}H-NMR Spectra of the compound **4g,** in $CDCl_3$, 300 MHz

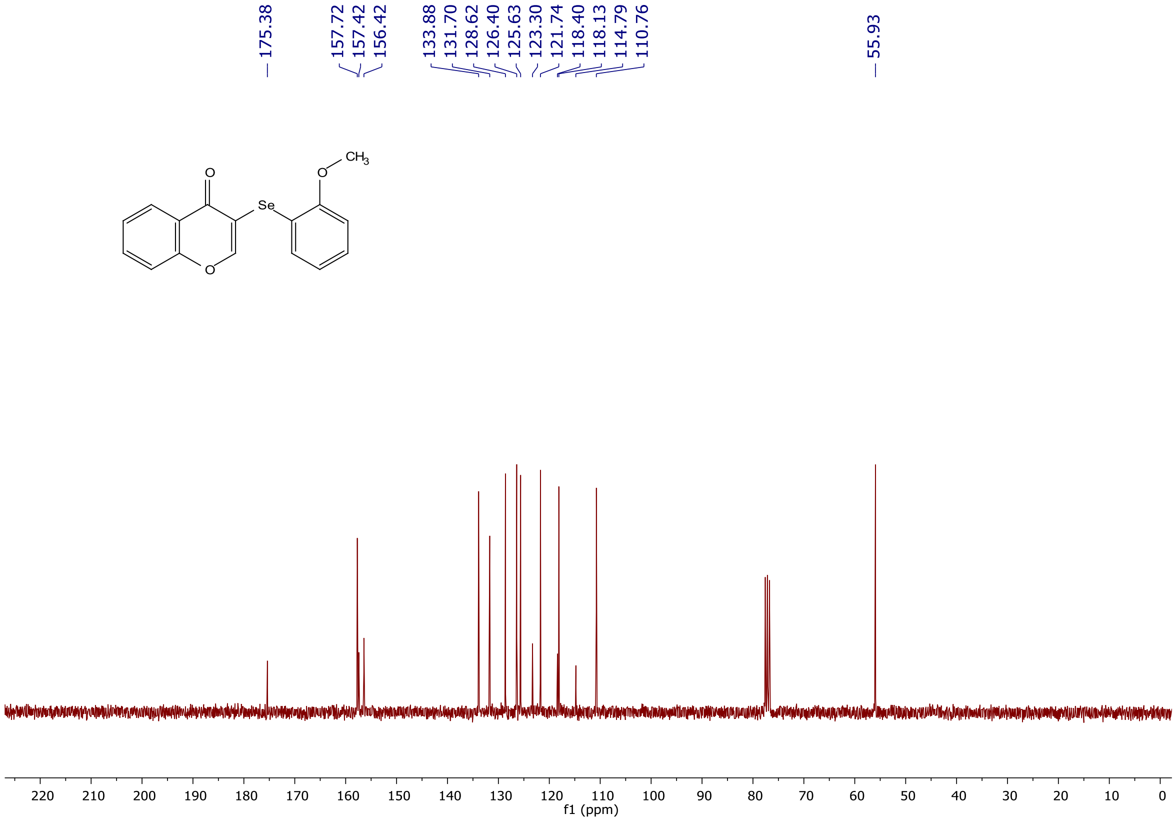

Figure S14. ^{13}C-NMR Spectra of the compound **4g**, in $CDCl_3$, 75 MHz

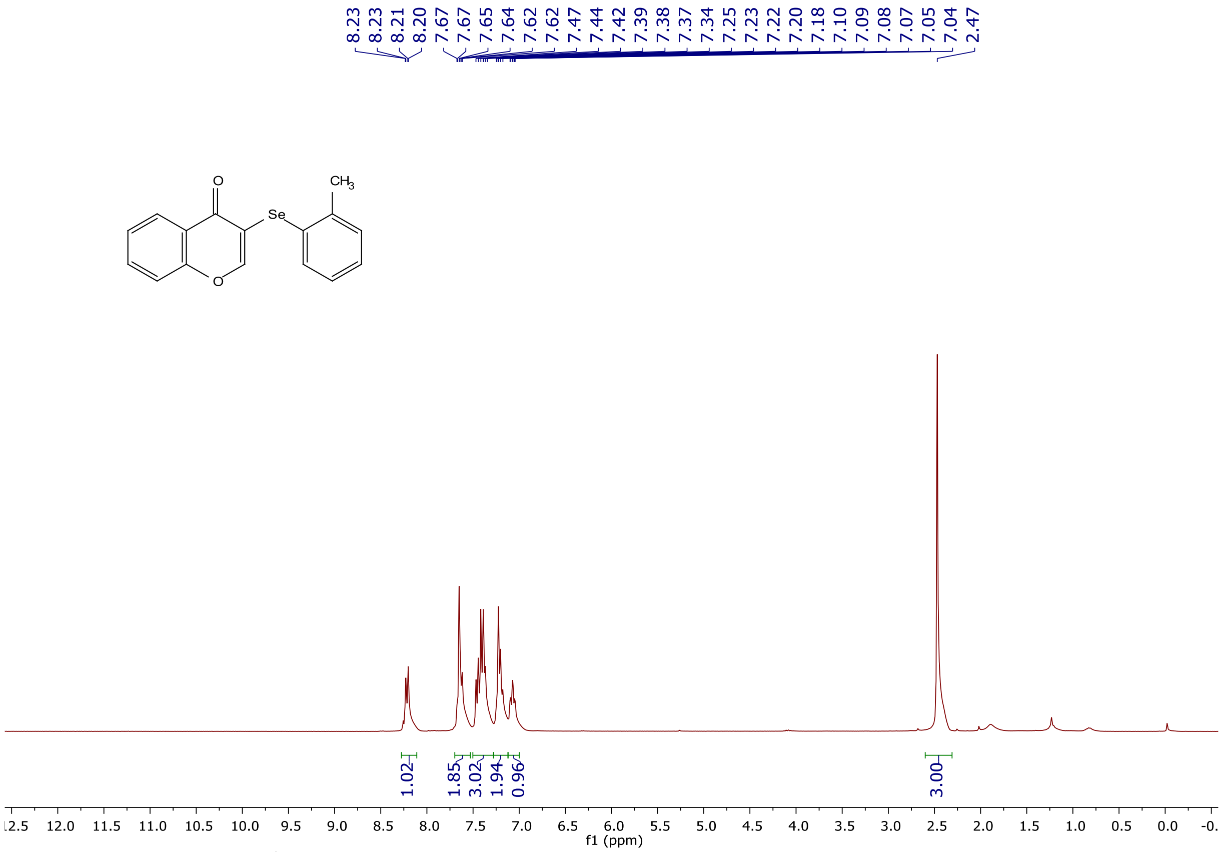

Figure S15. ^{1}H-NMR Spectra of the compound **4h,** in $CDCl_3$, 300 MHz

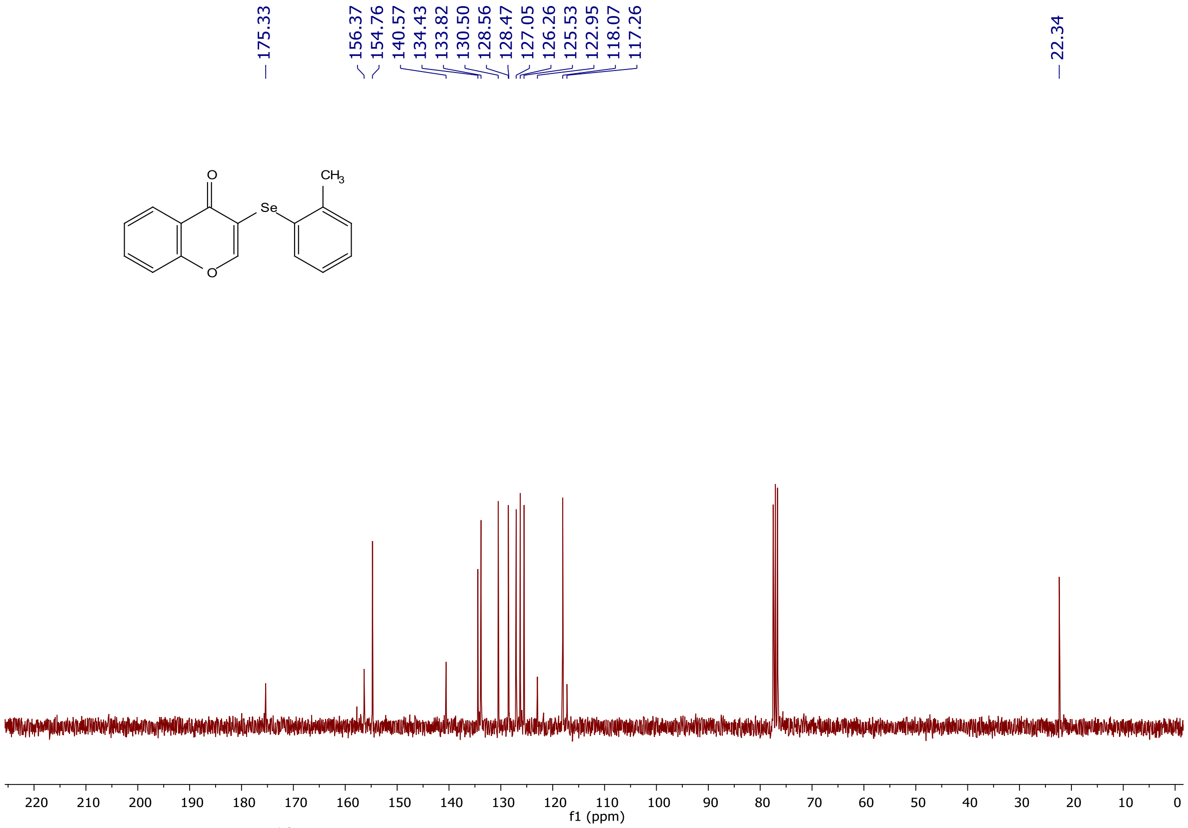

Figure S16. ^{13}C-NMR Spectra of the compound **4h**, in $CDCl_3$, 75 MHz

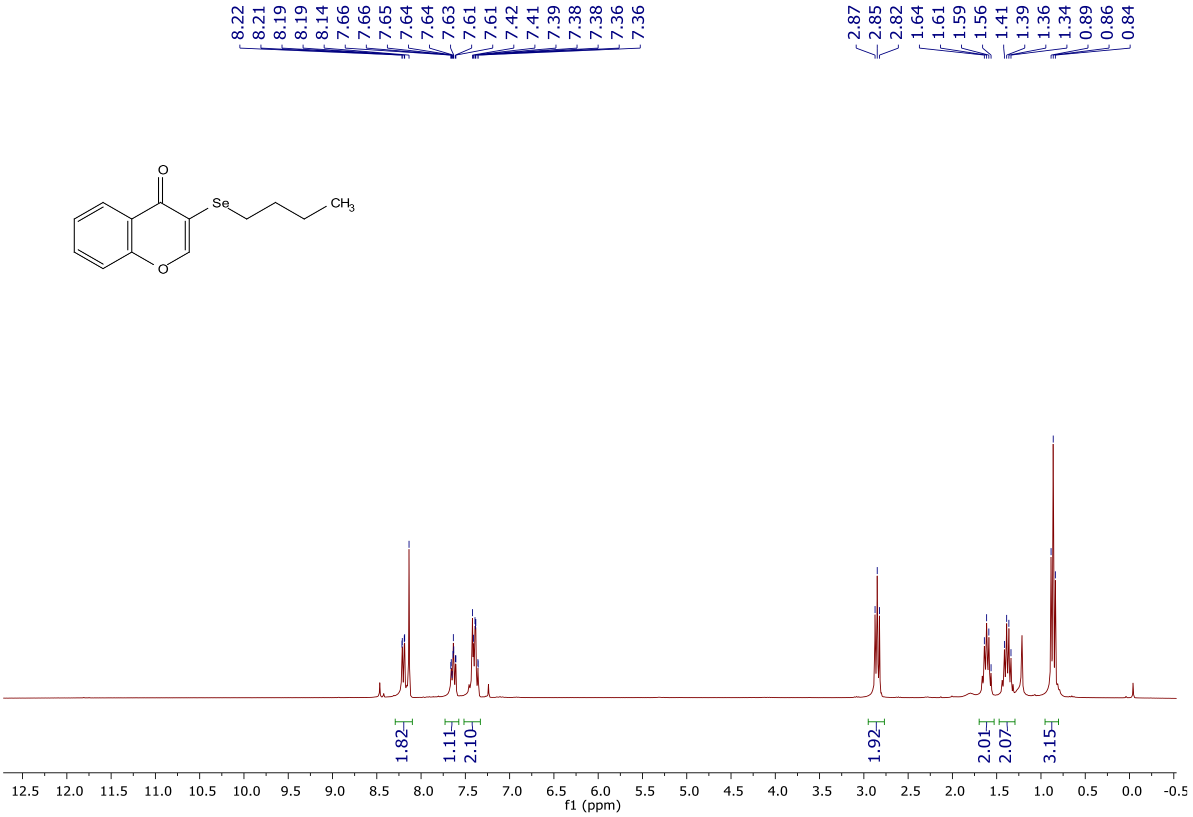

Figure S17. ^{1}H-NMR Spectra of the compound **4i,** in $CDCl_3$, 300 MHz

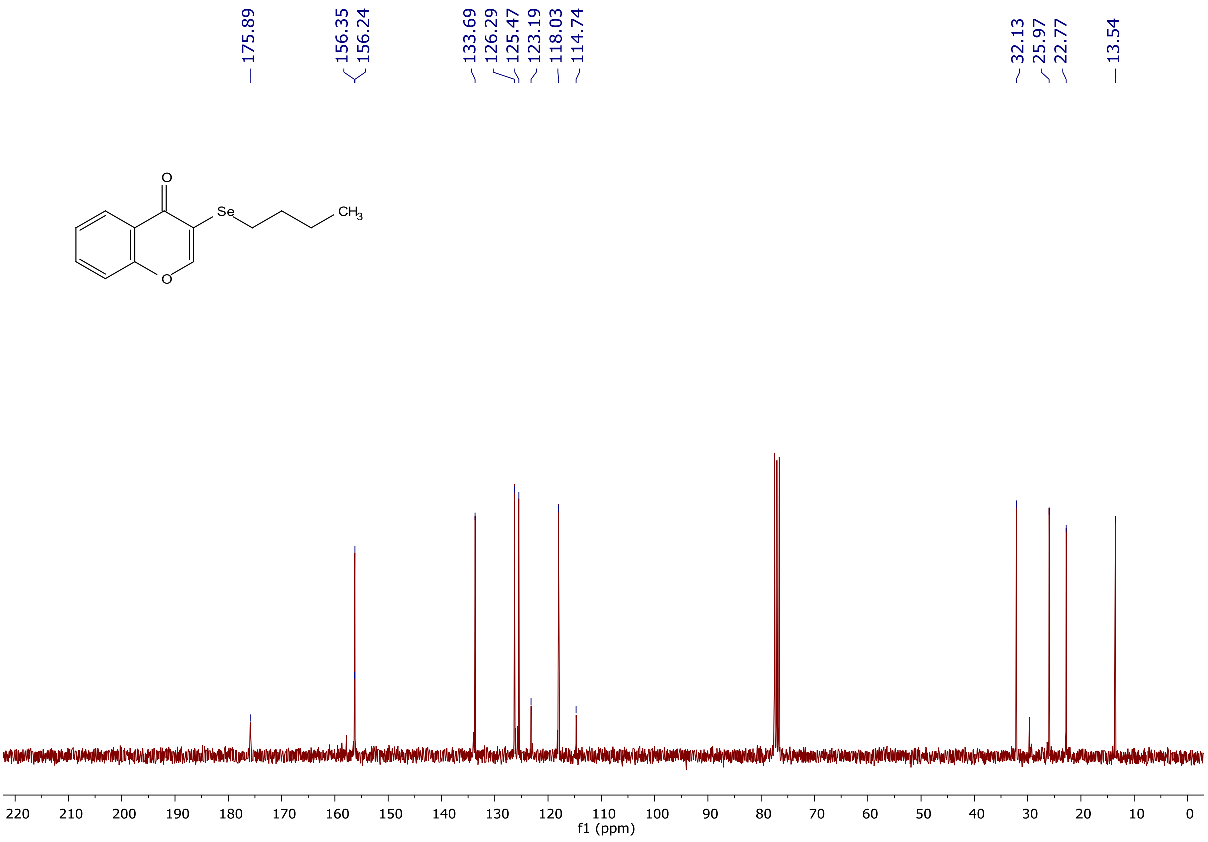

Figure S18. ^{13}C-NMR Spectra of the compound **4i**, in $CDCl_3$, 75 MHz

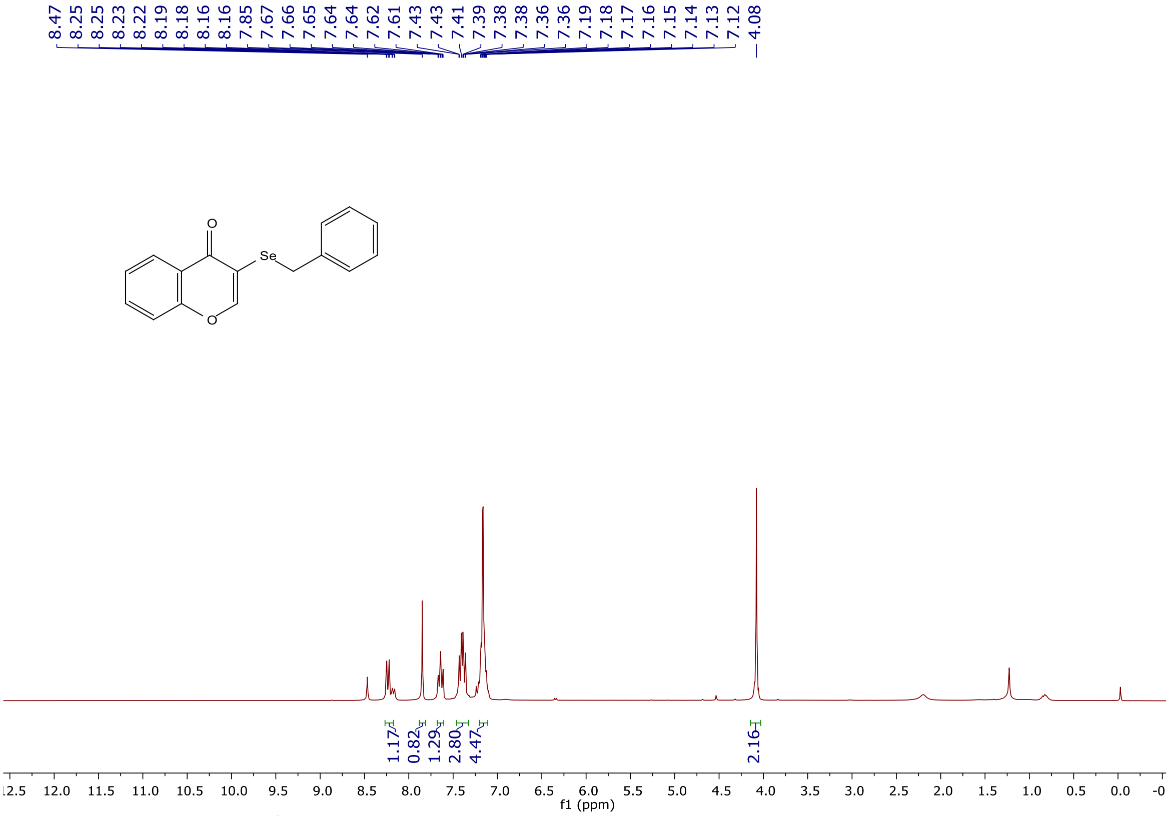

Figure S19. ^{1}H-NMR Spectra of the compound **4j,** in $CDCl_3$, 300 MHz

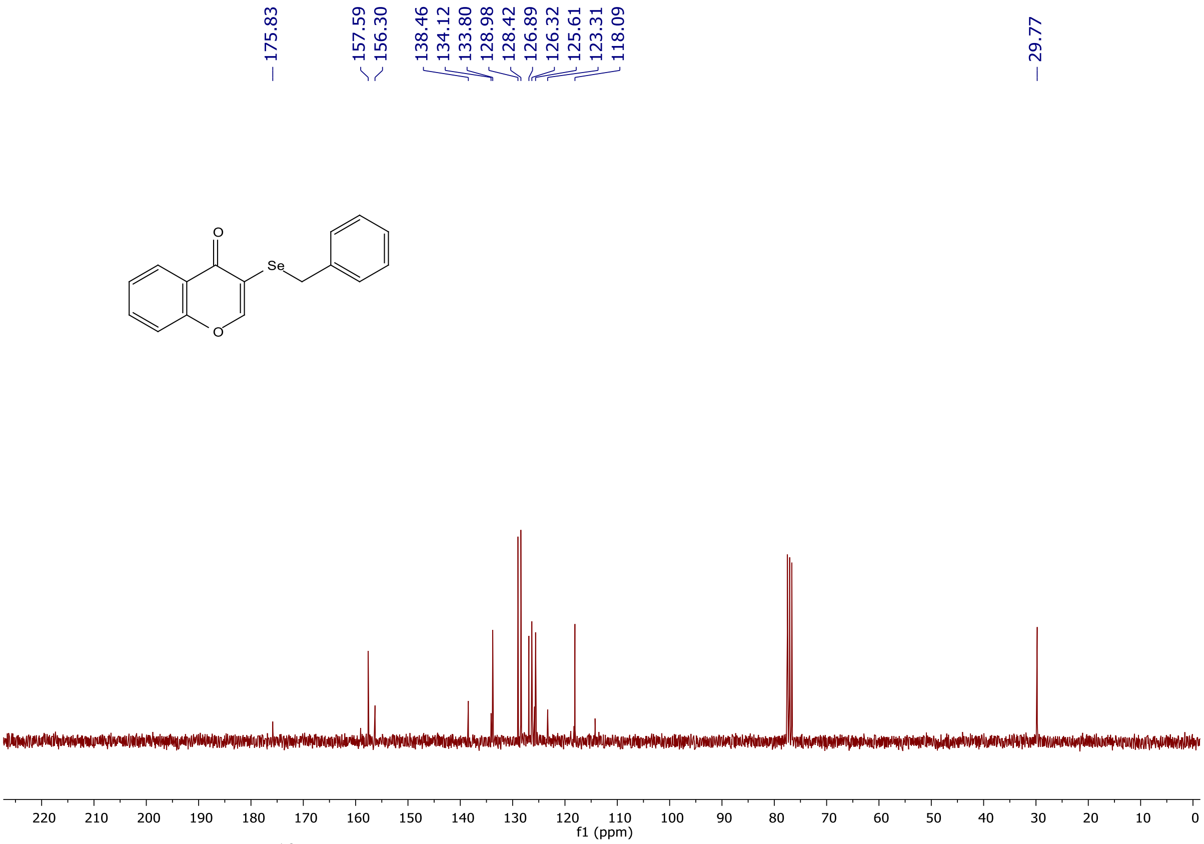

Figure S20. ^{13}C-NMR Spectra of the compound **4j**, in $CDCl_3$, 75 MHz

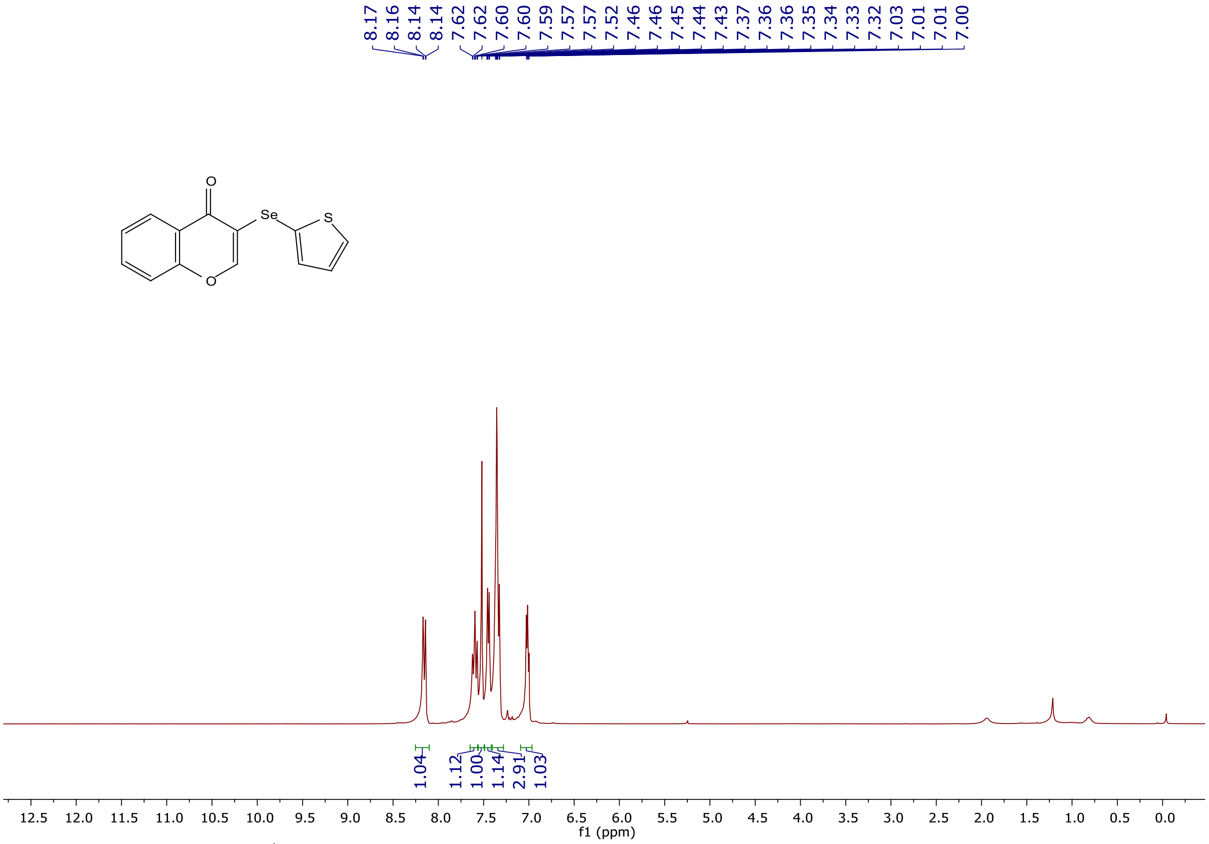

Figure S21. ^{1}H-NMR Spectra of the compound **4k,** in $CDCl_3$, 300 MHz

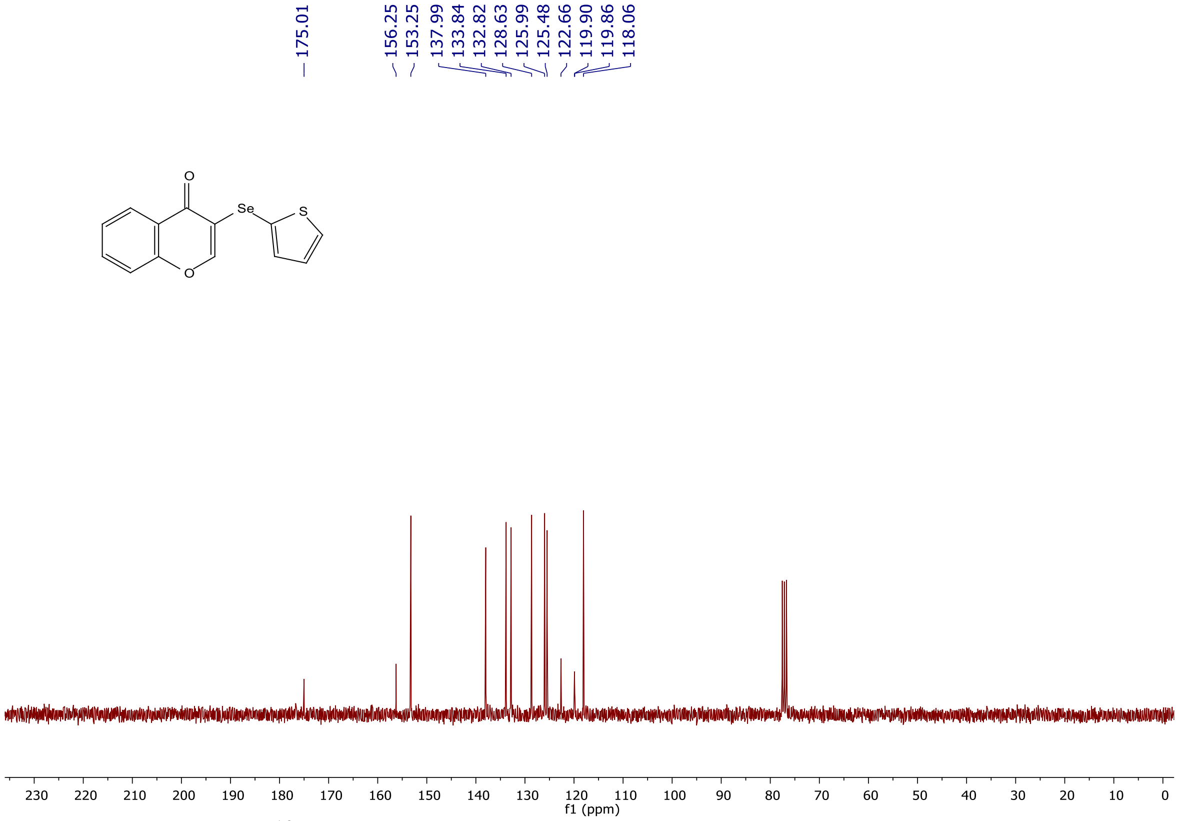

Figure S22. ^{13}C-NMR Spectra of the compound **4k**, in $CDCl_3$, 75 MHz

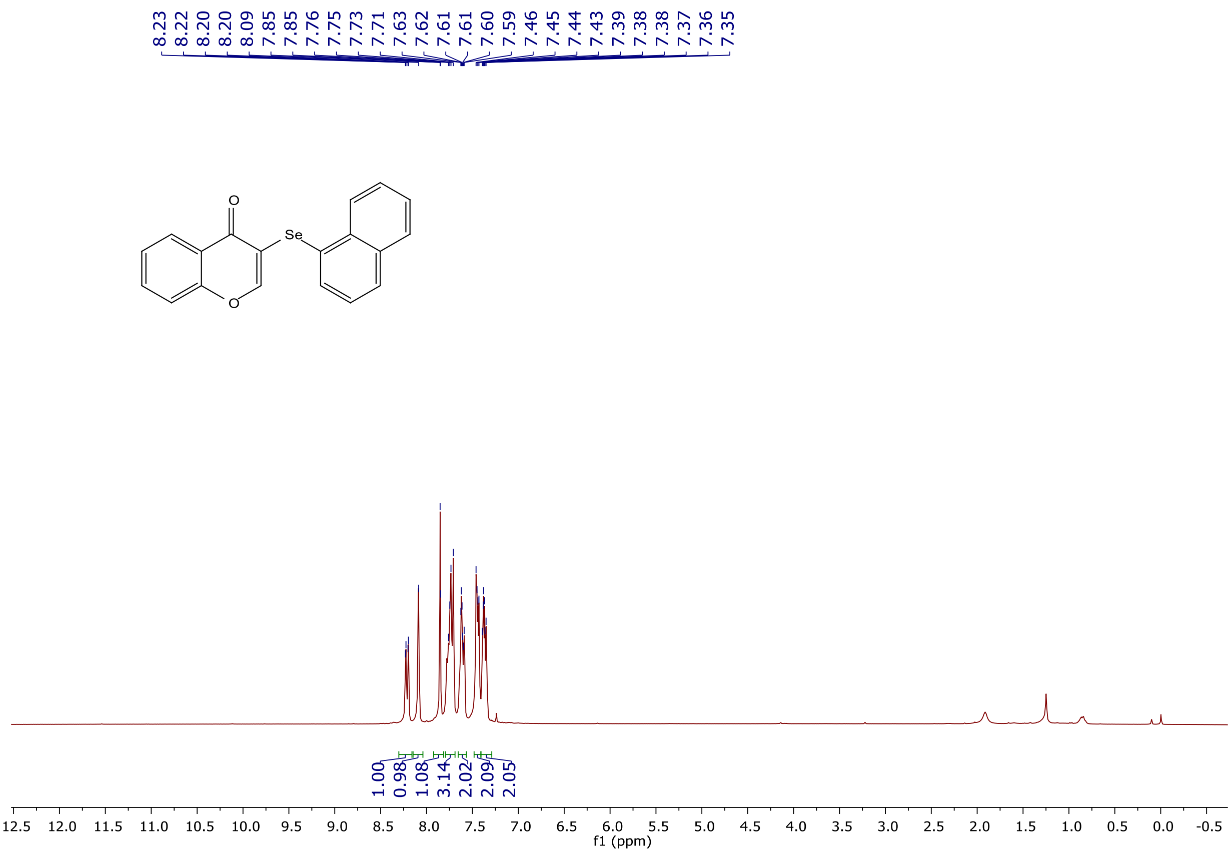

Figure S23. ^{1}H-NMR Spectra of the compound **4l,** in $CDCl_3$, 300 MHz

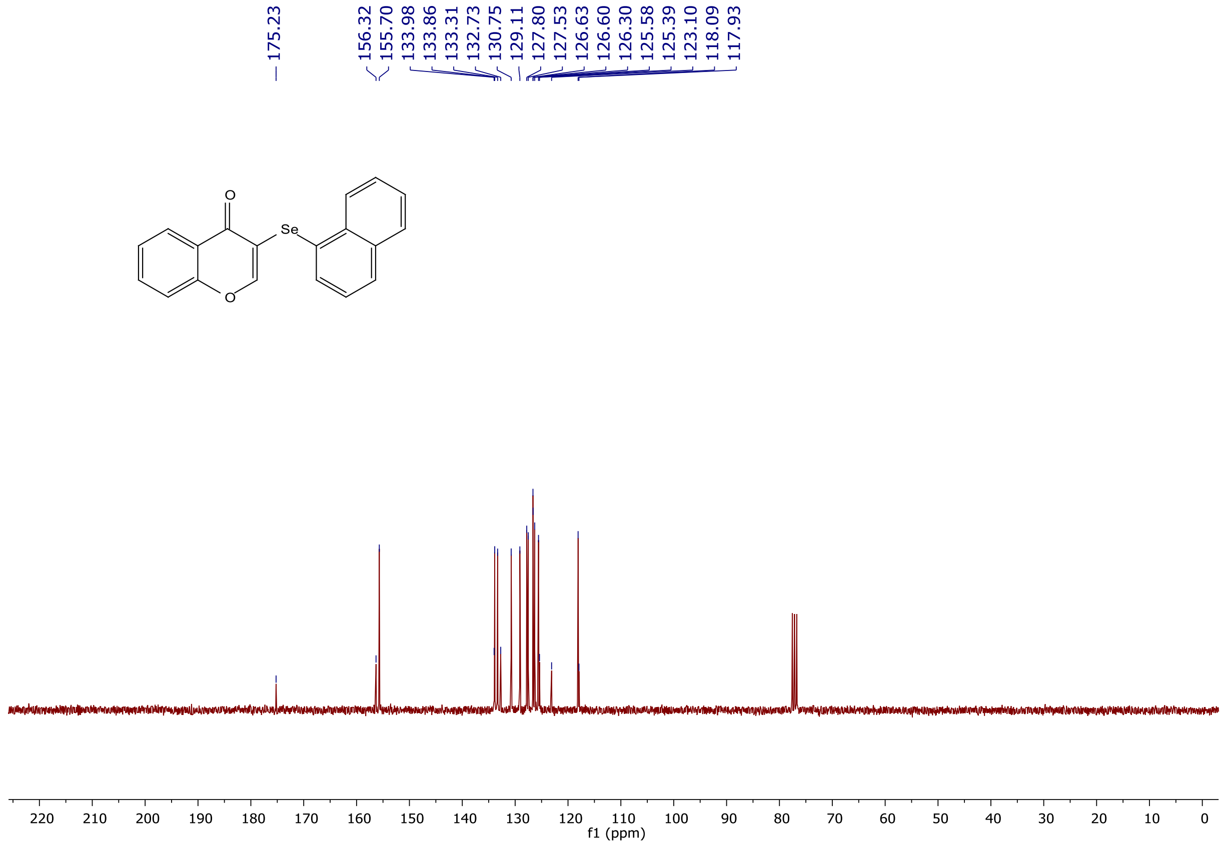

Figure S24. ^{13}C-NMR Spectra of the compound **4l**, in $CDCl_3$, 75 MHz

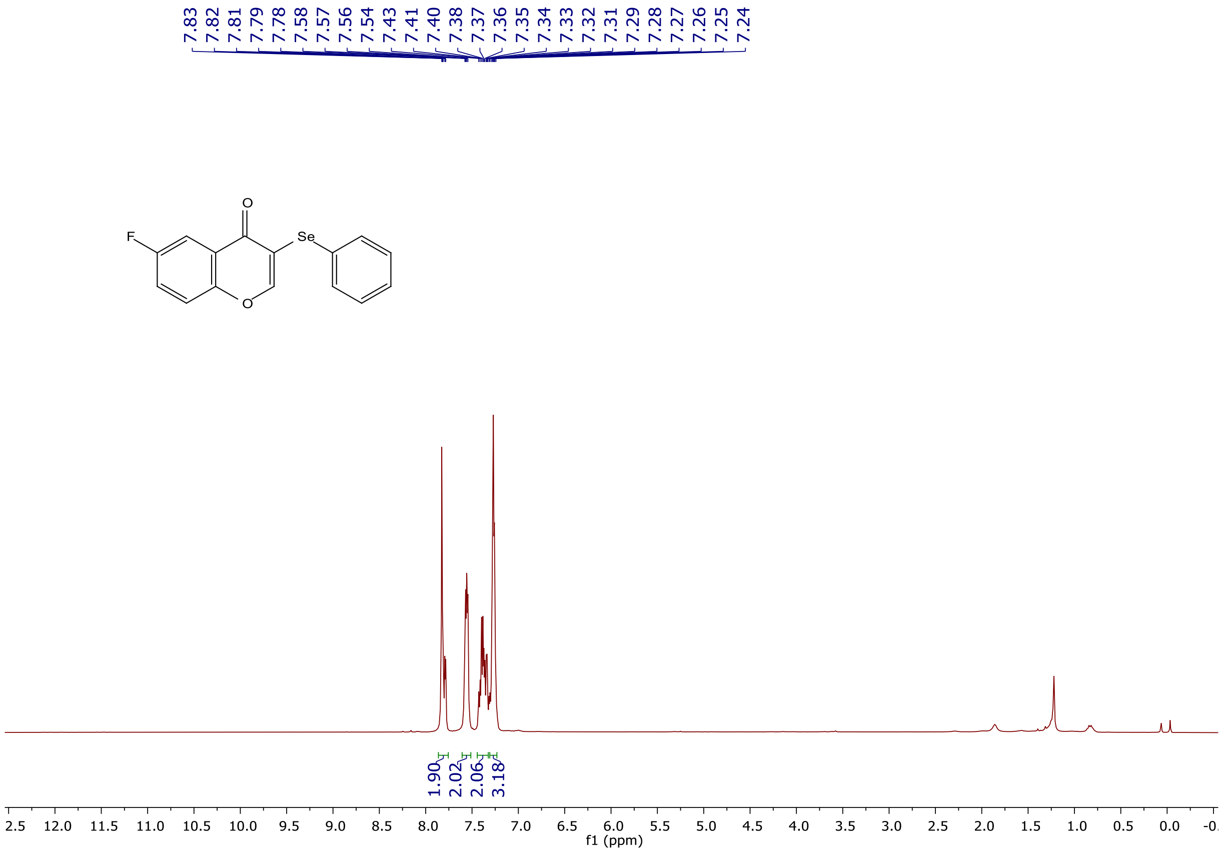

Figure S25. ^{1}H-NMR Spectra of the compound **4m,** in $CDCl_3$, 300 MHz

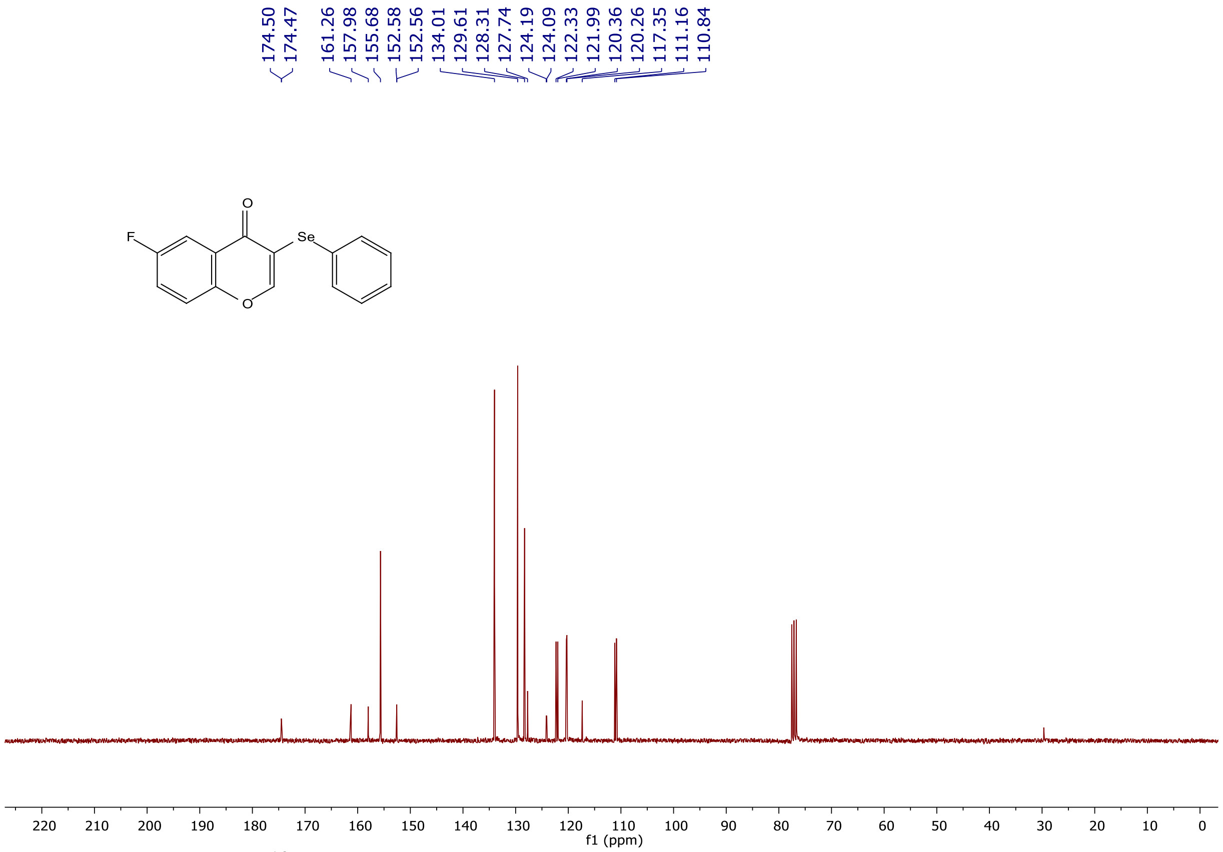

Figure S26. ^{13}C-NMR Spectra of the compound **4m**, in $CDCl_3$, 75 MHz

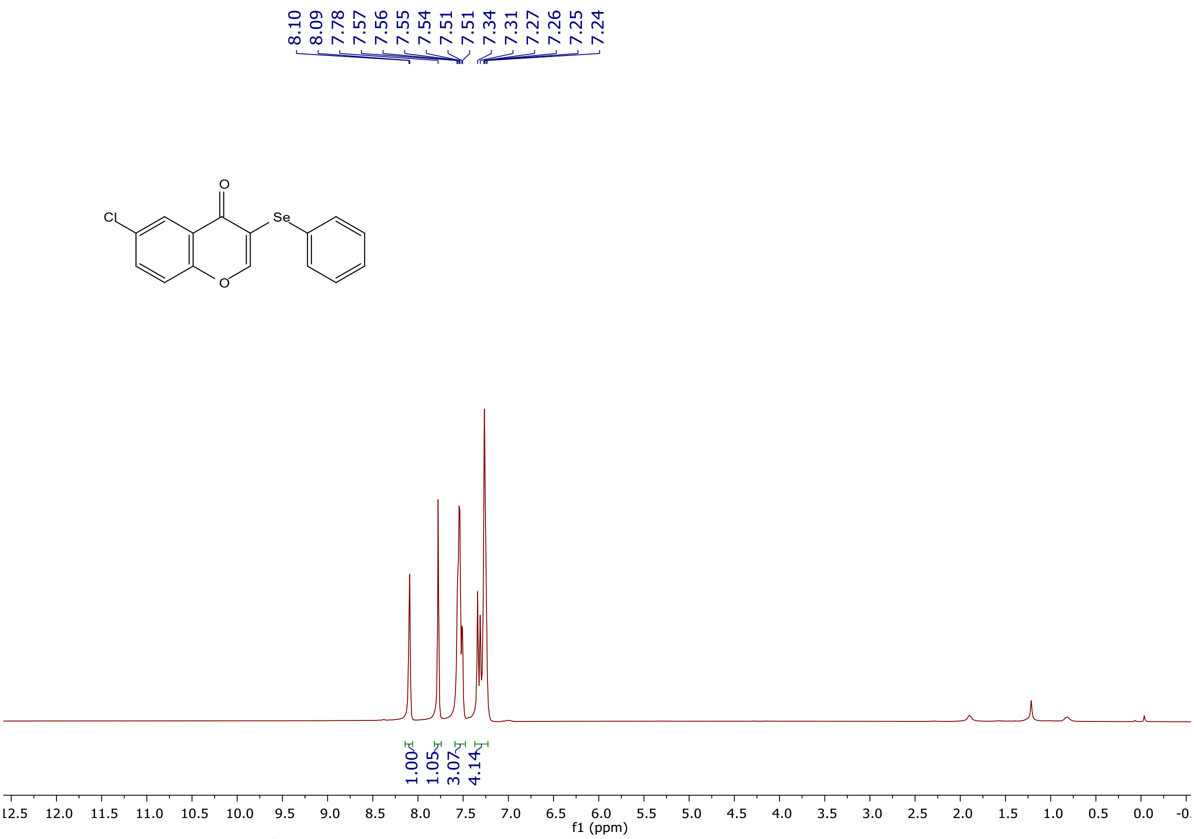

Figure S27. ^{1}H-NMR Spectra of the compound **4n,** in $CDCl_3$, 300 MHz

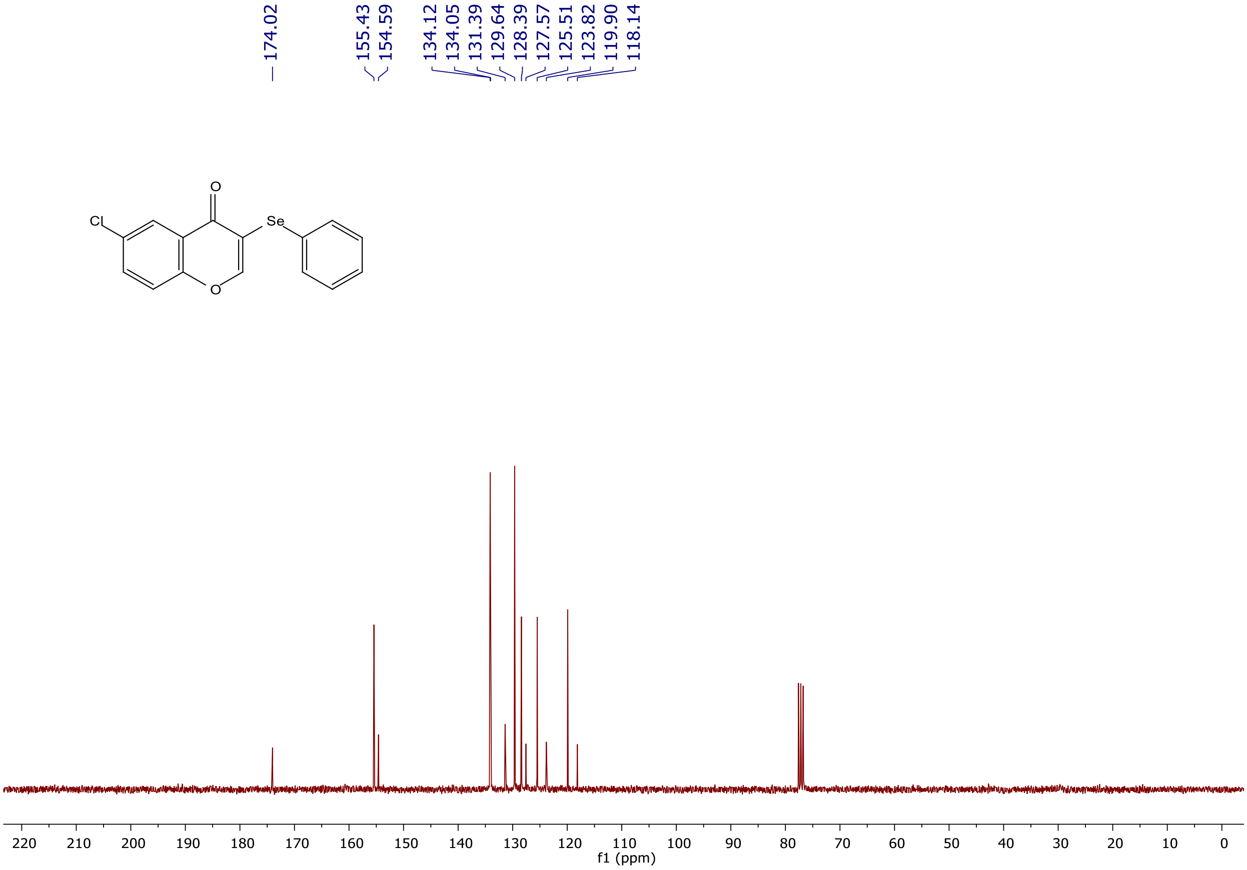

Figure S28. ^{13}C-NMR Spectra of the compound **4n**, in $CDCl_3$, 75 MHz

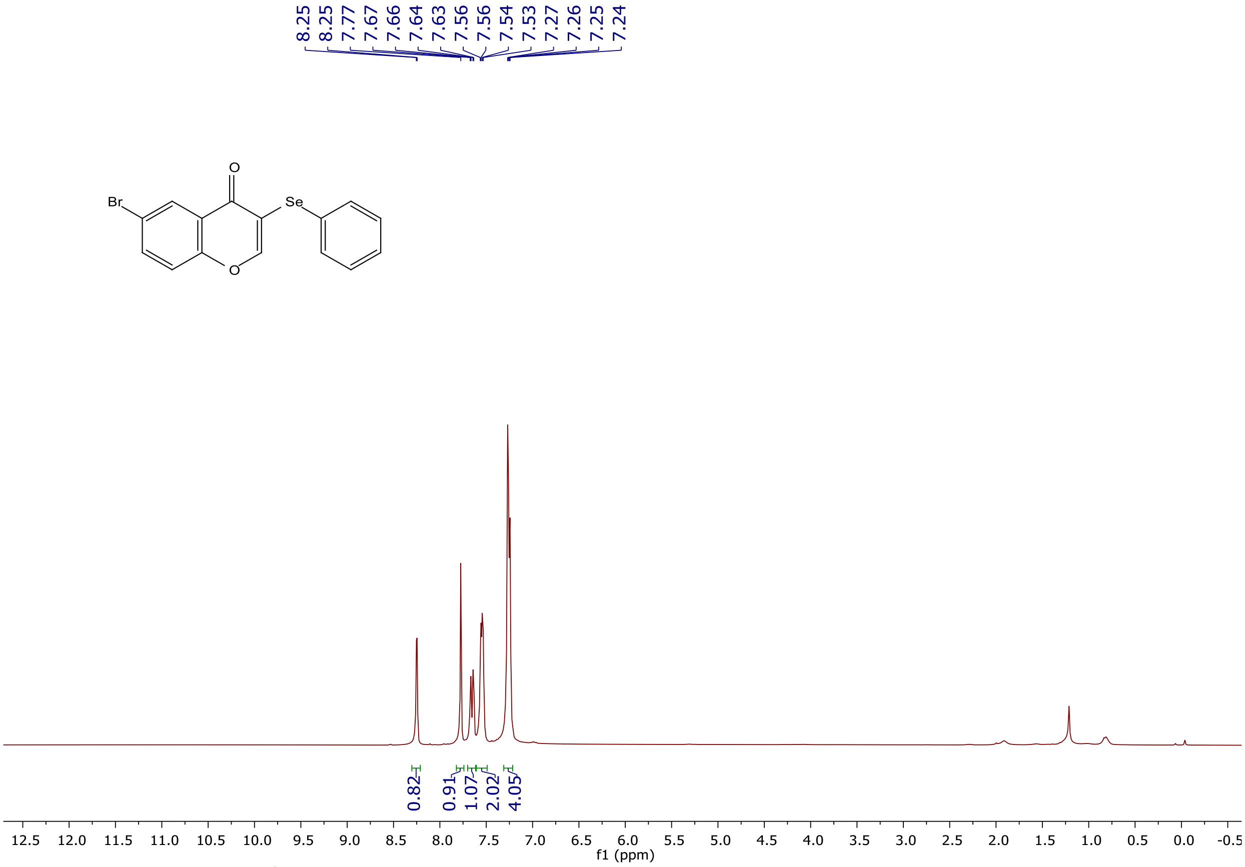

Figure S29. ^{1}H-NMR Spectra of the compound **4o,** in $CDCl_3$, 300 MHz

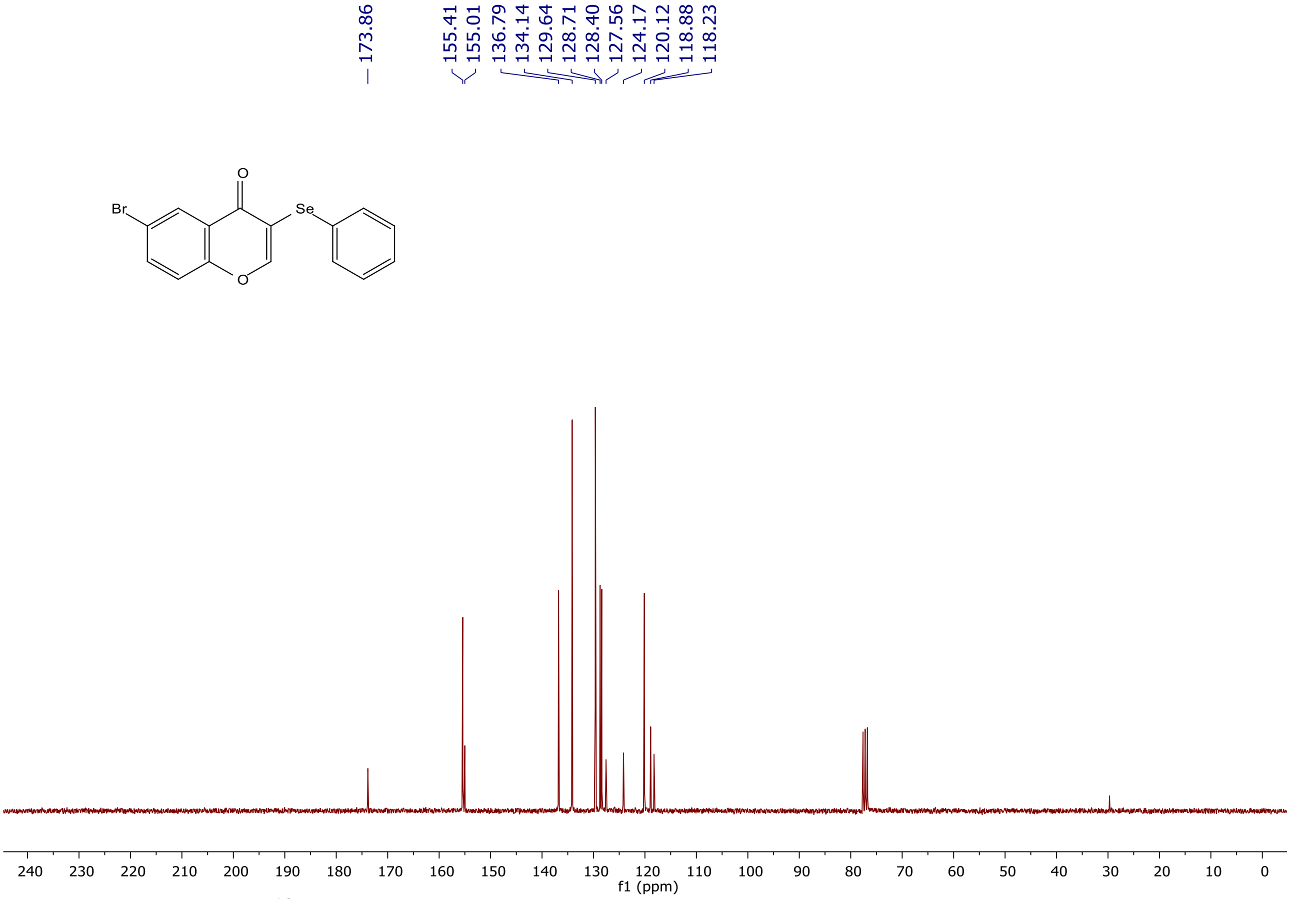

Figure S30. ^{13}C-NMR Spectra of the compound **4o**, in $CDCl_3$, 75 MHz

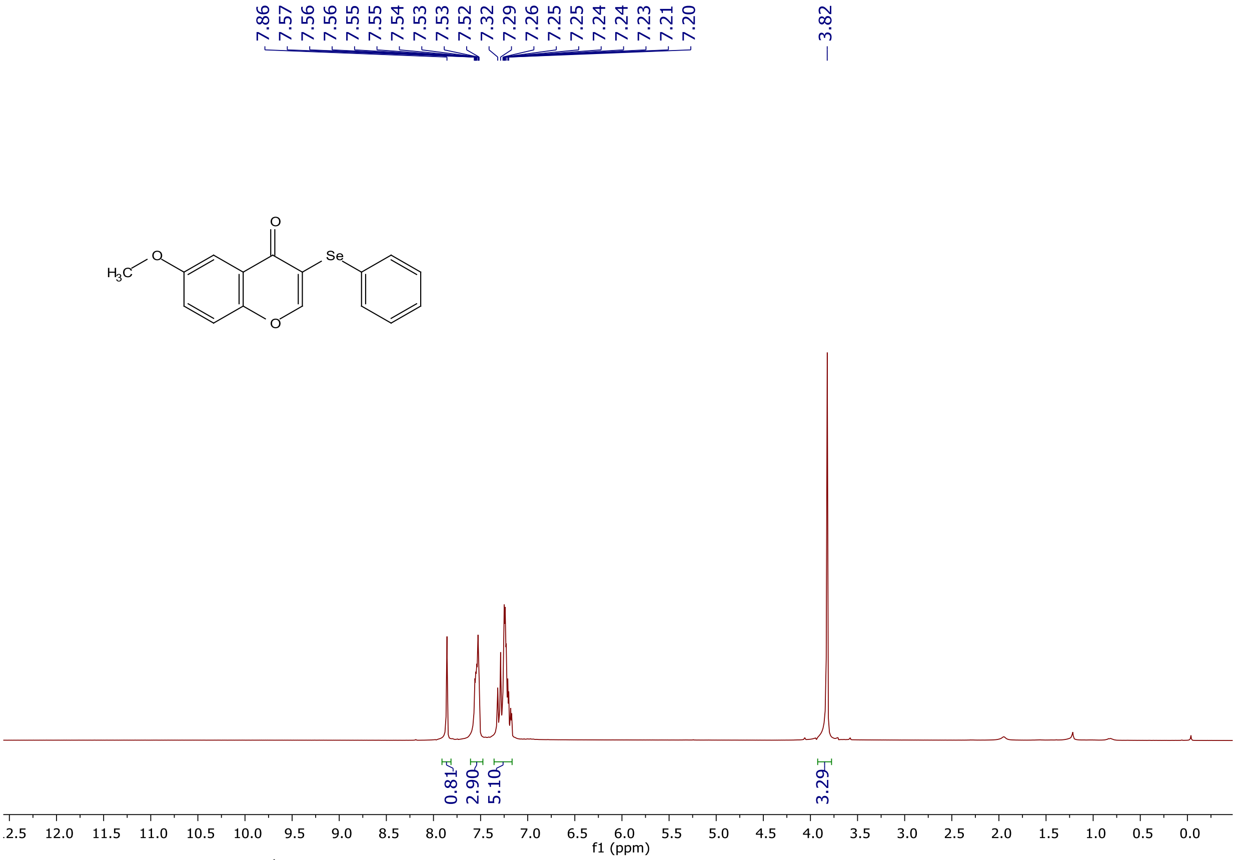

Figure S31. ^{1}H-NMR Spectra of the compound **4p,** in $CDCl_3$, 300 MHz

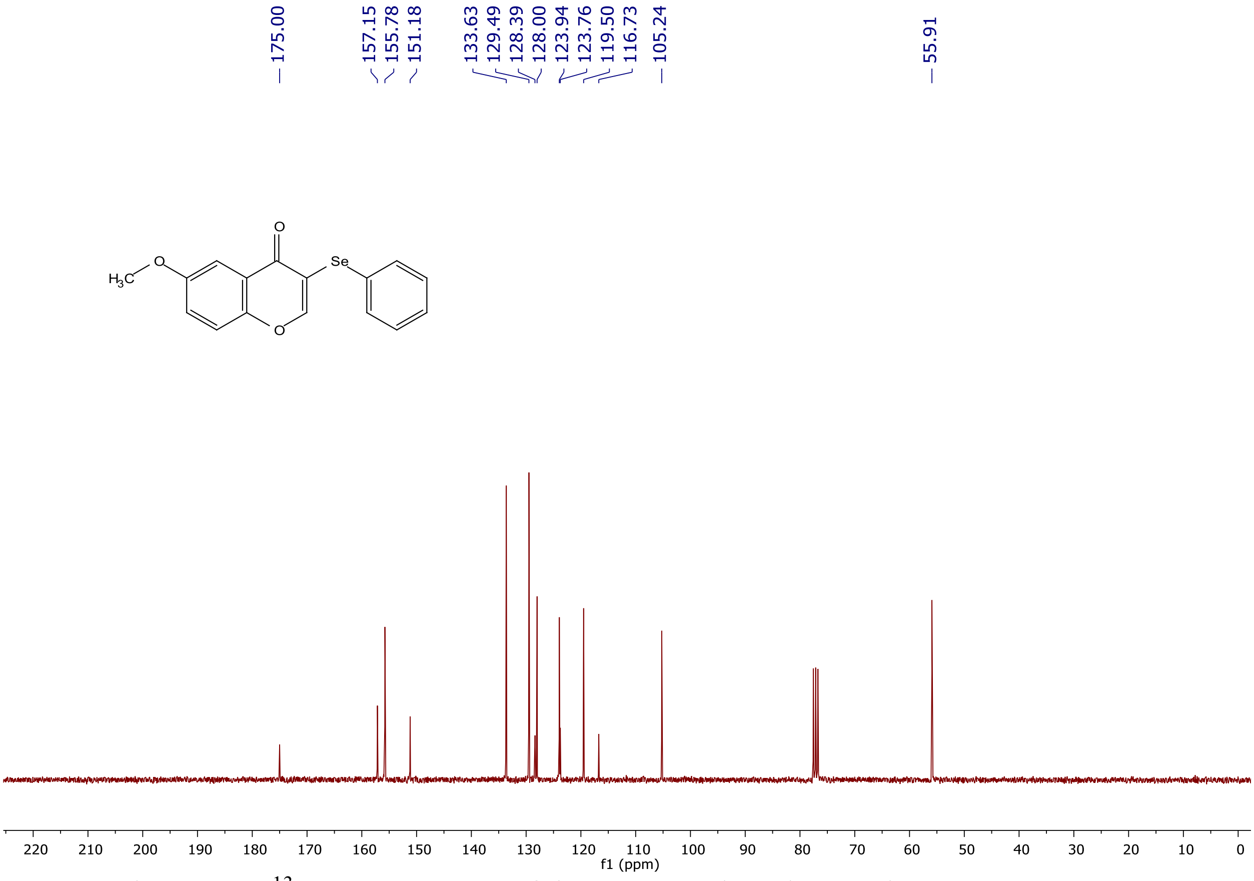

Figure S32. ^{13}C-NMR Spectra of the compound **4p**, in $CDCl_3$, 75 MHz

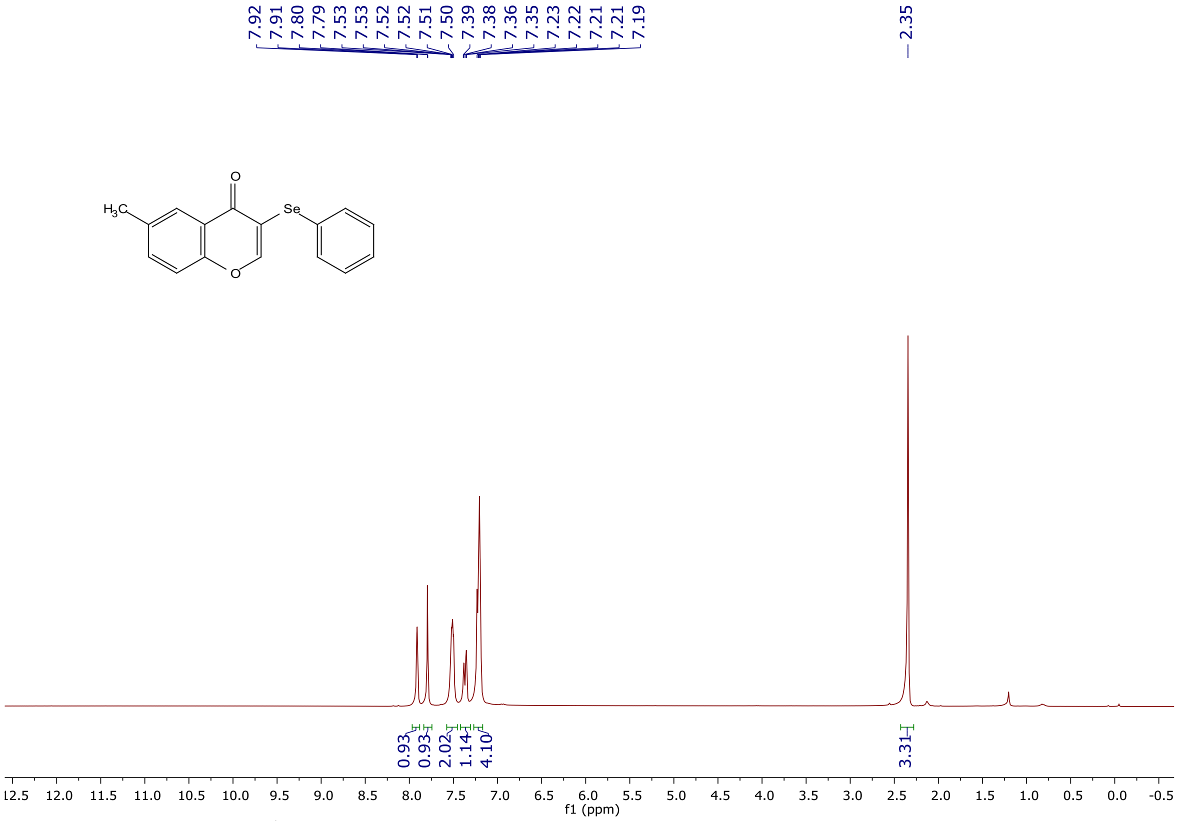

Figure S33. ^{1}H-NMR Spectra of the compound **4q,** in $CDCl_3$, 300 MHz

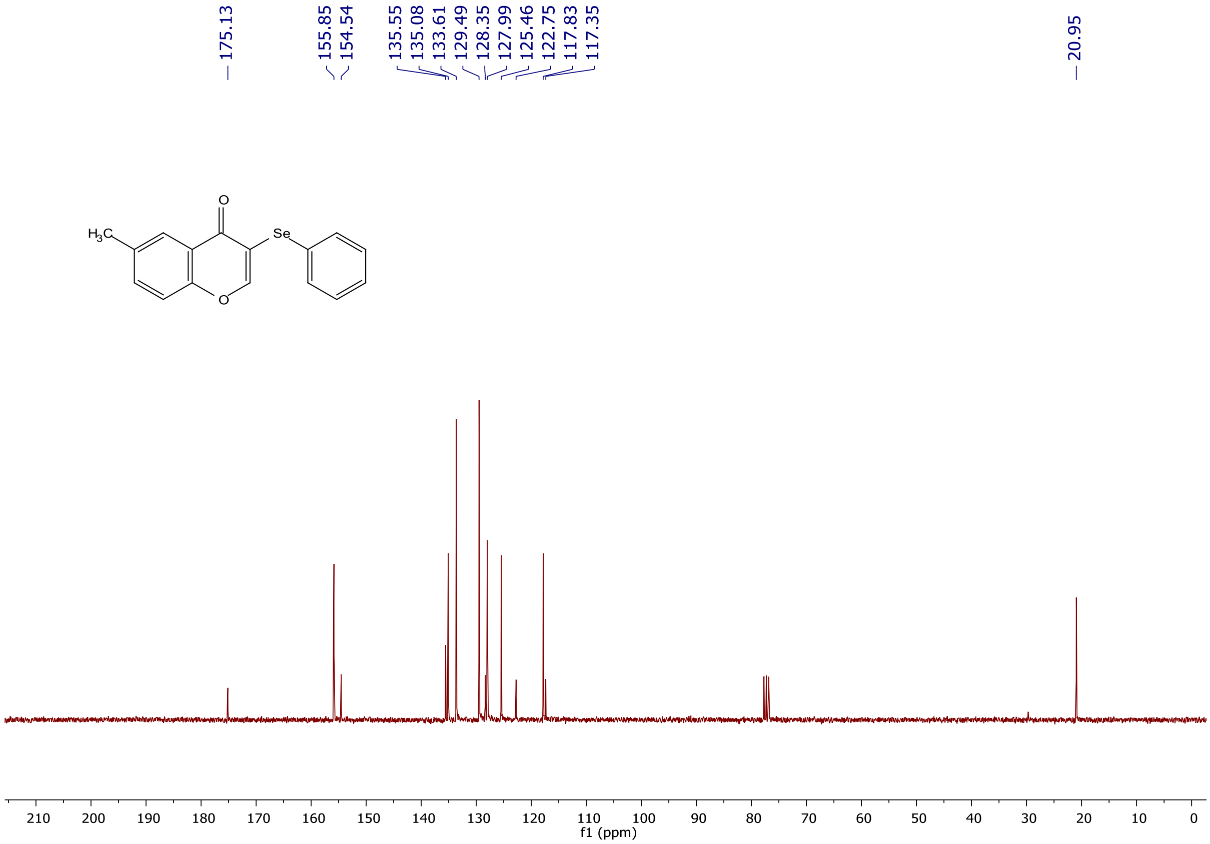

Figure S34. ^{13}C-NMR Spectra of the compound **4q**, in $CDCl_3$, 75 MHz

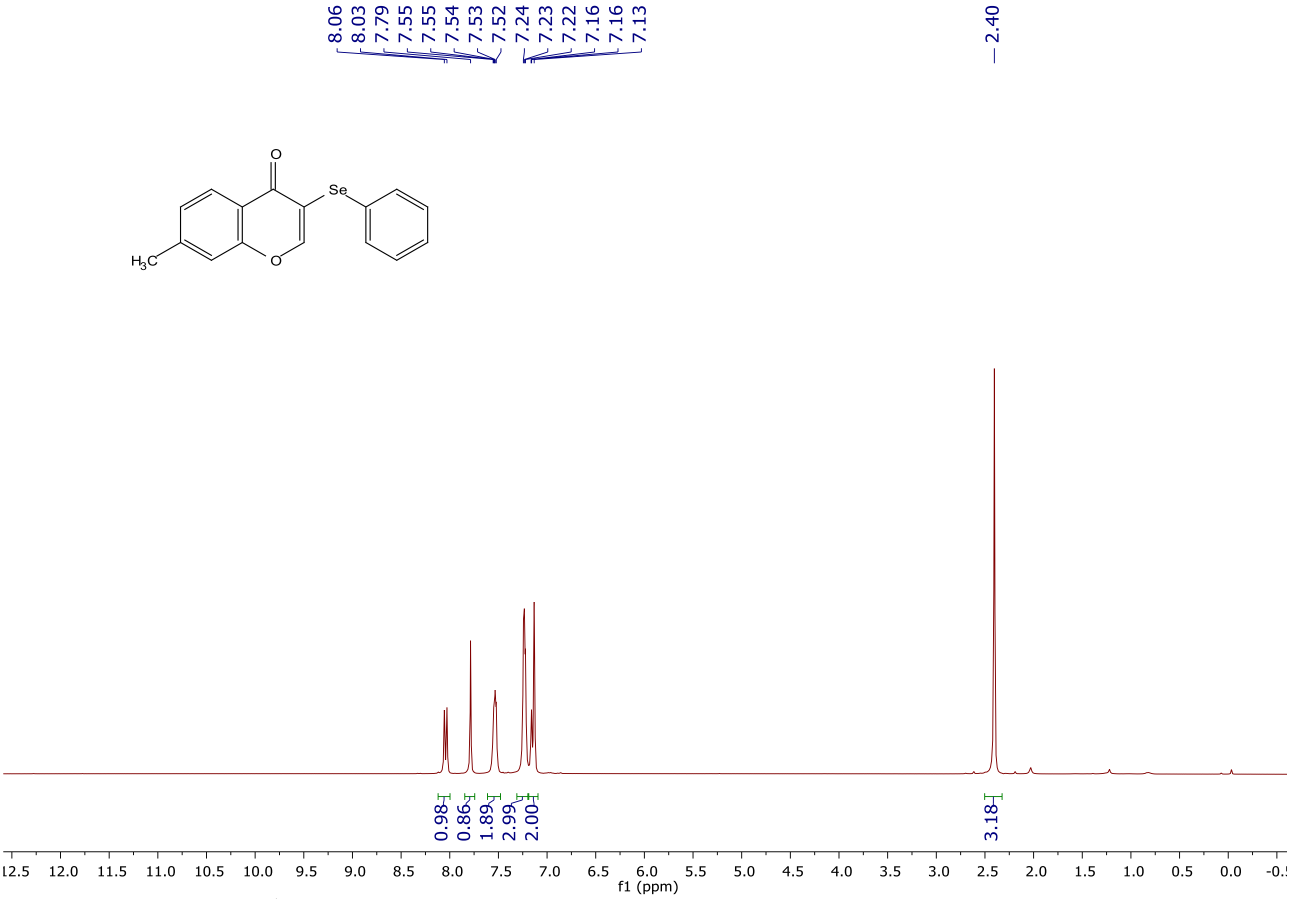

Figure S35. ^{1}H-NMR Spectra of the compound **4r,** in $CDCl_3$, 300 MHz

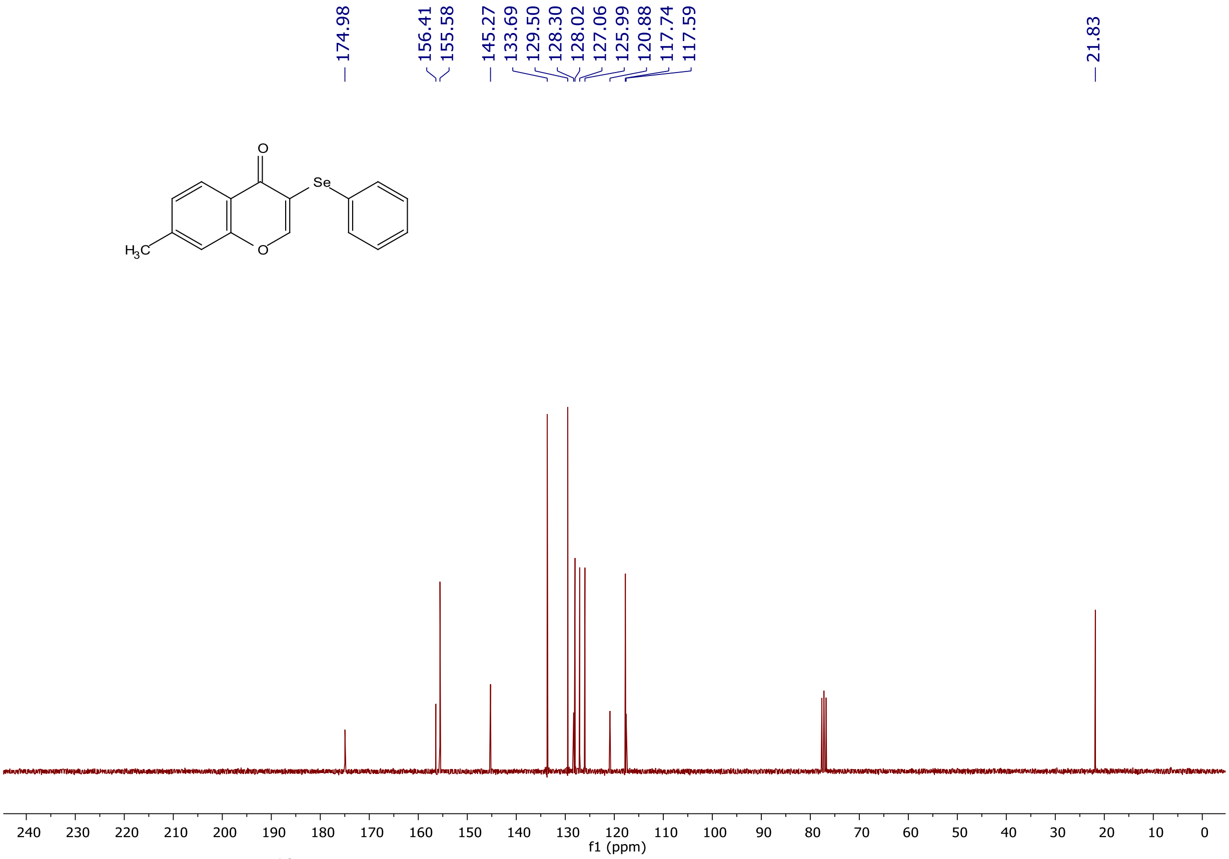

Figure S36. ^{13}C-NMR Spectra of the compound **4r**, in $CDCl_3$, 75 MHz

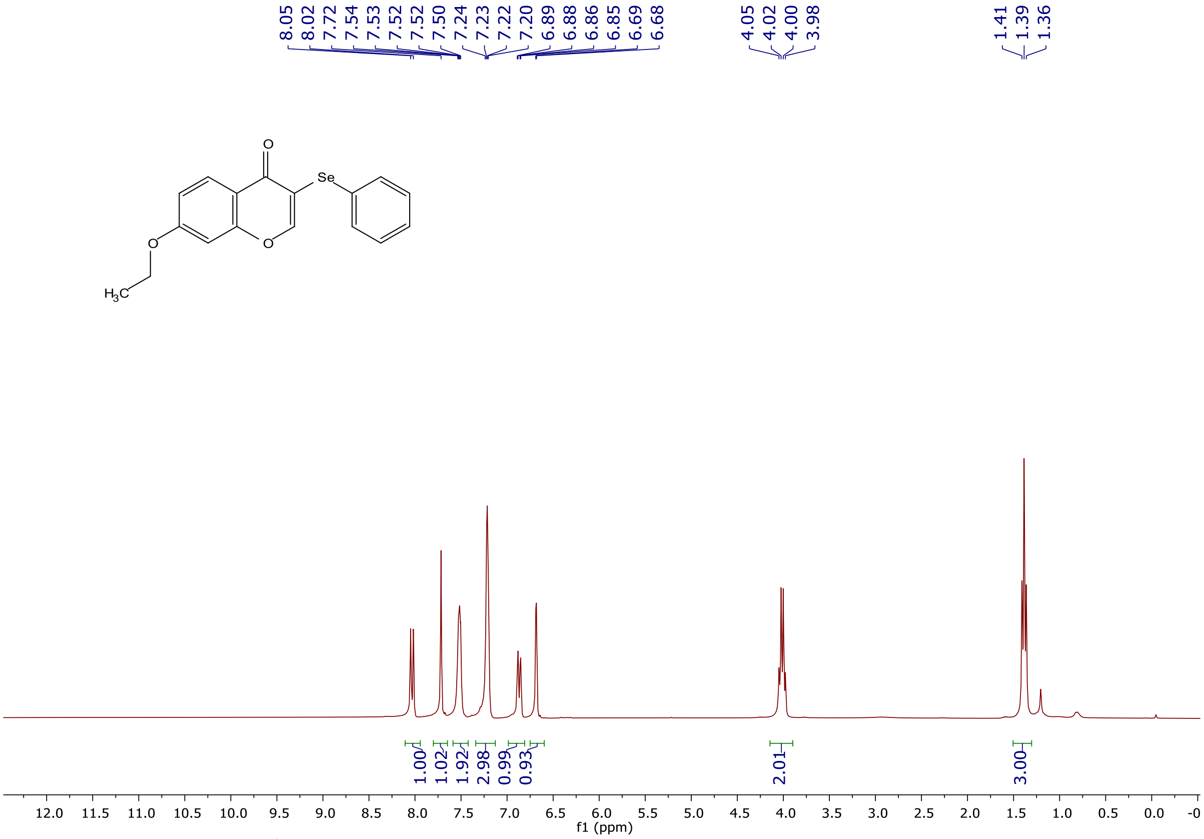

Figure S37. ^{1}H-NMR Spectra of the compound **4s,** in $CDCl_3$, 300 MHz

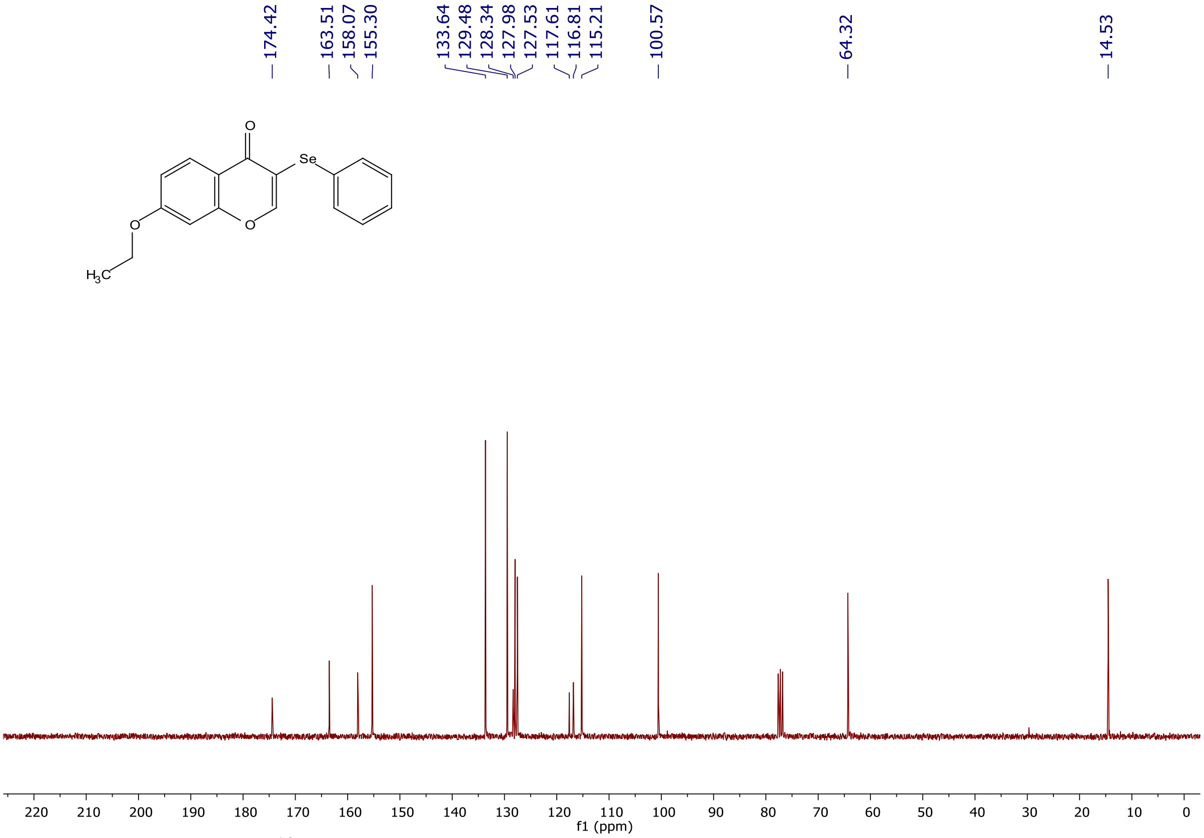

Figure S38. ^{13}C-NMR Spectra of the compound **4s**, in $CDCl_3$, 75 MHz

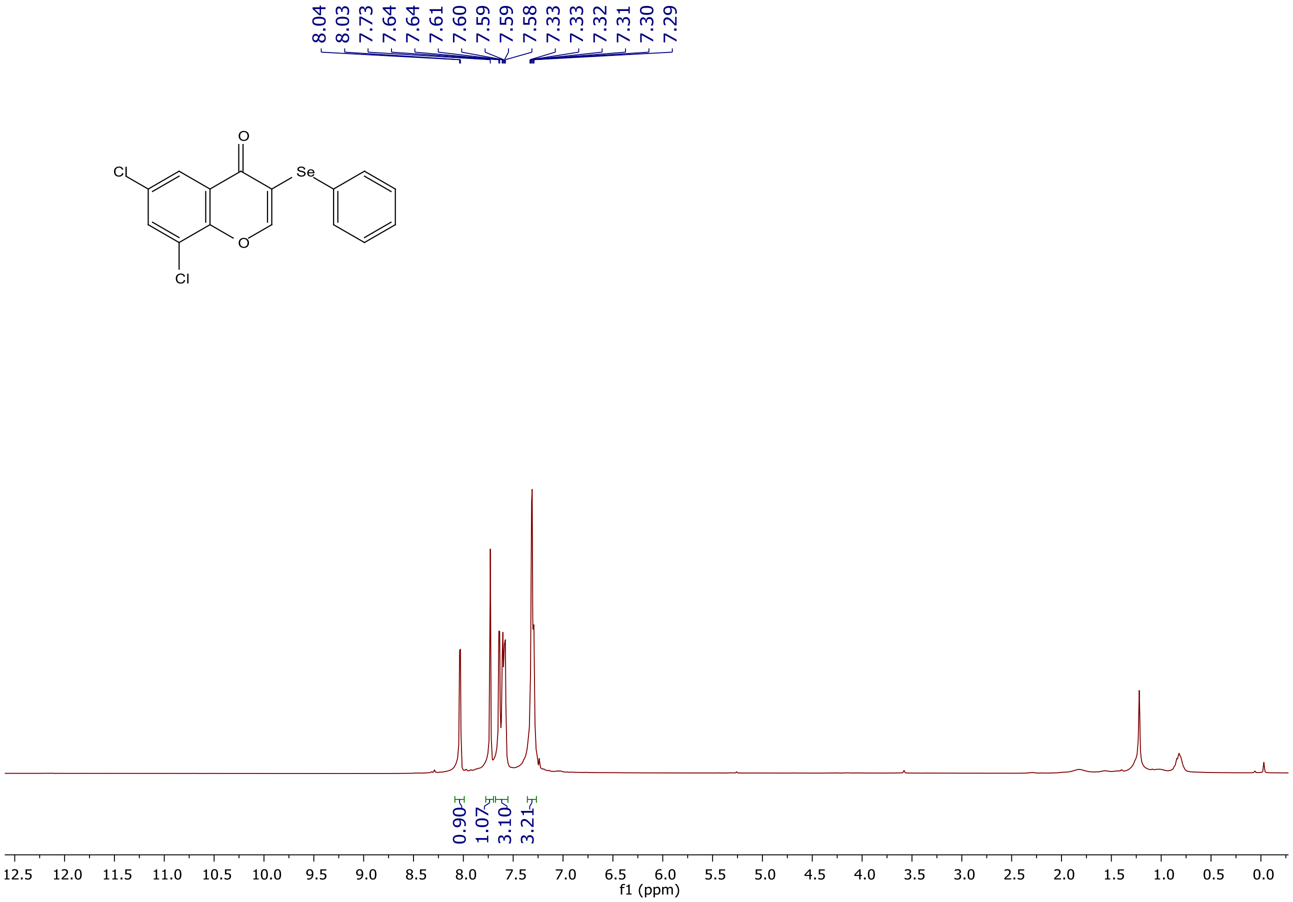

Figure S39. ^{1}H-NMR Spectra of the compound **4t,** in $CDCl_3$, 300 MHz

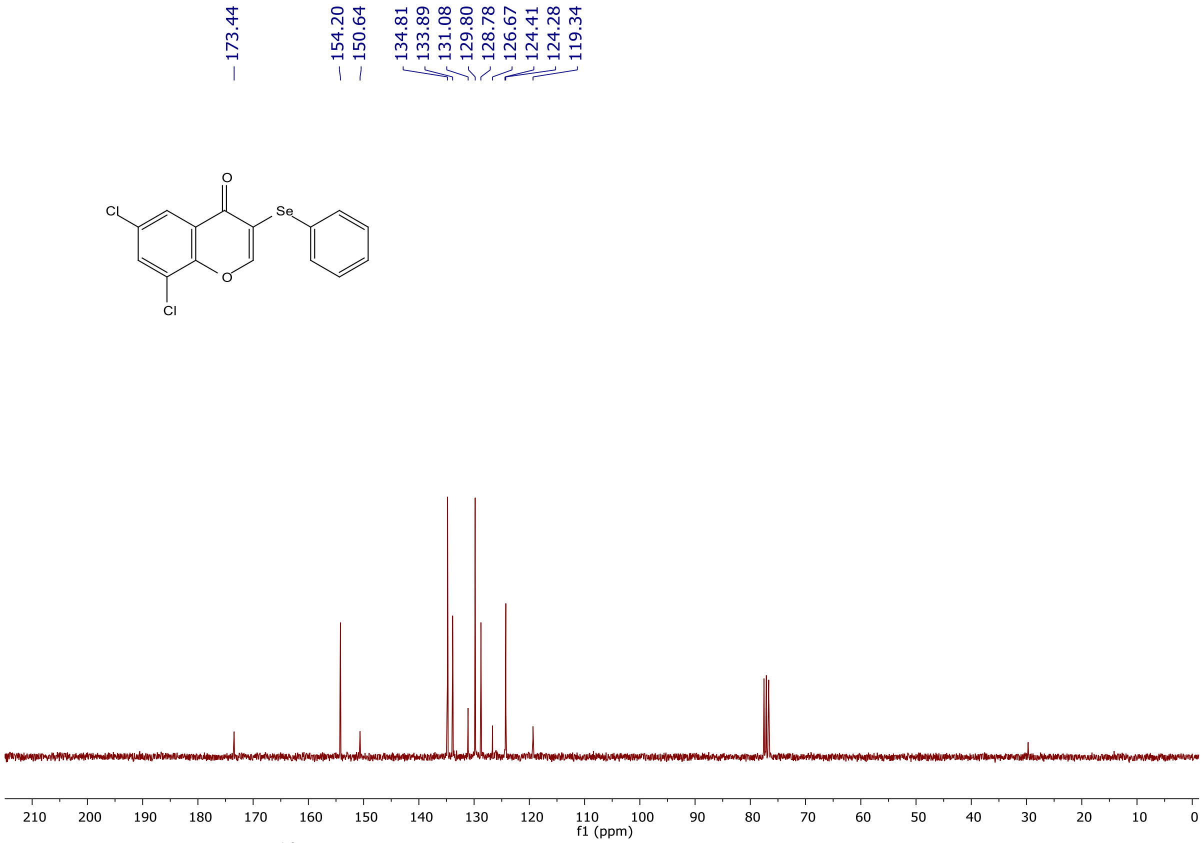

Figure S40. ^{13}C-NMR Spectra of the compound **4t**, in $CDCl_3$, 75 MHz

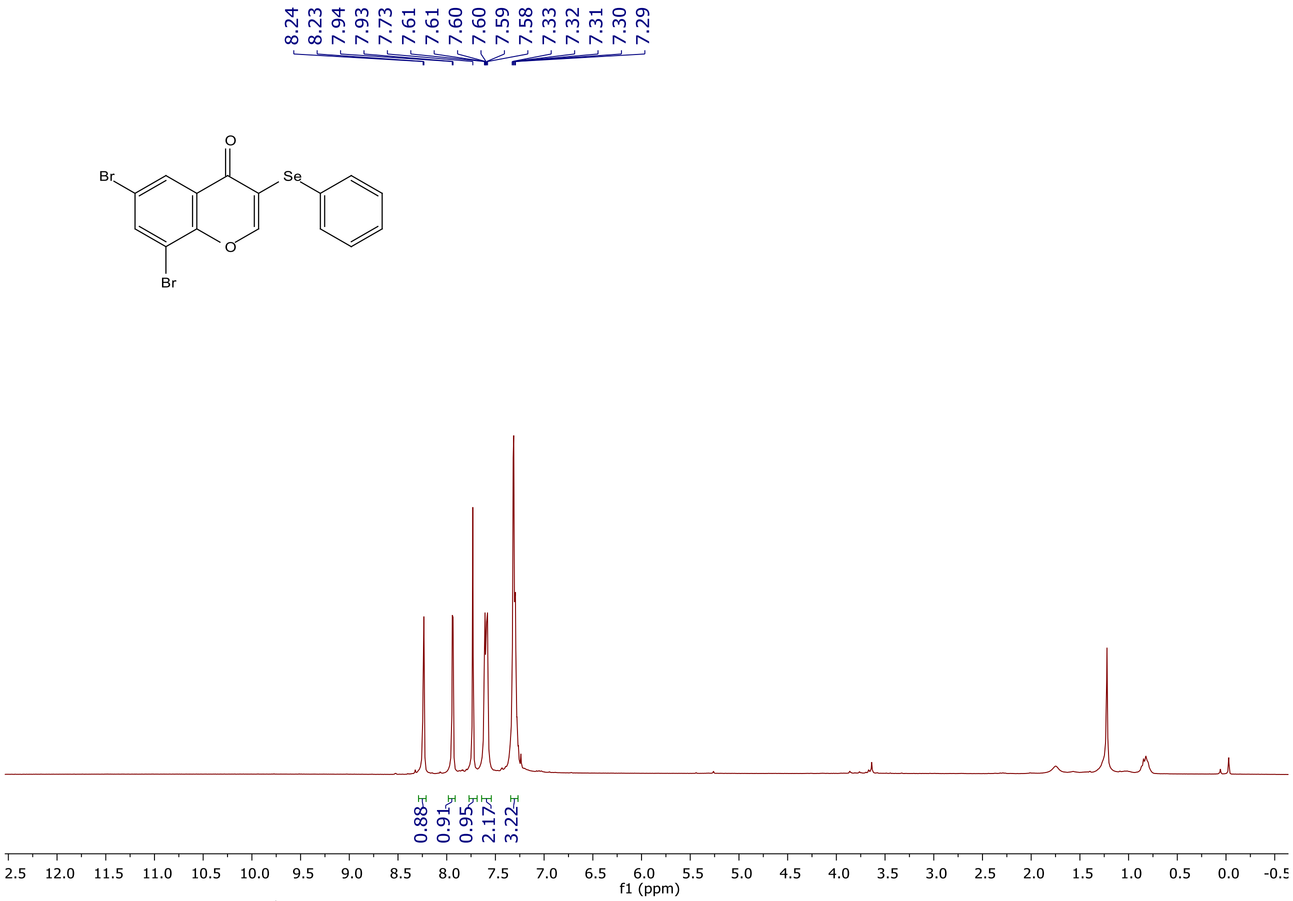

Figure S41. ^{1}H-NMR Spectra of the compound **4u,** in $CDCl_3$, 300 MHz

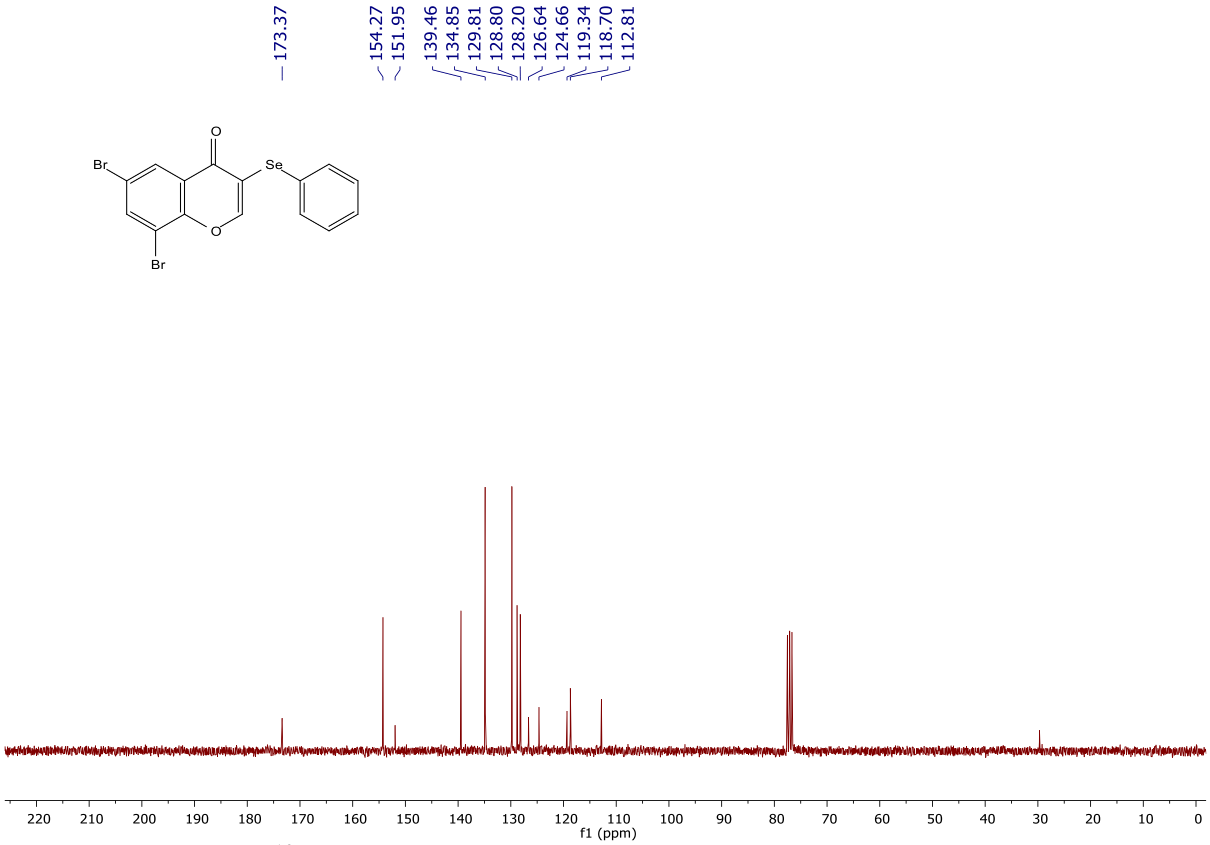

Figure S42. ^{13}C-NMR Spectra of the compound **4u**, in $CDCl_3$, 75 MHz

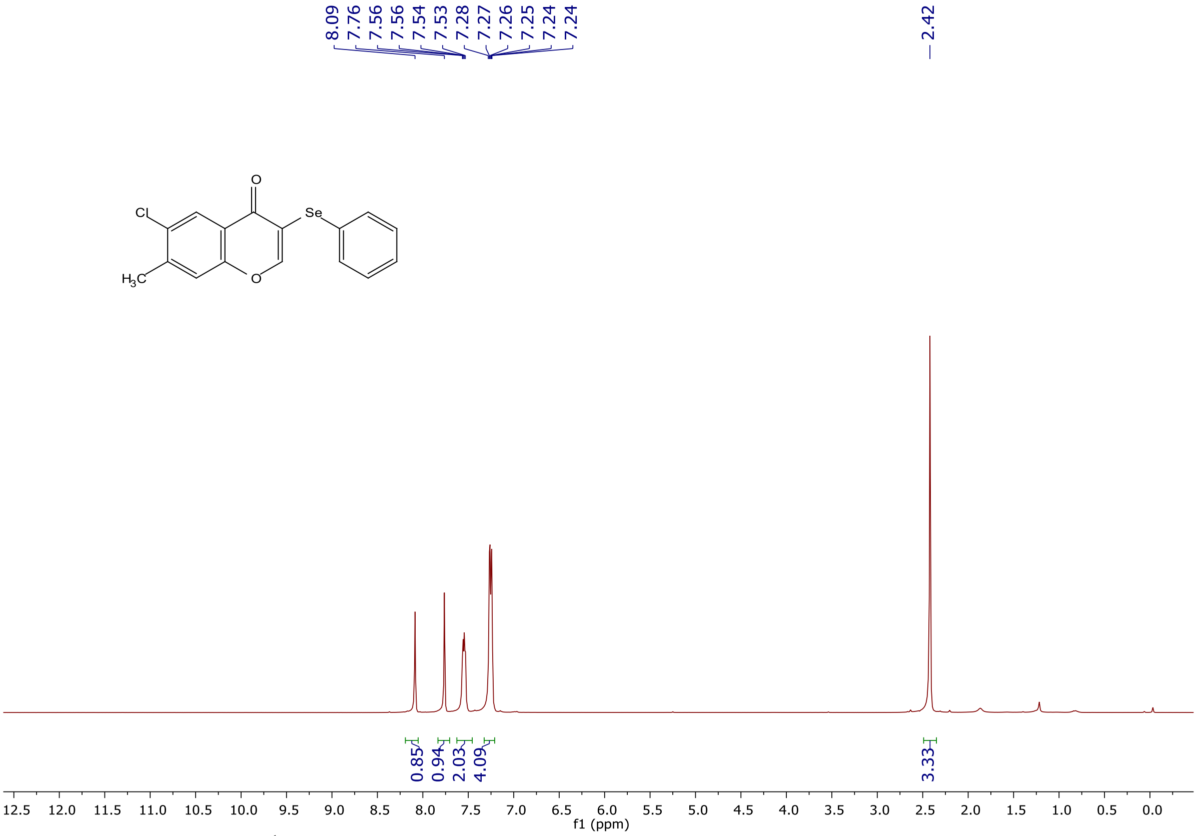

Figure S43. ^{1}H-NMR Spectra of the compound **4v,** in $CDCl_3$, 300 MHz

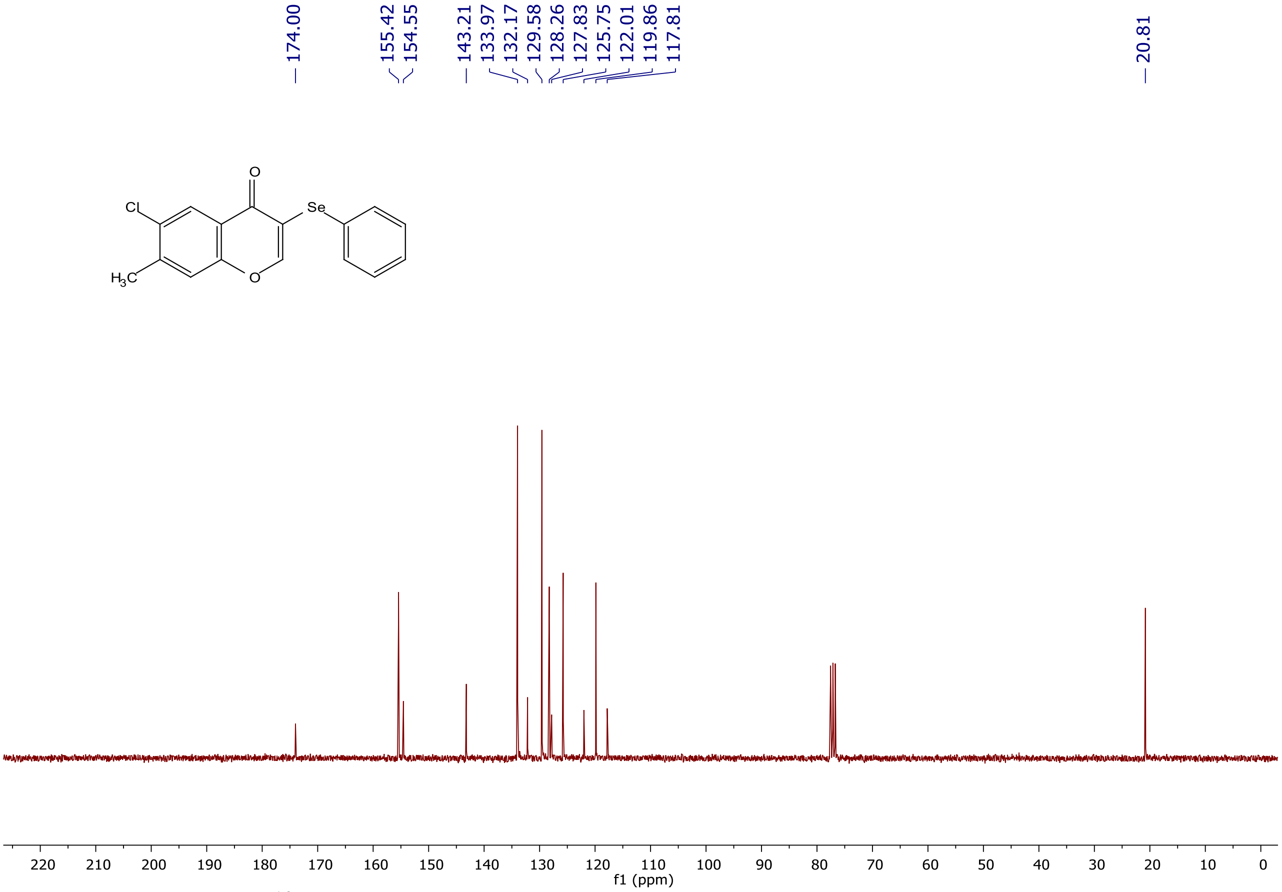

Figure S44. ^{13}C-NMR Spectra of the compound **4v**, in $CDCl_3$, 75 MHz

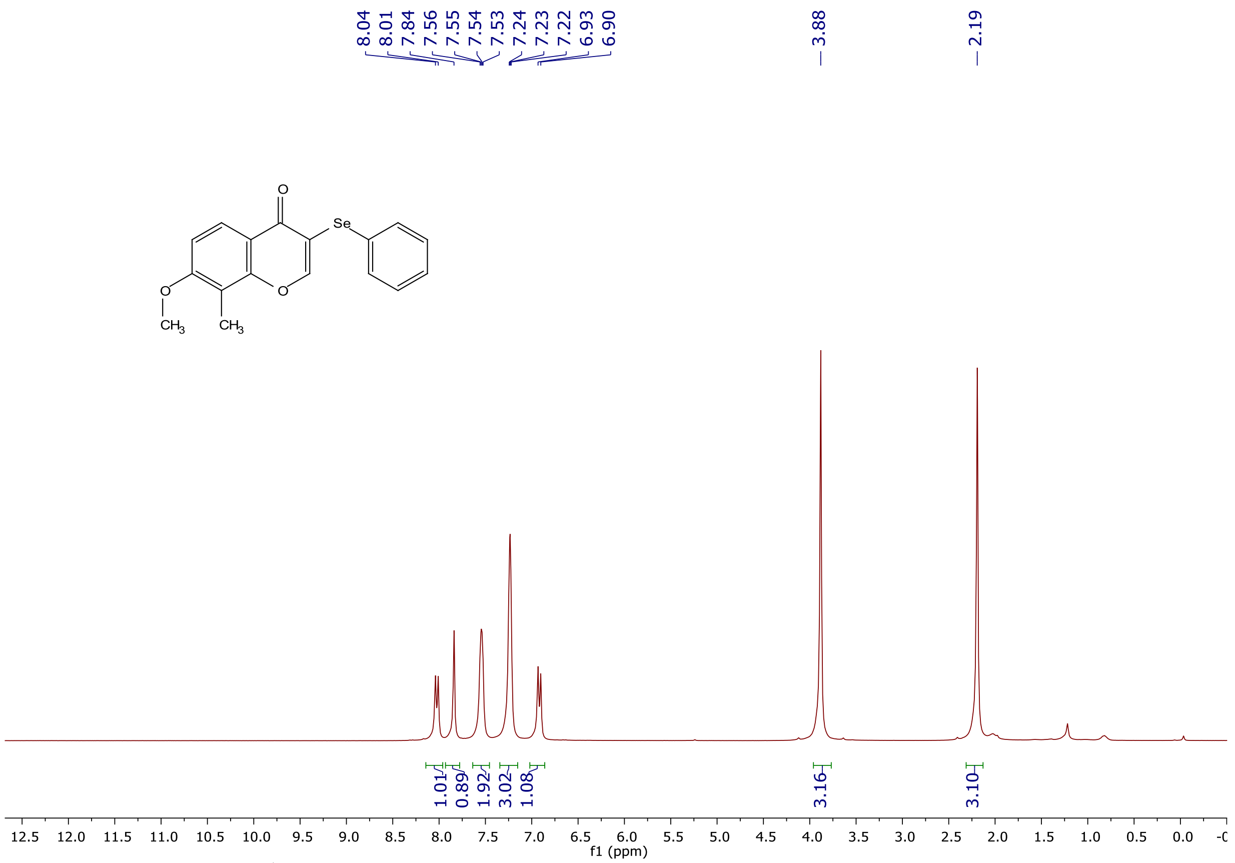

Figure S45. ^{1}H-NMR Spectra of the compound **4w,** in $CDCl_3$, 300 MHz

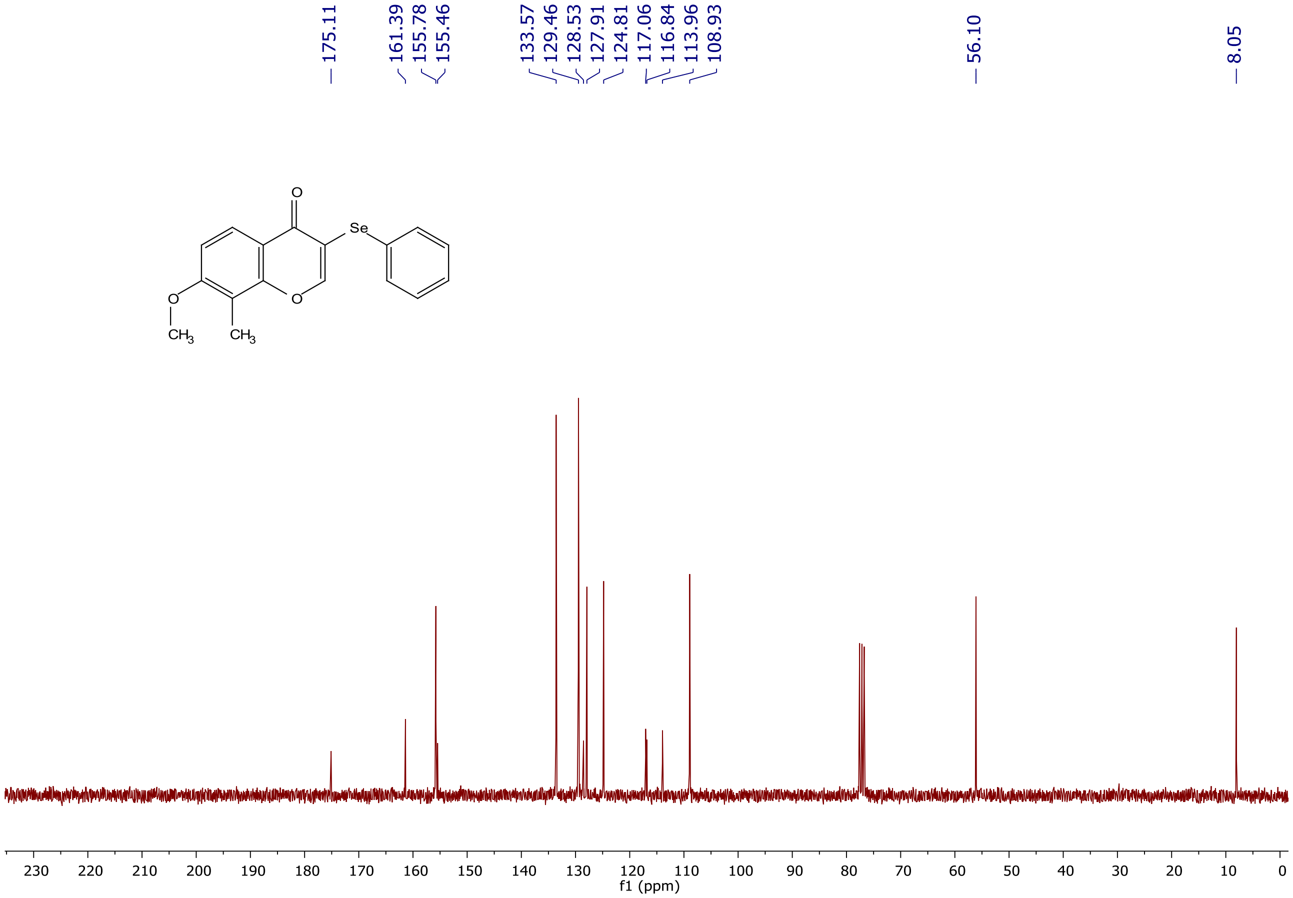

Figure S46. ^{13}C-NMR Spectra of the compound **4w**, in $CDCl_3$, 75 MHz

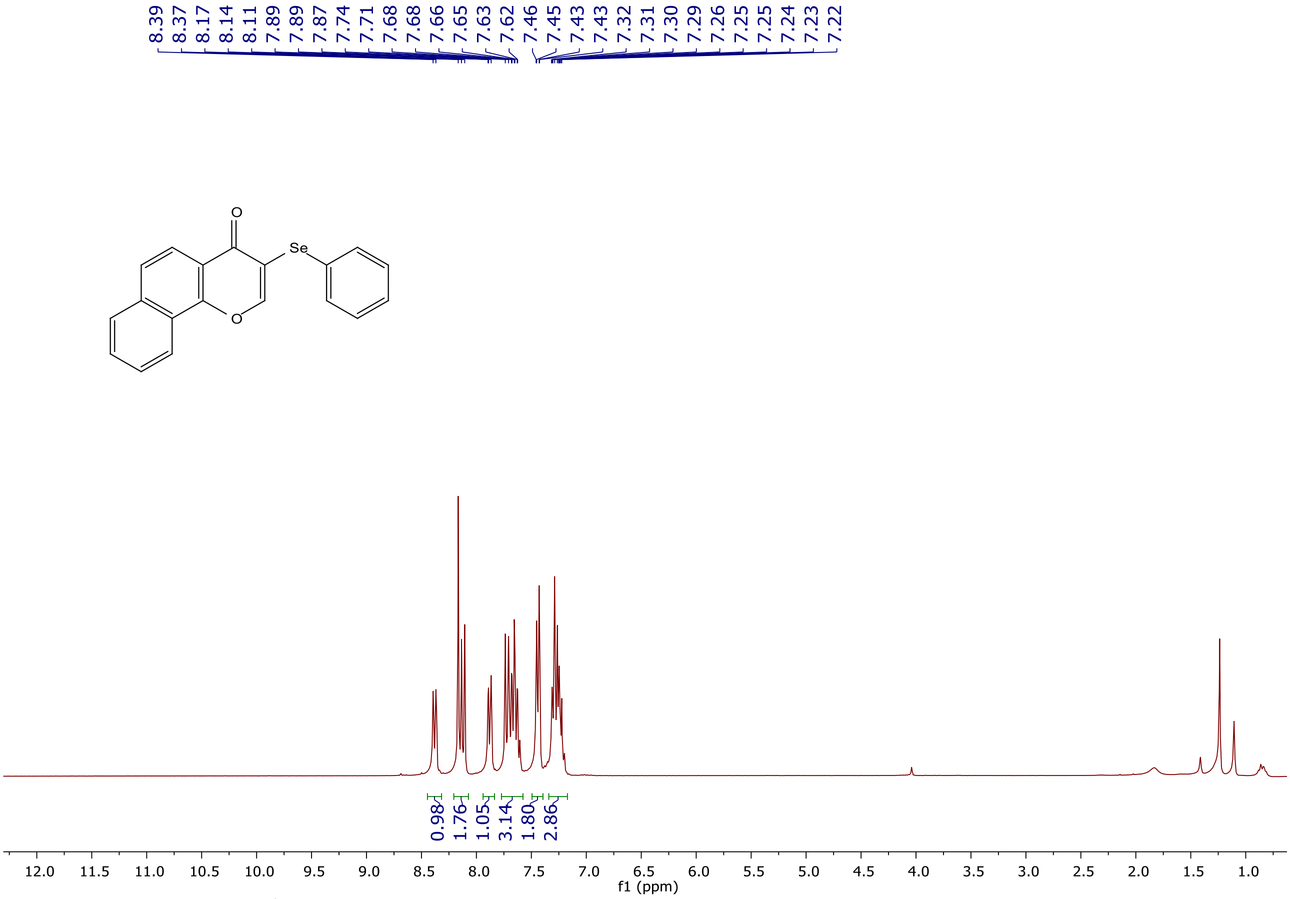

Figure S47. ^{1}H-NMR Spectra of the compound **4x,** in $CDCl_3$, 300 MHz

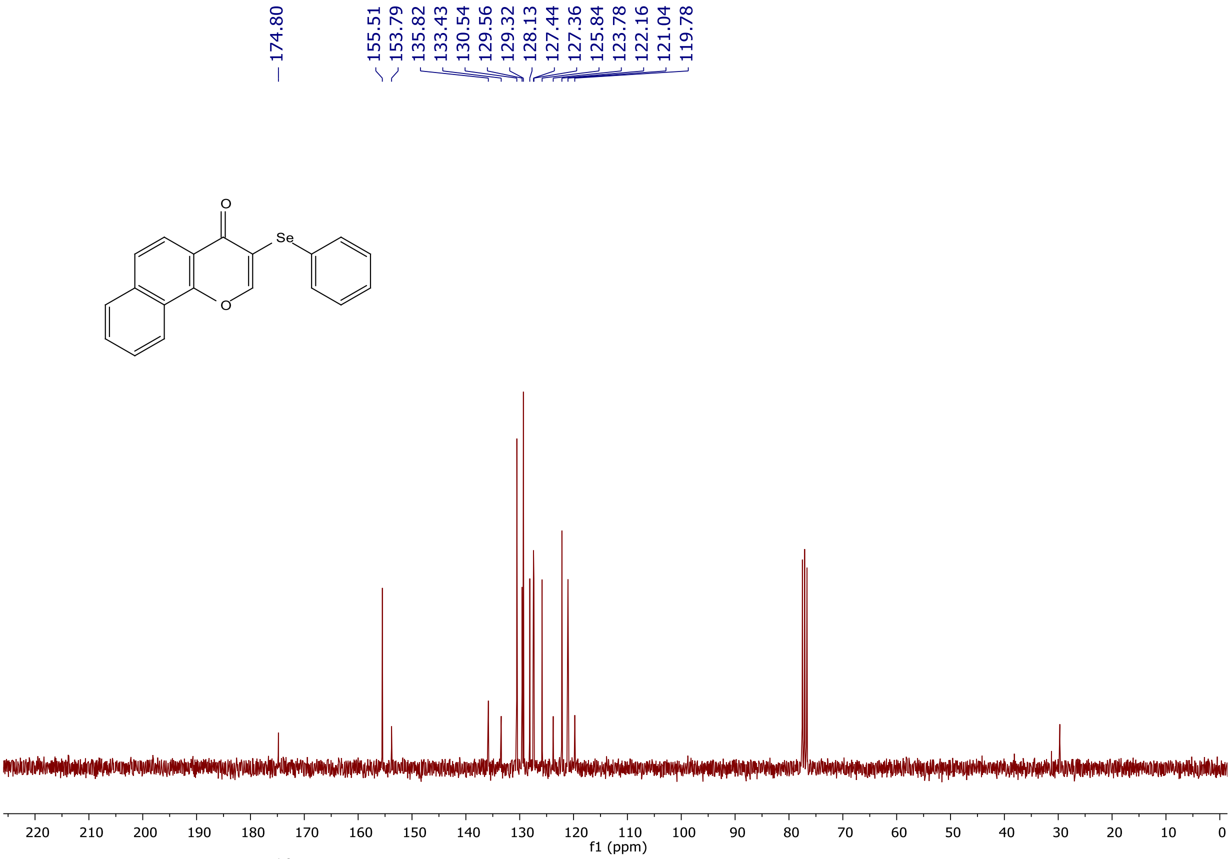

Figure S48. ^{13}C-NMR Spectra of the compound **4a**, in $CDCl_3$, 75 MHz
